# Sumac (*Rhus coriaria* L.) Capsules for the management of hypermenorrhea: Protocol for a randomized triple-blind trial

**DOI:** 10.1371/journal.pone.0361956

**Published:** 2026-11-11

**Authors:** Morteza Mojahedi, Adeleh Rezaei, Romina Fili, Fereshteh Behmanesh, Zahra Memariani, Maryam Nikpour, Sedigheh Esmaeilzadeh, Sobhan Rahimi Osbo

**Affiliations:** 1 Traditional Medicine and History of Medical Sciences Research Center, Health Research Institute, Babol University of Medical Sciences, Babol, I.R. Iran; 2 Student Research Committee, Babol University of Medical Sciences, Babol, I.R. Iran; 3 Student Research Committee, Babol University of Medical Sciences, Babol, I.R. Iran; 4 Infertility and Reproductive Health Research Center, Health Research Institute, Babol University of Medical Sciences, Babol, I.R. Iran; 5 Traditional Medicine and History of Medical Sciences Research Center, Health Research Institute, Babol University of Medical Sciences, Babol, I.R. Iran; 6 Non-Communicable Pediatric Diseases Research Center, Health Research Institute, Babol University of Medical Sciences, Babol, I.R. Iran; 7 Infertility and Reproductive Health Research Center, Health Research Institute, Babol University of Medical Sciences, Babol, I.R. Iran; 8 Social Determinants of Health Research Center, Health Research Institute, Babol University of Medical Sciences, Babol, I.R. Iran; Longhua Hospital Shanghai University of Traditional Chinese Medicine, CHINA

## Abstract

**Background:**

Hypermenorrhea is a common gynecological disorder adversely affecting women’s quality of life. Conventional treatments, particularly hormonal therapies, are associated with side effects and contraindications. *Rhus coriaria L.* (sumac) has a historical basis in Persian medicine for its astringent and haemostatic properties, supported by modern phytochemical evidence of tannins and flavonoids. However, robust clinical evidence for its efficacy in hypermenorrhea is lacking. This study aims to evaluate the efficacy and safety of sumac capsules compared to a placebo in reducing menstrual blood loss in women with hypermenorrhea.

**Methods:**

A triple-blind, parallel-group, randomized, placebo-controlled clinical trial will be conducted. Sixty eligible women aged 18–45 years with hypermenorrhea (Pictorial Blood Loss Assessment Chart [PBAC] score ≥100) will be randomly allocated to either the intervention group (500 mg sumac capsule twice daily for the first 7 days of menstruation) or the control group (Avicel placebo capsule), over three consecutive menstrual cycles.

**Discussion:**

This protocol details a methodologically rigorous trial to generate high-level evidence on the potential of sumac as a complementary treatment for hypermenorrhea. If proven effective, sumac could offer a safe, accessible, and cost-effective therapeutic alternative, aligning with patient preferences for natural interventions. The results will inform future clinical practice and research in integrative women’s health.

**Trial registration:**

Iranian Registry of Clinical Trials (IRCT), IRCT20180218038783N12, registered on 5 October 2025.

## Introduction

Abnormal menstrual bleeding is a medical condition characterized by irregular menstrual cycles, including excessive menstrual blood loss. Menstrual bleeding exceeding 80 milliliters per cycle or lasting longer than seven days is considered abnormal, a condition referred to as hypermenorrhea [1,2]. The prevalence of abnormal menstrual bleeding worldwide varies and depends on multiple factors, including geographical region, cultural context, socioeconomic status, access to and effectiveness of medical care, and environmental factors [1,3].

Complications of abnormal menstrual bleeding include iron deficiency, physical symptoms such as pain and fatigue, and, consequently, a reduced quality of personal and social life among women of reproductive age [4].

The most common causes of abnormal menstrual bleeding include pregnancy-related conditions, structural uterine pathologies (e.g., fibroids, polyps, adenomyosis), ovulatory disorders, and neoplasms. Excessive blood loss from these conditions can lead to anemia, coagulopathy, acidosis, hypothermia, and coagulation abnormalities [4,5]. Hormonal disorders and platelet-related blood abnormalities, such as vascular diseases and coagulation disorders, can also influence this condition [6,7].

First-line treatment for hypermenorrhea is pharmacological therapy, including nonsteroidal anti-inflammatory drugs, hormone therapy, progestins, and progesterone-releasing intrauterine devices. Surgical approaches such as hysterectomy or endometrial ablation are reserved for inadequate response to medical therapy [8]. In some cases, emergency treatment is required, such as in the presence of severe bleeding, which may result from acute blood loss (hemorrhage) [9,10].

In traditional Iranian medicine texts, excessive menstrual flow, prolonged menstrual duration, and bleeding outside the menstrual period are referred to as efrāṭ-e ṭamth or kasrat-e ṭamth [11]. In traditional Iranian medicine, the general principles of treatment in traditional Iranian medicine are based on lifestyle modification, with nutrition considered a key component. If no improvement is observed, the next steps recommended are pharmacological treatment followed by manual or procedural interventions [12]. The efficacy of certain pharmacological agents, including mefenamic acid, tranexamic acid, ethamsylate, and oral contraceptives, has been investigated in clinical trials, which have demonstrated positive effects [13]. In the first step, hematinic treatments consisting of Khabth al-Hadid (iron-based preparations) derivatives are prescribed to the patient. In the second step, herbal astringents such as quince paste, myrtle, pomegranate flower, and plantain are administered, depending on availability and cost-effectiveness [11,14].

In traditional Iranian medicine, sumac has been recommended for the management of abnormal uterine bleeding because of its cooling and astringent properties. Traditional sources describe excessive bleeding as being associated with increased heat and fluidity of blood, for which cooling and astringent foods such as sumac, pomegranate, barberry, and vinegar have been recommended to help reduce bleeding and constrict blood vessels [15].

Sumac is a plant from the pistachio family, with the scientific name Rhus coriaria L [16,17]. The flowers of the sumac shrub are dense, clustered, and honey-colored. Sumac primarily grows in rocky areas and on hard, rugged soils [18]. Sumac has a cold and dry nature (second degree) with astringent, stomach-tonic, and bile-reducing properties. It has traditionally been used for nausea and vomiting caused by bile imbalance, bleeding, and chronic diarrhea [19].

In traditional Iranian medicine, sumac has been regarded as a preventive agent against cardiovascular diseases [20]. Many recent findings regarding the effects of sumac on digestive disorders, diarrhea, as well as its antiseptic and analgesic properties, are consistent with the observations of traditional Iranian medicine scholars [21]. Sumac has also long been used as a food seasoning and for strengthening the stomach and gums, reducing or stopping bleeding, and preventing infectious diseases [22].

One of the therapeutic uses of sumac in traditional Iranian medicine is its application in the preparation of sumac porridge (Somāqīyeh), which has been used to treat conditions such as indigestion and diarrhea [23], nausea, nosebleeds, blood-related joint pain, hiccups, and bleeding from hemorrhoidal vessels [24]. According to written Iranian medical sources, this plant has also been used for uterine disorders [25,26]. Studies have shown that sumac increases platelet distribution width [27]. It can also influence bleeding time, prothrombin time, and activated partial thromboplastin time, suggesting its potential as an adjuvant therapy for bleeding control. Salsali and colleagues reviewed classical Iranian medical texts including Qanun fi al-Tibb, Tibb Akbari Arzani, Ma‘alijat Aghili Khorasani, Sharh al-Asbab Samarkandi, and Aksir-e Azam Nazem Jahan, and reported that astringent foods such as sumac can be effective in reducing menstrual bleeding [15].

The active compound in sumac is tannins [28]. Sumac is also rich in magnesium, calcium, tannins, flavonoids, and other polyphenolic compounds. Its astringent properties are attributed to malic acid, tannic acid, gallic acid, and tannins. Tannins and flavonoids stimulate estrogen receptors, and uterine vascular contraction [14]. Experimental studies suggest that polyphenols and tannins in Rhus coriaria may influence inflammatory and vascular pathways, but their relevance to human uterine bleeding remains uncertain and requires clinical confirmation [29].

Experimental studies suggest that Rhus coriaria may exert anti-inflammatory and antioxidant effects through modulation of inflammatory cytokines and oxidative stress markers [30]. Since inflammatory pathways may contribute to abnormal uterine bleeding, these findings provide a possible biological rationale for investigating the effect of sumac on hypermenorrhea. However, direct clinical evidence in this field is still lacking [31,32]. Recent systematic reviews continue to emphasize the need for well-designed randomized trials to evaluate herbal interventions for menstrual bleeding disorders [32].

Given references in traditional medicine texts to the potential pro-coagulant effects of *Rhus coriaria* and the hemostatic activity, well-designed human trials are required to clarify its clinical effects on menstrual bleeding. Therefore, this trial aims to evaluate, based on the PICO framework, whether sumac capsules reduce menstrual blood loss in women with hypermenorrhea compared to placebo.

### Objectives

The primary objective of this trial is to evaluate whether oral administration of Rhus coriaria capsules reduces menstrual blood loss (measured by changes in PBAC score) and bleeding duration (number of bleeding days) compared with placebo over three consecutive menstrual cycles. Secondary objectives include assessing the safety and tolerability of sumac capsules and recording the frequency and type of adverse events during the intervention period.

## Methods

### Study design and setting

This study will be conducted as a triple-blind, parallel-group randomized clinical trial with random allocation of participants into two groups: intervention and control. This manuscript follows the SPIRIT 2013 guidelines (Supplementary file 1). The trial was registered in the Iranian Clinical Trial Registry (Code: IRCT20180218038783N12) on 5 October 2025. Recruitment commenced on 22 October 2025 and is currently ongoing. At the time of manuscript revision, 20 participants had been recruited and randomized, and recruitment will continue until the target sample size of 60 participants is reached. Participants will be recruited from a traditional medicine clinic, a gynecology clinic, and the female dormitory of Babol University of Medical Sciences. The combination of clinical and non-clinical recruitment sources enhances the diversity of the sample. The SPIRIT schedule of enrolment, interventions, assessments, and visits for participants is shown in Fig 1.

**Fig 1:**
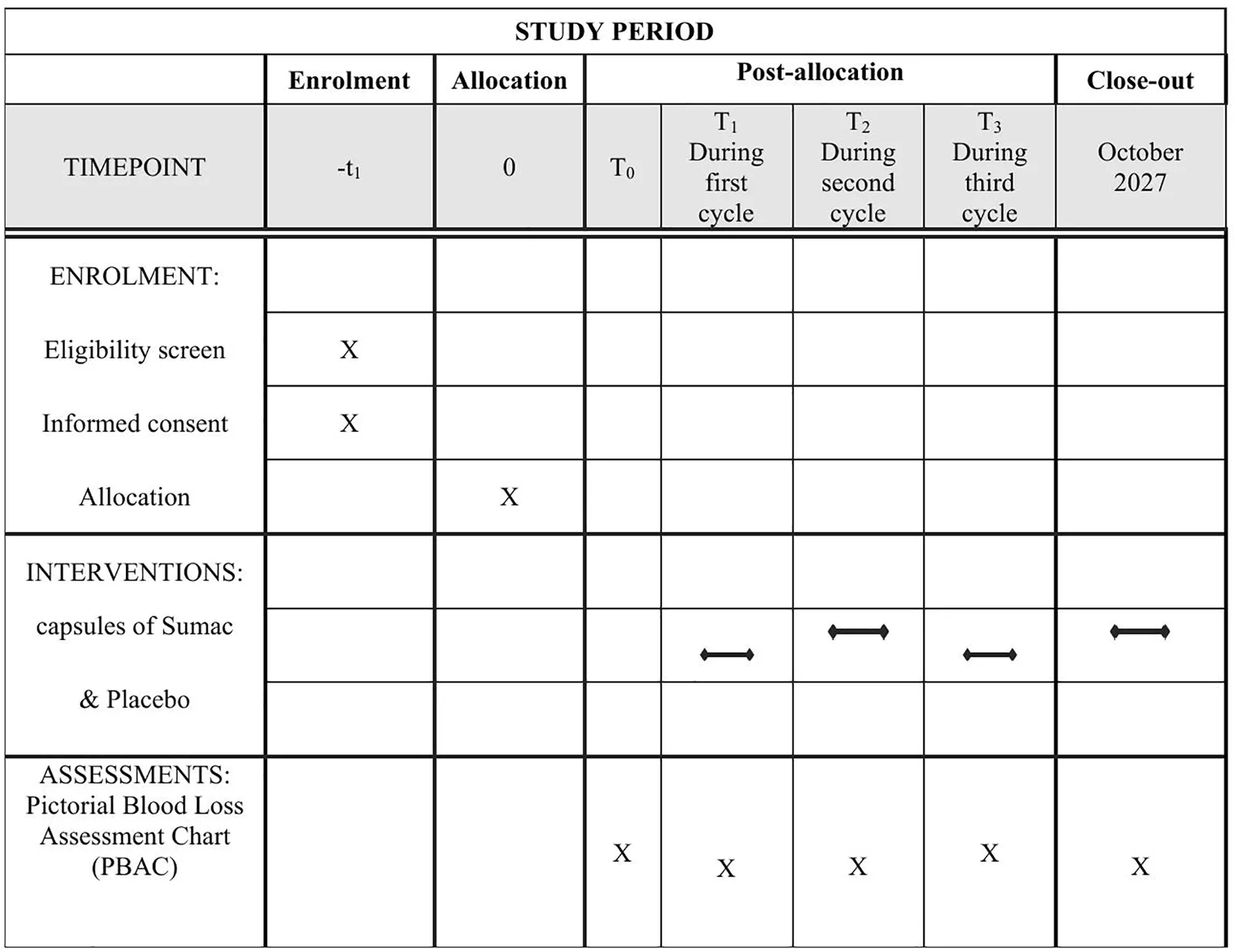
SPIRIT schedule of enrolment, interventions, and assessments. The schedule follows the SPIRIT 2013 Statement and presents the timing of enrolment, allocation, interventions, and outcome assessments.

The flow of participants through the study is illustrated in Fig. 2.

**Fig 2:**
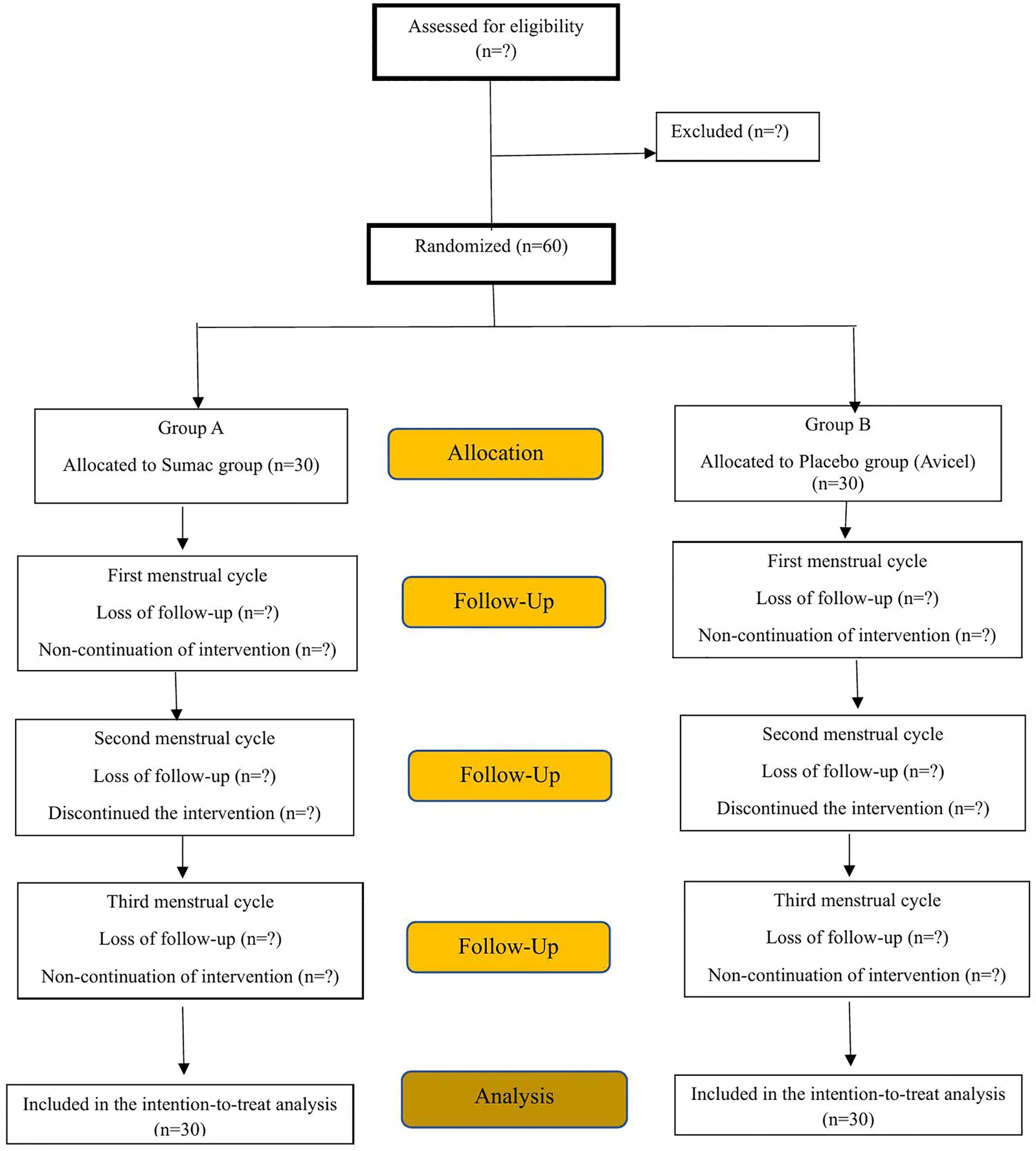
Flow of participants through the study.

### Eligibility criteria

Eligible participants will include women aged 18–45 years with menstrual blood loss >80 mL per cycle, assessed using the Pictorial Blood Loss Assessment Chart (PBAC) (score ≥100), consent to participate in study, a regular menstrual cycle of 21–42 days, and literacy.

Exclusion criteria will include pregnancy or breastfeeding at the time of enrollment, prior ultrasound report showing uterine fibroids >3 cm (submucosal or intramural), endometrial polyps, ovarian cysts, known neoplasms, irregular menstrual cycles, chronic medical conditions (hypertension, uncontrolled diabetes, renal, hepatic, cardiovascular, or thyroid disorders), use of anticoagulants or hormonal contraceptives, smoking or alcohol consumption, or abnormal endometrial thickness, first-degree family history of cancer, known allergy to sumac, adherence to a special diet (e.g., very low-calorie diet, ketogenic diet, vegan/vegetarian diet, or regular consumption of herbal supplements with hormonal/hemostatic effects) in the previous three months, and severe bleeding requiring hospitalization (hemodynamic instability needing IV fluids or transfusion, per physician judgment). Withdrawal criteria will include <80% treatment adherence, occurrence of pregnancy during the study period, and unwillingness to continue participation.

### Sample size

The sample size was determined to detect a clinically meaningful difference in the PBAC score, with calculations based on expert clinical judgment and related literature. An estimated standard deviation (SD) of 11.0 and a minimum detectable difference of 10.0 points were used. These parameters were derived considering variability from previous studies on heavy menstrual bleeding [33,34], aiming for a conservative estimate. The sample size was calculated using the formula for comparing two independent means:


$$\begin{array}{c} \text{n}=\frac{\text{2}(Z\text{1}-\alpha /\text{2}+Z\text{1}-\beta )\text{2}\sigma \text{2}}{d\text{2}} \end{array}$$

With α = 0.05\alpha = 0.05α = 0.05 (Z-score 1.96) and 90% power (Z-score 1.28), this yielded a required sample size of 26 participants per group. To account for an approximate 10%–15% anticipated loss to follow-up, the final sample size was adjusted to 30 participants per group, for a total of 60 participants.

### Randomization, allocation concealment, and blinding

Eligible participants will be recruited consecutively from patients presenting to the study clinic who meet the inclusion criteria. They will then be randomly assigned in a 1:1 ratio to the intervention or placebo group using block randomization (block size = 10) generated by Random Allocation software. Allocation concealment will be ensured using sequentially numbered, opaque, sealed envelopes prepared by an independent individual. Random sequence generation, participant enrollment, and group assignment will be performed by separate personnel. Participants, care providers responsible for capsule administration, and the data analyst will be blinded to group allocation. The placebo and sumac capsules are identical in shape, color, size, texture, taste, and packaging. Randomization codes will be kept in the sealed envelopes and broken only after the final data analysis is completed.

### Implementation

After selecting participants based on the inclusion criteria and obtaining informed consent, they will be randomly allocated into two groups (Sumac and Placebo). The use of an inert placebo as the comparator will allow the specific effect of sumac supplementation on menstrual blood loss to be distinguished from nonspecific effects related to participant expectations and trial participation. The intervention group will receive capsules containing an aqueous extract of sumac fruit (A++ grade), obtained from Giah Kala Company, with the total flavonoid content determined. Each capsule will be equivalent to 500 mg of dried fruit. The control group will receive placebo capsules containing 500 mg of Avicel, purchased from Behboud Shimi Company (Gloria brand). All capsules (both sumac and placebo) were prepared by a single traditional medicine pharmacist who was not involved in participant recruitment, using identical capsules, equipment, and packaging procedures to ensure consistency. Participants will take two capsules daily, at 12-hour intervals, during the first seven days of menstruation, for three consecutive menstrual cycles. Adherence to the intervention will be monitored through participant-completed daily checklists and by checking returned capsule packaging at each monthly visit. Adherence will be calculated as the percentage of capsules taken relative to the expected total.

Mefenamic acid (or other painkillers) and iron supplements will be permitted as concomitant medications and will not be prohibited during the trial. Their use will be documented throughout the study, including the type, dose, frequency, and duration, using a daily checklist. The use of these concomitant medications may contribute to variability in menstrual blood loss. Therefore, their use will be considered in the statistical analysis using a linear mixed-effects model (LMM) to account for their potential effects on the primary outcome.

### Concomitant medications

Prohibited concomitant medications will include hormonal contraceptives, anticoagulants, and any herbal supplements with known hormonal or hemostatic effects. Permitted concomitant medications will include analgesics for dysmenorrhea and iron supplements. All concomitant medication use will be recorded in a daily checklist, including the type, dose, frequency, and duration of use.

### Primary and secondary outcome

The primary outcomes of this study are the intensity and duration of menstrual bleeding, assessed using the Pictorial Blood Loss Assessment Chart (PBAC). Bleeding intensity will be evaluated based on the total PBAC score, while bleeding duration will be determined by the number of bleeding days recorded in the PBAC chart. Outcomes will be assessed at baseline and after one, two, and three menstrual cycles following initiation of the intervention.

Secondary outcomes include the occurrence, type, and severity of any adverse effects associated with sumac capsule consumption, which will be recorded prospectively throughout the study period.

### Assessment and recording of adverse events

Adverse events will be assessed at each monthly follow-up visit through structured interviews and participant self-reporting. Participants will be instructed to report any gastrointestinal symptoms, allergic reactions, dizziness, nausea, or other unexpected symptoms during the intervention period.

Adverse events will be categorized according to severity (mild, moderate, severe) and their potential relationship to the intervention (unlikely, possible, probable). Serious adverse events will be reported immediately to the ethics committee.

### Instruments

Menstrual blood loss will be assessed using the PBAC (Higham Chart), a validated and reliable tool, completed at baseline and during each menstrual cycle. The Pictorial Blood Loss Assessment Chart (PBAC) was originally developed by Higham, O’Brien, and Shaw in 1990 as a semi-quantitative tool for assessing menstrual blood loss [35]. A PBAC score of ≥100 corresponds to a menstrual blood loss exceeding 80 mL. The Persian version has shown good reliability (r = 0.88) in the Iranian population [36]. Participants will complete the chart at baseline and during three menstrual cycles.

Demographic, socioeconomic, and obstetric data will be collected at baseline using a structured questionnaire including age, body mass index (BMI), educational level, place of residence, employment status, satisfaction with household income, and obstetric information such as the number of pregnancies, deliveries, abortions, and method of contraception. Data related to abnormal uterine bleeding included menstrual cycle characteristics, namely cycle regularity, intermenstrual interval, duration of bleeding, presence of dysmenorrhea, and volume of menstrual blood loss.

### Statistical analysis

All analyses will be conducted according to the intention-to-treat principle. Continuous variables will be summarized as means and standard deviations or medians and interquartile ranges, as appropriate. Changes in PBAC scores over time will be analyzed using linear mixed-effects models (LMMs), including group, time, and group-by-time interaction terms. Baseline covariates, including age, baseline PBAC score, and use of concomitant medications, will be included as fixed effects to improve precision. The primary inference will be based on the group-by-time interaction term. Between-group comparisons at individual time points will be performed using model-based estimates, with Bonferroni correction applied to control for multiple comparisons. If substantial deviations from the assumptions of the planned parametric analyses are identified, appropriate non-parametric methods will be considered, as applicable. LMMs use all available outcome data under the missing-at-random assumption, provided the model is correctly specified. If the proportion of missing data exceeds 10%, multiple imputation will be conducted as a sensitivity analysis. Effect estimates with 95% confidence intervals and two-sided p-values will be reported. A p-value < 0.05 will be considered statistically significant. Statistical analyses will be performed using SPSS software (version 26).

### Ethics and dissemination

Ethical approval for this study was obtained from the Research Ethics Committee of Babol University of Medical Sciences (Approval Code: IR.MUBABOL.REC.1404.106) on 4 August 2025. The use of an inert placebo as the control intervention is considered ethically acceptable because participants will not be receiving active treatment for heavy menstrual bleeding (HMB) at the time of enrollment. The placebo-controlled design will also allow the specific effect of sumac supplementation on menstrual blood loss to be distinguished from nonspecific effects related to participant expectations and trial participation. Participants who develop severe bleeding requiring hospitalization or other clinically important deterioration will be withdrawn from the study and referred for appropriate medical evaluation and treatment. Prior to participation, all potential participants will be comprehensively informed about the study aims, methodology, potential risks, and benefits. Enrollment will proceed only after written informed consent has been obtained from each participant. To safeguard participant privacy, all collected data will be anonymized using unique codes and stored securely. Participants will retain the right to withdraw from the study at any stage without consequence. The results will be published in a peer-reviewed journal and presented at relevant conferences. A plain-language summary of the findings will also be shared with participants upon request. Findings will additionally be disseminated to the public through conference presentations. We confirm that this manuscript, in whole or in part, is not being considered for publication elsewhere and has not been previously published.

### Study oversight and safety

Given the nature of the intervention (sumac capsule as a herbal product with a known safety profile), the limited sample size, and the short intervention period, the establishment of an independent Data Monitoring Committee (DMC) will not be required for this trial. Oversight of protocol adherence, participant safety, and data integrity will be the responsibility of the Principal Investigator and a Monitor appointed by the Research Ethics Committee of Babol University of Medical Sciences. This monitor will continuously review the study progress and submit periodic reports to the Ethics Committee. In the event of any serious adverse event or unforeseen incident, the initial assessment and immediate decision regarding the discontinuation of the intervention for the affected participant or the termination of the entire trial will be made by the Principal Investigator in consultation with the Ethics Committee Monitor. Any major decision (including study termination or significant modifications) will be promptly communicated to the Research Ethics Committee for final deliberation and necessary action. All these processes will be conducted in accordance with national ethical guidelines (the Declaration of Helsinki) and the Research Institute’s internal regulations.

### Patient and public involvement

There was no patient or public involvement in the design, conduct, reporting, or dissemination of this research.

### Patient consent for publication

Consent will be obtained directly from patient(s).

## Discussion

This study protocol has been developed with the aim of designing and conducting a randomized, triple-blind clinical trial to evaluate the efficacy and safety of sumac (Rhus coriaria L.) capsules in reducing menstrual bleeding in women with hypermenorrhea. Hypermenorrhea, as a common condition that significantly affects quality of life, requires safe, accessible, and low-side-effect therapeutic options, particularly in light of the potential adverse effects of conventional hormonal treatments and the increasing patient preference for natural interventions. In this context, sumac, with a long history of use in traditional Iranian medicine and a rich profile of bioactive constituents such as tannins and flavonoids, has gained scientific interest due to its astringent and anti-inflammatory properties [37,38].

Previous meta-analysis evidence suggests sumac reduces hs-CRP [39], and influences oxidative stress markers [38], but direct high-quality clinical evidence for hypermenorrhea is lacking.

### Strengths of this study

This study has several strengths. It employs a randomized, triple-blind, placebo-controlled design which minimizes selection, performance, and detection biases. A validated and reliable tool (PBAC) is used to quantify menstrual blood loss. The herbal formulation is standardized. The sample size is adequate and allocation concealment is ensured. Concomitant medications are pragmatically permitted and will be adjusted for statistically.

### Limitations of this study

This study also has several limitations. The intervention period is relatively short (three menstrual cycles). The trial relies on participant self-reporting (PBAC) rather than objective alkaline hematin measurement. Coagulation parameters are not directly measured. Baseline hemoglobin will not be measured; therefore, mild or asymptomatic anemia may remain undetected. The study is single-center. The use of permitted concomitant medications, including analgesics and iron supplements, may introduce variability in menstrual blood loss across participants. However, their use will be prospectively documented and incorporated into the planned statistical analysis. Finally, the trial does not stratify participants based on the underlying etiology of hypermenorrhea. If sumac proves effective, this trial will provide evidence for an affordable, natural complementary treatment, potentially reducing economic burden. Future studies with longer follow-up and mechanistic investigations are warranted.

## Conclusion

This protocol details a methodologically rigorous trial to generate high-level evidence on the potential of sumac as a complementary treatment for hypermenorrhea. If proven effective, sumac could offer a safe, accessible, and cost-effective therapeutic alternative, aligning with patient preferences for natural interventions. The results will inform future clinical practice and research in integrative women’s health.

## Supporting information

S1 Protocol. English version of the study protocol.
(DOCX)

S2 Protocol. Persian version of the study protocol.
(DOCX)

S3 SPIRIT checklist. SPIRIT checklist for the study protocol.
(DOCX)
