# Supplementary material for: Sumac (Rhus coriaria L.) Capsules for the management of hypermenorrhea: Protocol for a randomized triple-blind trial
Source: PLoS One. 2026 Nov 11;21(11):e0361956. doi: 10.1371/journal.pone.0361956 (PMC13754152; doi:10.1371/journal.pone.0361956)
Supplement: Supplementary file 1 [file pone.0361956.s005.pdf]

PLOS One

Sumac (Rhus coriaria L.) Capsules for the Management of Hypermenorrhea: Protocol for a Randomized Triple-Blind Trial

--Manuscript Draft--

Manuscript Number:	PONE-D-26-06715
Article Type:	Study Protocol
Full Title:	Sumac (Rhus coriaria L.) Capsules for the Management of Hypermenorrhea: Protocol for a Randomized Triple-Blind Trial
Short Title:	Sumac (Rhus coriaria L.) Capsules for the Management of Hypermenorrhea
Corresponding Author:	Fereshteh Behmanesh, PhD<br>Babol University of Medical Science<br>Babol, Mazandaran IRAN, ISLAMIC REPUBLIC OF
Keywords:	Hypermenorrhea; Menorrhagia; Rhus coriaria; Herbal Medicine; Randomized Clinical Trial; Traditional Persian Medicine
Abstract:	Background: Hypermenorrhea is a common gynecological disorder adversely affecting women's quality of life. Conventional treatments, particularly hormonal therapies, are associated with side effects and contraindications. Rhus coriaria L. (sumac) has a historical basis in Persian medicine for its astringent and haemostatic properties, supported by modern phytochemical evidence of tannins and flavonoids. However, robust clinical evidence for its efficacy in hypermenorrhea is lacking. This study aims to evaluate the efficacy and safety of sumac capsules compared to a placebo in reducing menstrual blood loss in women with hypermenorrhea.<br>Methods: A triple-blind, parallel-group, randomized, placebo-controlled clinical trial will be conducted. Sixty eligible women aged 18-45 years with hypermenorrhea (Pictorial Blood Loss Assessment Chart [PBAC] score ≥100) will be randomly allocated to either the intervention group (500 mg sumac capsule twice daily for the first 7 days of menstruation) or the control group (Avicel placebo capsule), over three consecutive menstrual cycles.<br>Discussion: This protocol details a methodologically rigorous trial to generate high-level evidence on the potential of sumac as a complementary treatment for hypermenorrhea. If proven effective, sumac could offer a safe, accessible, and cost-effective therapeutic alternative, aligning with patient preferences for natural interventions. The results will inform future clinical practice and research in integrative women's health.<br>Trial Registration: Iranian Registry of Clinical Trials (IRCT), IRCT20180218038783N12, registered on 5 October 2025.
Order of Authors:	Morteza Mojahedi
	Adeleh Rezaei
	Romina Fili
	Fereshteh Behmanesh, PhD
	Zahra Memariani
	Maryam Nikpour
	Sedigheh Esmaelzadeh
	Sobhan Rahimi Osbo
Additional Information:	
Question	Response
**Financial Disclosure**<br><br>Enter a financial disclosure statement that describes the sources of funding for the	The author(s) received no specific funding for this work.

Powered by Editorial Manager® and ProduXion Manager® from Aries Systems Corporation

work included in this submission. Review the submission guidelines for detailed requirements. View published research articles from *PLOS ONE* for specific examples.<br><br>This statement is required for submission and **will appear in the published article** if the submission is accepted. Please make sure it is accurate.<br><br>**Funded studies**<br>Enter a statement with the following details:<br>• Initials of the authors who received each award<br>• Grant numbers awarded to each author<br>• The full name of each funder<br>• URL of each funder website<br>• Did the sponsors or funders play any role in the study design, data collection and analysis, decision to publish, or preparation of the manuscript?<br><br>Did you receive funding for this work?	
**Competing Interests**<br><br>Use the instructions below to enter a competing interest statement for this submission. On behalf of all authors, disclose any competing interests that could be perceived to bias this work—acknowledging all financial support and any other relevant financial or non-financial competing interests.<br><br>This statement is required for submission and **will appear in the published article** if the submission is accepted. Please make sure it is accurate and that any funding sources listed in your Funding Information later in the submission form are also declared in your Financial Disclosure statement.<br><br>View published research articles from *PLOS ONE* for specific examples.	The authors have declared that no competing interests exist.

**NO authors have competing interests**<br><br>Enter: *The authors have declared that no competing interests exist.*<br><br>**Authors with competing interests**<br><br>Enter competing interest details beginning with this statement:<br><br>*I have read the journal's policy and the authors of this manuscript have the following competing interests: [insert competing interests here]*<br><br>* typeset	
**Ethics Statement**<br><br>Enter an ethics statement for this submission. This statement is required if the study involved:<br><br>• Human participants<br>• Human specimens or tissue<br>• Vertebrate animals or cephalopods<br>• Vertebrate embryos or tissues<br>• Field research<br><br>Write "N/A" if the submission does not require an ethics statement.<br><br>General guidance is provided below. Consult the submission guidelines for detailed instructions. **Make sure that all information entered here is included in the Methods section of the manuscript.**	Ethical approval for this research has been obtained from the Research Ethics Committee of Babol University of Medical Sciences (Approval Code: IR.MUBABOL.REC.1404.106). Prior to their involvement, all potential participants will be comprehensively briefed on the study's aims, methodology, potential risks, and benefits. Enrollment will proceed only after obtaining written informed consent from each individual. To safeguard participant privacy, all collected data will be anonymized using unique codes and stored securely. Participants retain the right to withdraw from the study at any stage without consequence. Findings from this study are intended for dissemination in peer-reviewed scientific publications and conference presentations. We confirm that this manuscript, in whole or in part, is not being considered for publication elsewhere nor has it been previously published.

Powered by Editorial Manager® and ProduXion Manager® from Aries Systems Corporation

**Format for specific study types**<br><br>**Human Subject Research (involving human participants and/or tissue)**<br>• Give the name of the institutional review board or ethics committee that approved the study<br>• Include the approval number and/or a statement indicating approval of this research<br>• Indicate the form of consent obtained (written/oral) or the reason that consent was not obtained (e.g. the data were analyzed anonymously)<br><br>**Animal Research (involving vertebrate animals, embryos or tissues)**<br>• Provide the name of the Institutional Animal Care and Use Committee (IACUC) or other relevant ethics board that reviewed the study protocol, and indicate whether they approved this research or granted a formal waiver of ethical approval<br>• Include an approval number if one was obtained<br>• If the study involved *non-human primates*, add *additional details* about animal welfare and steps taken to ameliorate suffering<br>• If anesthesia, euthanasia, or any kind of animal sacrifice is part of the study, include briefly which substances and/or methods were applied<br><br>**Field Research**<br><br>Include the following details if this study involves the collection of plant, animal, or other materials from a natural setting:<br>• Field permit number<br>• Name of the institution or relevant body that granted permission	
**Data Availability**<br><br>Authors are required to make the data underlying their research findings fully available, without restriction.<br><br>If the manuscript reports pilot or preliminary results, the data underlying those results must be made available at the time of publication. A Data Availability	N/A - No results are reported

Statement describing where the data can be found is required at submission. Your answers to this question constitute the Data Availability Statement and will be published in the article, if accepted. When legal or ethical requirements prohibit public data sharing, authors must indicate how researchers may obtain access to the data. See the PLOS Data Policy and FAQ for detailed information.<br><br>**Important:** Stating 'data available on request from the author' is not sufficient. If your data are only available upon request, select 'No' for the first question and explain your situation in the text box.<br><br>For protocols without pilot or preliminary data, authors are strongly encouraged to state how they plan to share research data from their study when it is completed or published.<br><br>If your study includes pilot data, will they be made fully available without restriction?	
**Describe where the data may be found in full sentences. If you are copying our sample text, replace any instances of XXX with the appropriate details.**<br><br>• If **no pilot data are reported** in the manuscript, please enter the following: *No datasets were generated or analysed during the current study. All relevant data from this study will be made available upon study completion.*<br>• If the manuscript reports pilot data **held in a public repository**, include URLs, accession numbers or DOIs. If this information will only be available after acceptance, indicate this by ticking the box below. For example: *All XXX files are available from the XXX database (accession number(s) XXX, XXX.).*<br>• If the manuscript reports pilot data contained **within the manuscript and/or**	N/A

**Supporting Information files**, enter the following:<br>*All relevant data are within the manuscript and its Supporting Information files.*<br>• If neither of these applies but you are able to provide **details of access elsewhere**, with or without limitations, please do so. For example:<br><br>*Data cannot be shared publicly because of [XXX]. Data are available from the XXX Institutional Data Access / Ethics Committee (contact via XXX) for researchers who meet the criteria for access to confidential data.*<br><br>*The data underlying the results presented in the study are available from (include the name of the third party and contact information or URL).*<br>• This text is appropriate if the data are owned by a third party and authors do not have permission to share the data.<br><br>For study protocols with data management or sharing plans, authors are encouraged to briefly describe in their Data Availability Statement how data they generate will be made accessible when the study is completed. For example:<br>• Deidentified research data will be made publicly available when the study is completed and published.<br><br>* typeset	
Additional data availability information:	

Powered by Editorial Manager® and ProduXion Manager® from Aries Systems Corporation

Manuscript
Click here to access/download;Manuscript;Sumac protocole manuscript 21-11-404.docx

Sumac (Rhus coriaria L.) Capsules for the Management of Hypermenorrhea: Protocol for a Randomized Triple-Blind Trial

Morteza Mojahedi[1], Adeleh Rezaei[2], Romina Fili[3], Fereshteh Behmanesh[4*], Zahra Memariani[5], Maryam Nikpour[6], Seddigheh Esmaeilzadeh[7], Sobhan Rahimi Osbo[8]

[1] Traditional Medicine and History of Medical Sciences Research Center, Health Research Institute, Babol University of Medical Sciences, Babol, Iran
mortazamojahedy@gmail.com ORCID: 0000-0002-0346-5925

[2] Student Research Committee, Babol University of Medical Sciences, Babol, I.R. Iran adele.rezaei82@gmail.com

[3]Student Research Committee, Babol University of Medical Sciences, Babol, I.R. Iran rominafili.mid@gmail.com ORCID: 0009-0005-0705-8007

[4]* (Correspondence) Infertility and Reproductive Health Research Center, Health Research Institute, Babol University of Medical Sciences, Babol, I.R. Iran f.behmanesh@mubabol.ac.ir f.behmanesh2015@gmail.com http://orcid.org/0000-0001-5837-4265 Tel:09832199594

[5] Traditional Medicine and History of Medical Sciences Research Center, Health Research Institute, Babol University of Medical Sciences, Babol, I.R.Iran z.memariani@mubabol.ac.ir ORCID:0000-0002-2052-4261

[6] Non-Communicable Pediatric Disease Research Center, Health Research Institute, Babol University of Medical Sciences, Babol, I.R.Iran Email: maryamnikpour19@yahoo.com ORCID: 0000-0001-6821-0010

[7] Infertility and Reproductive Health Research Center, Health Research Institute, Babol University of Medical Sciences, Babol, I.R. Iran sesmael2010@gmail.com http://orcid.org/0000-0002-1951-1367

[8] Social Determinants of Health Research Center, Health Research Institute, Babol University of Medical Sciences, Babol, I.R. Iran Email: rrrrsobhan@gmail.com

Sumac (Rhus coriaria L.) Capsules for the Management of Hypermenorrhea: Protocol for a Randomized Triple-Blind Trial

Abstract

Background: Hypermenorrhea is a common gynecological disorder adversely affecting women's quality of life. Conventional treatments, particularly hormonal therapies, are associated with side effects and contraindications. *Rhus coriaria L.* (sumac) has a historical basis in Persian medicine for its astringent and haemostatic properties, supported by modern phytochemical evidence of tannins and flavonoids. However, robust clinical evidence for its efficacy in hypermenorrhea is lacking. This study aims to evaluate the efficacy and safety of sumac capsules compared to a placebo in reducing menstrual blood loss in women with hypermenorrhea.

Methods: A triple-blind, parallel-group, randomized, placebo-controlled clinical trial will be conducted. Sixty eligible women aged 18-45 years with hypermenorrhea (Pictorial Blood Loss Assessment Chart [PBAC] score ≥100) will be randomly allocated to either the intervention group (500 mg sumac capsule twice daily for the first 7 days of menstruation) or the control group (Avicel placebo capsule), over three consecutive menstrual cycles.

Discussion: This protocol details a methodologically rigorous trial to generate high-level evidence on the potential of sumac as a complementary treatment for hypermenorrhea. If proven effective, sumac could offer a safe, accessible, and cost-effective therapeutic alternative, aligning with patient preferences for natural interventions. The results will inform future clinical practice and research in integrative women's health.

Trial Registration: Iranian Registry of Clinical Trials (IRCT), IRCT20180218038783N12, registered on 5 October 2025.

Keywords: Hypermenorrhea; Menorrhagia; Rhus coriaria; Herbal Medicine; Randomized Clinical Trial; Traditional Persian Medicine.

Highlights

- This study employs a randomized, placebo-controlled, triple-blind design, which minimizes selection, performance, and detection biases and strengthens the internal validity of the findings.
- Use of a standardized and validated outcome measure (Pictorial Blood Loss Assessment Chart, PBAC) enables objective assessment of menstrual blood loss and enhances comparability with previous studies.
- The intervention is based on both traditional Persian medicine and contemporary phytochemical evidence, providing a strong translational rationale for evaluating sumac as a complementary therapy for hypermenorrhea.

Introduction

Abnormal menstrual bleeding is a medical condition characterized by irregular menstrual cycles, including excessive menstrual blood loss. Menstrual bleeding exceeding 80 milliliters per cycle or lasting longer than seven days is considered abnormal, a condition referred to as hypermenorrhea [1, 2]. The prevalence of abnormal menstrual bleeding worldwide varies and depends on multiple factors, including geographical region, cultural context, socioeconomic status, access to and effectiveness of medical care, and environmental factors [1, 3].
Complications of abnormal menstrual bleeding include iron deficiency, physical symptoms such as pain and fatigue, and, consequently, a reduced quality of personal and social life among women of reproductive age [4].
The most common causes of abnormal menstrual bleeding include pregnancy-related conditions, structural uterine pathologies (such as fibroids, endometrial polyps, and adenomyosis), ovulatory disorders, and neoplastic disorders, which, through excessive blood loss, can lead to anemia, coagulopathy, acidosis, hypothermia, and coagulation abnormalities [4, 5]. Hormonal disorders and platelet-related blood abnormalities, such as vascular diseases and coagulation disorders, can also influence this condition [6, 7].

Based on these varying factors, different treatment approaches are considered for hypermenorrhea. The first-line treatment for hypermenorrhea is pharmacological therapy.
Pharmacological treatments include the use of nonsteroidal anti-inflammatory drugs, hormone therapy, progestin-containing medications, and progesterone-releasing intrauterine devices. In cases of inadequate response to medical therapy, surgical approaches such as hysterectomy or endometrial ablation are employed [8]. In some cases, emergency treatment is required, such as in the presence of severe bleeding, which may result from acute blood loss (hemorrhage) [9, 10].
In traditional Iranian medicine texts, excessive menstrual flow, prolonged menstrual duration, and bleeding outside the menstrual period are referred to as efrāṭ-e ṭamth or kasrat-e ṭamth [11].The general principles of treatment in traditional Iranian medicine are based on lifestyle modification, with nutrition considered a key component. If no improvement is observed, the next steps recommended are pharmacological treatment followed by manual or procedural interventions [12]. The efficacy of certain pharmacological treatments has been investigated

in clinical trials, which have demonstrated positive effects [13]. In the first step, hematinic treatments consisting of Khabth al-Hadid derivatives are prescribed to the patient. In the second step, herbal astringents such as quince paste, myrtle, pomegranate flower, and plantain are administered, depending on availability and cost-effectiveness [11, 14].

Another substance mentioned in traditional Iranian medicine for its effectiveness in controlling and treating abnormal uterine bleeding is sumac. Reduced blood viscosity due to increased heat and fluidity can be one of the causes of bleeding. During menstruation, the released blood is described as thin, yellowish, warm, and burning, and it exits under pressure. An appropriate intervention in this condition is to lower the "heat" of the blood. Therefore, foods considered "cooling" in traditional Iranian medicine are used—these include pomegranate, barberry, unripe grape, rhubarb, sumac, and vinegar. Causes of uterine bleeding include lesions such as ulcers, masses, trauma, difficult childbirth, or any factor that damages the uterus. In these cases, alongside treating the underlying cause, the consumption of astringent foods is recommended. These substances, which have a constrictive effect, help contract the uterus, close the vessels, and stop bleeding. Such foods include quince, apple, lentils, sumac, sour grapes, pomegranate, barberry, oak, Russian olives, and vinegar. [15].

Sumac is a plant from the pistachio family, with the scientific name Rhus coriaria L [16, 17]. The flowers of the sumac shrub are dense, clustered, and honey-colored. Sumac primarily grows in rocky areas and on hard, rugged soils [18].

The nature of sumac is cold and dry in the second degree, and it possesses actions and properties such as astringent, restraining, stomach-tanning, strengthening the warm organs, and reducing bile. It has traditionally been used to treat nausea and vomiting caused by bile, bleeding, and chronic diarrhea [19].

In traditional Iranian medicine, sumac has been regarded as a preventive agent against cardiovascular diseases and is consumed alongside certain foods [20]. Many recent findings regarding the effects of sumac on digestive disorders, diarrhea, as well as its antiseptic and analgesic properties, are consistent with the observations of traditional Iranian medicine scholars [21]. Sumac has also long been used as a food seasoning and for strengthening the stomach and gums, reducing or stopping bleeding, and preventing infectious diseases [22].

One of the therapeutic uses of sumac in traditional Iranian medicine is its application in the preparation of sumac porridge (Somāqīyeh), which has been used to treat conditions such as indigestion and diarrhea [23], nausea, nosebleeds, blood-related joint pain, hiccups, and bleeding from hemorrhoidal vessels [24]. According to written Iranian medical sources, this plant has also been used for uterine disorders [25, 26]. Studies have shown that sumac increases platelet distribution width [27]. It can also influence bleeding time, prothrombin time, and activated partial thromboplastin time, suggesting its potential as an adjuvant therapy for bleeding control. In a study by Salsali and colleagues titled Dietary Recommendations for Abnormal Uterine Bleeding in Traditional Iranian Medicine, which reviewed classical Iranian medical texts such as Qanun fi al-Tibb, Tibb Akbari Arzani, Maʿalijat Aghili Khorasani, Sharh al-Asbab Samarkandi, and Aksir-e Azam Nazem Jahan, it was reported that the consumption of astringent foods can be effective in reducing menstrual bleeding. One such food is sumac [15] .

The active compound in sumac is tannins [28] .Sumac is also rich in magnesium, calcium, tannins, flavonoids, and other polyphenolic compounds. Its astringent properties are

attributed to malic acid, tannic acid, gallic acid, and tannins. Tannins and flavonoids stimulate estrogen receptors, the smooth muscles of the myometrium, and uterine vascular contraction [14]. Experimental and preclinical studies suggest that polyphenols and tannins present in Rhus coriaria may influence inflammatory and vascular pathways. However, the relevance of these mechanisms to uterine bleeding in humans remains uncertain and requires confirmation through well-designed clinical trials [29].

A study titled Anti-inflammatory Properties of Sumac (Rhus coriaria L.) Powder Combined with Iron Sulfate on Hematological Indices and Oxidative Stress in Young Female Athletes with Non-anemic Iron Deficiency reported that sumac powder did not enhance the effect of iron supplementation and even had a negative impact [30]. Momenimovahed and Khalajinia (2019) demonstrated that ethanolic extract of sumac fruit significantly reduced the expression of inflammatory genes IL-1β and IL-18 in LPS-stimulated synovial cells, showing notable anti-inflammatory effects [31]. Since these cytokines play a role in many inflammatory processes, including endometrial inflammation and abnormal uterine bleeding, it can be inferred that sumac may indirectly reduce uterine bleeding by inhibiting inflammatory responses. However, to confirm this effect definitively, direct clinical studies on uterine health are necessary. Additionally, a systematic review of five studies investigating sumac's effects on oxidative stress found that four studies reported significant reductions in malondialdehyde (MDA) levels and increases in total antioxidant capacity (TAC) and paraoxonase-1 (PON1). Regarding inflammation, one study reported no significant differences in TNF-α or IL-6 levels between intervention and control groups [32].
Given references in traditional medicine texts to the potential pro-coagulant effects of *Rhus coriaria* and the limited experimental studies suggesting hemostatic activity, well-designed human trials are required to clarify its clinical effects on menstrual bleeding. Moreover, hormonal therapies commonly used for heavy menstrual bleeding are associated with adverse effects and contraindications in certain populations, including increased thromboembolic risk and limitations in women with cardiovascular, metabolic, or hepatic conditions. Alongside growing interest in complementary therapies due to their accessibility, lower cost, and perceived safety, preliminary evidence from Iranian medical sources supports a potential role for sumac in the management of uterine bleeding. Therefore, this randomized, triple-blind, placebo-controlled trial is designed to evaluate the efficacy and safety of sumac capsules in women with moderate to heavy menstrual bleeding, with the aim of generating preliminary clinical evidence to inform future confirmatory studies.

Objectives

The primary objective of this trial is to evaluate whether oral administration of *Rhus coriaria* capsules reduces menstrual blood loss, measured by changes in PBAC scores, compared with placebo over three consecutive menstrual cycles.

Secondary objectives include assessing the safety and tolerability of sumac capsules, evaluating changes in bleeding duration, and recording the frequency and type of adverse events during the intervention period.

Methods

Study Design and Setting

This study will be conducted as a triple-blind, parallel-group randomized clinical trial with random allocation of participants into two groups: intervention and control. This manuscript follows the SPIRIT 2013 guidelines (Supplementary file 1). The trial was registered in the Iranian Clinical Trial Registry (Code: IRCT20180218038783N12) on 5 October 2025. The recruitment period for this study started on 22 October 2025 and is expected to continue until October 2027. Participants will be recruited from a traditional medicine clinic, a gynecology clinic, and the female dormitory of Babol University of Medical Sciences. Time schedule of enrolment, interventions, assessments, and visits for participants is shown in Fig 1. The study design also follows the consolidated standards of CONSORT clinical trial reports (Fig 2).

Eligibility Criteria

Eligible participants will include women aged 18–45 years with menstrual blood loss >80 mL per cycle, assessed using the Pictorial Blood Loss Assessment Chart (PBAC) (score ≥100), consent to participate in study, a regular menstrual cycle of 21–42 days, and literacy.

Exclusion criteria will include gynecological pathologies (ovarian cysts, endometrial polyps, neoplasms), irregular menstrual cycles, chronic medical conditions (hypertension, uncontrolled diabetes, renal, hepatic, cardiovascular, or thyroid disorders), use of anticoagulants or hormonal contraceptives, smoking or alcohol consumption, abnormal endometrial thickness on ultrasonography, first-degree family history of cancer, uterine fibroids >3 cm (submucosal or intramural), known allergy to sumac, adherence to a special diet in the previous three months, and severe bleeding requiring hospitalization.

Withdrawal criteria will include <80% treatment adherence, pregnancy or breastfeeding, and unwillingness to continue participation.

Randomization, Allocation Concealment, and Blinding

Participants will be enrolled using convenience sampling and randomly assigned in a 1:1 ratio to the intervention or placebo group using block randomization (block size = 10) generated by Random Allocation software. Allocation concealment will be ensured using sequentially numbered, opaque, sealed envelopes prepared by an independent individual. Random sequence generation, participant enrollment, and group assignment will be performed by separate personnel. The researchers, participants, and data analyst will remain blinded to group allocation throughout the study.

To ensure allocation concealment and preserve the integrity of randomization, sealed opaque envelopes containing the allocation assignments will be used. Based on the predetermined sample size, 60 sequentially numbered envelopes will be prepared, each enclosing a card

indicating the random allocation. At the time of participant enrollment, envelopes will be opened in sequence according to the order of recruitment to determine group assignment.

Implementation

After selecting participants based on the inclusion criteria and obtaining informed consent, they will be randomly allocated into two groups. The intervention group will receive capsules containing an aqueous extract of sumac fruit (A++ grade), obtained from Giah Kala Company, with the total flavonoid content determined. Each capsule will be equivalent to 500 mg of dried fruit. The control group will receive placebo capsules containing 500 mg of Avicel, purchased from Behboud Shimi Company (Gloria brand). All capsules will be prepared and coded by a pharmacist specialized in traditional medicine who is not involved in participant recruitment. Participants will take two capsules daily, at 12-hour intervals, during the first seven days of menstruation, for three consecutive menstrual cycles. If required, mefenamic acid and iron supplements will be prescribed to both groups and recorded.

Primary and Secondary Outcome

The primary outcomes of this study are the intensity and duration of menstrual bleeding, assessed using the Pictorial Blood Loss Assessment Chart (PBAC). Bleeding intensity will be evaluated based on the total PBAC score, while bleeding duration will be determined by the number of bleeding days recorded in the PBAC chart. Outcomes will be assessed at baseline and after one, two, and three menstrual cycles following initiation of the intervention.

Secondary outcomes include the occurrence, type, and severity of any adverse effects associated with sumac capsule consumption, which will be recorded prospectively throughout the study period.

Assessment and Recording of Adverse Events

Adverse events will be assessed at each monthly follow-up visit through structured interviews and participant self-reporting. Participants will be instructed to report any gastrointestinal symptoms, allergic reactions, dizziness, nausea, or other unexpected symptoms during the intervention period.

Adverse events will be categorized according to severity (mild, moderate, severe) and their potential relationship to the intervention (unlikely, possible, probable). Serious adverse events will be reported immediately to the ethics committee.

Instruments

Menstrual blood loss will be assessed using the PBAC (Higham Chart), a validated and reliable tool, completed at baseline and during each menstrual cycle. The Pictorial Blood Loss Assessment Chart (PBAC) was originally developed by Higham, O'Brien, and Shaw in 1990 as a semi-quantitative tool for assessing menstrual blood loss [33]. This chart is a

simple, non-laboratory visual assessment tool for the evaluation of menstrual bleeding. The scoring system is based on the visual inspection of sanitary pads, tampons, and the presence of blood clots. The chart has a two-dimensional structure, with the number of menstrual days represented on the horizontal axis and the degree of blood-soaked pads on the vertical axis. Light staining (spotting or minimally soiled pads) was assigned a score of 1, moderate staining a score of 5, and heavy saturation a score of 20. In addition, the passage of small clots (approximately the size of a small coin) was scored as 1, large clots (approximately the size of a large coin) as 5, and each episode of flooding (soiling of underwear or free flow of blood into the toilet) was scored as 5. At the end of menstruation, the frequency of each item was multiplied by its corresponding score, and the total PBAC score was calculated by summing all values. A PBAC score of 100 or higher corresponds to a menstrual blood loss exceeding 80 mL. The Higham chart is a validated instrument for quantifying menstrual blood loss and The Higham chart is a valid instrument, and its reliability was reported as r = 0.88 in the study by Mohamadirizi et al in Iran [34].

Demographic, socioeconomic, and obstetric data will be collected at baseline using a structured questionnaire including age, body mass index (BMI), educational level, place of residence, employment status, and satisfaction with household income, as well as obstetric information such as the number of pregnancies, deliveries, and abortions, and the method of contraception, were collected. Data related to abnormal uterine bleeding included menstrual cycle characteristics, namely cycle regularity, intermenstrual interval, duration of bleeding, presence of dysmenorrhea, and volume of menstrual blood loss.

Sample Size

Based on expert clinical opinion, a minimum clinically significant difference of 10 units in PBAC scores between groups will be assumed. With a standard deviation of 6.5, an effect size of 0.80, a 95% confidence level, and 90% power, the required sample size will be 27 participants per group. Allowing for a 15% attrition rate, 30 participants per group will be recruited.

Statistical Analysis

All analyses will be done according to the intention-to-treat principle. Continuous variables will be summarized as means ± standard deviations or medians with interquartile ranges, as appropriate. Changes in PBAC scores over time will be analyzed using repeated-measures analysis of variance or linear mixed-effects models, with group, time, and group-by-time interaction terms included. Between-group differences at each time point will be assessed with independent t-tests or non-parametric equivalents. Missing data will be handled using multiple imputation methods if the proportion of missing data exceeds 5%; otherwise, last observation carried forward will be applied. A two-sided p-value <0.05 will be considered statistically significant.

Ethics and dissemination

Ethical approval for this study was obtained from the Research Ethics Committee of Babol University of Medical Sciences (Approval Code: IR.MUBABOL.REC.1404.106) on 4 August 2025. Prior to their involvement, all potential participants will be comprehensively briefed on the study's aims, methodology, potential risks, and benefits. Enrollment will proceed only after obtaining written informed consent from each individual. To safeguard participant privacy, all collected data will be anonymized using unique codes and stored securely. Participants retain the right to withdraw from the study at any stage without consequence. Findings from this study are intended for dissemination in peer-reviewed scientific publications and conference presentations. We confirm that this manuscript, in whole or in part, is not being considered for publication elsewhere nor has it been previously published.

Study Oversight and Safety

Given the nature of the intervention (sumac capsule as a herbal product with a known safety profile), the limited sample size, and the short intervention period, the establishment of an independent Data Monitoring Committee (DMC) will not be required for this trial. Oversight of protocol adherence, participant safety, and data integrity will be the responsibility of the Principal Investigator and a Monitor appointed by the Research Ethics Committee of Babol University of Medical Sciences. This monitor will continuously review the study progress and submit periodic reports to the Ethics Committee. In the event of any serious adverse event or unforeseen incident, the initial assessment and immediate decision regarding the discontinuation of the intervention for the affected participant or the termination of the entire trial will be made by the Principal Investigator in consultation with the Ethics Committee Monitor. Any major decision (including study termination or significant modifications) will be promptly communicated to the Research Ethics Committee for final deliberation and necessary action. All these processes will be conducted in accordance with national ethical guidelines (the Declaration of Helsinki) and the Research Institute's internal regulations.

Discussion

This study protocol has been developed with the aim of designing and conducting a randomized, double-blind clinical trial to evaluate the efficacy and safety of sumac (Rhus coriaria L.) capsules in reducing menstrual bleeding in women with hypermenorrhea. Hypermenorrhea, as a common condition that significantly affects quality of life, requires safe, accessible, and low-side-effect therapeutic options, particularly in light of the potential adverse effects of conventional hormonal treatments and the increasing patient preference for natural interventions. In this context, sumac, with a long history of use in traditional Iranian medicine and a rich profile of bioactive constituents such as tannins and flavonoids, has gained scientific interest due to its astringent and anti-inflammatory properties [35, 36].

Previous studies support the potential of this plant to modulate coagulation parameters and inhibit inflammatory biomarkers; however, direct and high-quality clinical evidence regarding its effects on hypermenorrhea remains limited. A systematic review and meta-analysis of randomized trials indicates that Rhus coriaria (sumac) supplementation may significantly reduce high-sensitivity C-reactive protein (hs-CRP), an established inflammatory biomarker, in adults, suggesting anti-inflammatory activity in clinical settings

[37]. A randomized clinical trial has demonstrated that sumac intake can influence oxidative stress and inflammatory markers such as malondialdehyde (MDA) and protein and paraoxonase 1 activity, further supporting its biological activity toward pathways linked to inflammation and coagulation [38]. However, direct and high-quality clinical evidence regarding its effect on hypermenorrhea remains limited [37]. By employing a randomized, placebo-controlled design, utilizing the standardized Pictorial Blood Assessment Chart (PBAC) to quantify menstrual blood loss, and ensuring standardization of the herbal formulation, the present trial aims to address the existing gap in the scientific literature.

If the results of this study demonstrate a significant therapeutic effect of sumac, it may represent an important step toward the introduction of an evidence-based complementary treatment option. Such findings would not only align with patients' growing preference for natural therapies but may also contribute to reducing the economic burden of treatment. Nevertheless, attention to potential limitations, including the relatively short intervention period and reliance on patient self-reporting, is warranted. Future studies with longer follow-up durations and investigations into the precise molecular mechanisms underlying the effects of sumac are therefore recommended.

Overall, this protocol provides a structured, evidence-based framework to test a traditional medicine-derived hypothesis within the context of rigorous clinical research, potentially informing clinical decision-making and guiding larger integrative medicine trials.

Conclusion

This protocol details a methodologically rigorous trial to generate high-level evidence on the potential of sumac as a complementary treatment for hypermenorrhea. If proven effective, sumac could offer a safe, accessible, and cost-effective therapeutic alternative, aligning with patient preferences for natural interventions. The results will inform future clinical practice and research in integrative women's health.

Author Contributions

F.B. & M.M.: Conceptualization, Methodology, Writing- Original draft preparation Z.M., S.E., M.N., & S.R Methodology, A.R. & R.F. Data curation, All authors. Approve final manuscript. Authorship eligibility for the final manuscript will be determined according to the International Committee of Medical Journal Editors (ICMJE) guidelines, which require substantial contributions to the study's conception, design, execution, analysis, or drafting. The use of paid professional writers is not anticipated for this research.

Acknowledgements

The authors would like to thank the Deputy of Research and Technology of Babol University of Medical Sciences and mothers who will participate in this study. (Study code: 724136410).

Data Availability

Not Applicable. This is study protocol.

Funding statement

The authors received no specific funding for this study.

Competing interest's statement

The authors have declared that no competing interests exist.

Patient and public involvement

This research did not involve patients or the public in its design, conduct, reporting, or dissemination activities.

Patient consent for publication

Consent will be obtained directly from patient(s).

References

.1 Borzutzky, C. and J. Jaffray, *Diagnosis and management of heavy menstrual bleeding and bleeding disorders in adolescents.* JAMA pediatrics, 2020. **174**(2): p. 186-194.

.2 De Sanctis, V., et al., *Hypomenorrhea in adolescents and youths: normal variant or menstrual disorder? Revision of literature and personal experience.* Acta Bio Medica: Atenei Parmensis, 2022. **93**(1): p. e2022157.

.3 Ibrahim, P.M. and E.L. Samwel, *Prevalence of Heavy Menstrual Bleeding and Its Associated Factors Among Women Attending Kilimanjaro Christian Medical Centre In Northern Eastern, Tanzania: A Cross-Sectional Study.* The East African Health Research Journal, 2023. **7**(1): p. 1.

.4 Kaunitz, A.M., et al., *Approach to abnormal uterine bleeding in nonpregnant reproductive-age women.* Internet) Walthman (MA): UpToDate, 2015.

.5 Ash, T., *Massive transfusion protocol.* Archives of Hematology Mini Reviews and Reviews, 2023. **8**(1): p. 004-007.

.6 Kulkarni, R., *Improving care and treatment options for women and girls with bleeding disorders.* European journal of haematology, 2015. **95**: p. 2-10.

.7 Bradley, L.D. and N.-A. Gueye, *The medical management of abnormal uterine bleeding in reproductive-aged women.* American journal of Obstetrics and Gynecology, 2016. **214**(1): p. 31-44.

.8 Rodriguez, M.B., A .Lethaby, and V. Jordan, *Progestogen-releasing intrauterine systems for heavy menstrual bleeding.* Cochrane Database of Systematic Reviews, 2020.(6)

.9 Isono, W., et al., *Unilateral uterine artery embolization and Bakri tamponade balloon insertion in the treatment of acute puerperal uterine inversion: a case report.* Journal of Medical Case Reports, 2022. **16**(1): p. 190.

.10 Kaganov, H. and A. Ades, *Uterine fibroids: investigation and current management trends.* Australian Family Physician, 2016. **45**(10): p. 7.22-725

.11 Tansaz, M., et al., *Menorrhagia management in Iranian traditional medicine.* Journal of evidence-based complementary & alternative medicine, 2016. **21**(1): p. 71-76.

.12 Falahat, F., et al., *Natural treatments of oligomenorrhea based on persian medicine.* The Iranian Journal of Obstetrics, Gynecology and Infertility, 2018. **21**(Supplement): p. 55-66.

.13 Fesharaki, G. and N. Zafarghandi, *Comparison of effects of pomegranate peel and Mefenamic acid consumption on the treatment of Menorrhagia: A Triple-Blind Randomized Controlled Trial.* 2019.

.14 Ashteany, E.-h., et al., *Effectiveness of Persian Golnar on Excessive Menstrual Bleeding in Women with Abnormal Uterine Bleeding, Compared to Tranexamic Acid: A Triple-Blind, Randomized Equivalence Trial.* Evidence-Based Complementary and Alternative Medicine, 2023. **2023**.

.15 Salsali, G., *Food recommendations for abnormal uterine bleeding in Traditional Iranian medicine on the basis of Hemorheology.* Int J Ayurved Med, 2019. **10**(1): p. 1-6.

.16 Ghahreman, A. and A. Okhovvat, *Matching the old medicinal plant names with scientific terminology. vol. 1. Farsi ed*. 2004, Tehran University Press, Iran.

.17 Mozaffarian, V., *A dictionary of Iranian plant names: Latin, English, Persian*. 1996: Farhang Mo'aser.

.18 Ahmad, H ,.et al., *Unani description of Sumaq (Rhus coriaria Linn.) and its scientific report.* Global Journal of Medical Research, 2013. **13**(7): p. 75-78.

.19 Khoshkharam, M., et al., *Sumac: A functional food and herbal remedy in traditional herbal medicine in the Asia*, in *Functional foods and nutraceuticals in metabolic and non-communicable diseases*. 2022, Elsevier. p. 261-266.

.20 Zargham, H. and R. Zargham, *Tannin extracted from Sumac inhibits vascular smooth muscle cell migration.* McGill Journal of Medicine: MJM, 2008. **11**(2): p. 119.

.21 Mohammadi, S., et al., *Effect of hydroalcoholic leaves extract of Rhus Coriaria on pain in male rats.* Anesthesiology and pain medicine, 2016. **6**.(1)

.22 Mosaffa-Jahromi, M. and H. Kiani, *Alshamel fi Sana'at Tebbi'at: A Comprehensive Book on the Materia Medica.* Iranian Journal of Medical Sciences, 2016. **41**(3 Suppl): p. S20.

.23 Razi, B., *Kholasat ol-Tajarob.* The Institute for Medical History-Islamic and Complementary Medicine, Tehran University of Medical Sciences, Tehran, 2000 :p. 134-153.

.24 MA, A., *Tibb-i Akbari (Akbari's Medicine).* Qom: Jalal al-Din Publications, 2008. **2**: p. 690-695.

.25 Ibn-e-Sina, A., *Al-qanun fit-tib [The canon of medicine]*. 2005, Beirut, Lebanon: Alaalami Beirut lib Press.

.26 Aghili Shirazi, S., *Makhzan Al-adviyah (the Storehouse of medicaments).* Tehran: Tehran University of Medical Sciences, 2009.

.27 Capcarova, M., et al., *Effects of dietary inclusion of Rhus coriaria on internal milieu of rabbits.* Journal of animal physiology and animal nutrition, 2 :(3)96 .012p. 459-465.

.28 Khodabakhsh, M., et al., *The effect of plantain syrup on heavy menstrual bleeding: A randomized triple blind clinical trial.* Phytotherapy Research, 2020. **34**(1): p. 118-125.

.29 Payranj, M., et al., *Investigating the Impact of Sumac Capsules on Postpartum Bleeding among Women at Risk of Bleeding: a three-blind randomized clinical trial.* Journal of Pharmacopuncture, 2024. **27**(4): p. 287.

.30 Amirsasan, R., et al., *The effect of anti-inflammatory properties of sumac powder (Rhus coriaria L.) along with ferrous sulfate on blood markers and oxidative stress of young athletic girls with non-anemic iron deficiency (NAID).* Sport Sciences for Health, 2024: p. 1-9.

.31 Momenimovahed, Z. and Z. Khalajinia, *Unwillingness to have Vaginal Delivery among Iranian Women: A Qualitative Study.* Current Womens Health Reviews, 2025. **21**(1): p. E240124226088.

.32 Bahari, H., et al., *The Effects of Sumac Consumption on Inflammatory and Oxidative Stress Factors: A Systematic Review of Randomized Clinical Trials.* Current Pharmaceutical Design, 2024. **30**(27): p. 2142-2151.

.33 Higham, J.M., P. O'brien, and R. Shaw, *Assessment of menstrual blood loss using a pictorial chart.* BJOG: An International Journal of Obstetrics & Gynaecology, 1990. **97**(8): p. 734-739.

.34 Mohamadirizi, S., et al., *Relationship between Job Stress with Menstrual Bleeding Pattern among Midwives.* Hayat/Ḥayāt, 2012. **18**.(5)

.35 Elagbar, Z.A., et al., *Phytochemical Diversity and Pharmacological Properties of Rhus coriaria.* Chem Biodivers, 20 :(4)17 .20p. e1900561.

.36 Alsamri, H., et al., *Pharmacological and Antioxidant Activities of Rhus coriaria L. (Sumac).* Antioxidants (Basel), 2021. **10**.(1)

.37 Jazinaki, M.S., et al., *Sumac (Rhus coriaria L.) Supplementation on High-Sensitivity C-Reactive Protein Concentrations in Adults: A Systematic Review and Dose-Response Meta-Analysis.* Curr Dev Nutr, 2025. **9**(10): p. 107555.

.38 Rahideh, S.T., et al., *The effect of sumac (Rhus coriaria L.) powder on insulin resistance, malondialdehyde, high sensitive C-reactive protein and paraoxonase 1 activity in type 2 diabetic patients.* J Res Med Sci, 2014. **19**(10): p. 933-8.

Fig 1

Click here to access/download;Figure;Fig 1. SPIRIT schedule.tif.tif

Fig 1. SPIRIT schedule of enrolment, interventions, and assessments.

	STUDY PERIOD						
	Enrolment	Allocation	Post-allocation				Close-out
TIMEPOINT	$-t_1$	0	T_0	T_1 During first cycle	T_2 During second cycle	T_3 During third cycle	October 2027
ENROLMENT:							
Eligibility screen	X						
Informed consent	X						
Allocation		X					
INTERVENTIONS:							
capsules of Sumac				⟷	⟷	⟷	⟷
& Placebo							
ASSESSMENTS: Pictorial Blood Loss Assessment Chart (PBAC)			X	X	X	X	X

The figure was prepared in accordance with the SPIRIT 2013 Statement and outlines the timing of enrolment, allocation, interventions, and outcome assessments for the trial.

Fig 2

Click here to access/download;Figure;Fig 2. tif.tif

Fig 2: Flow of participants through the study

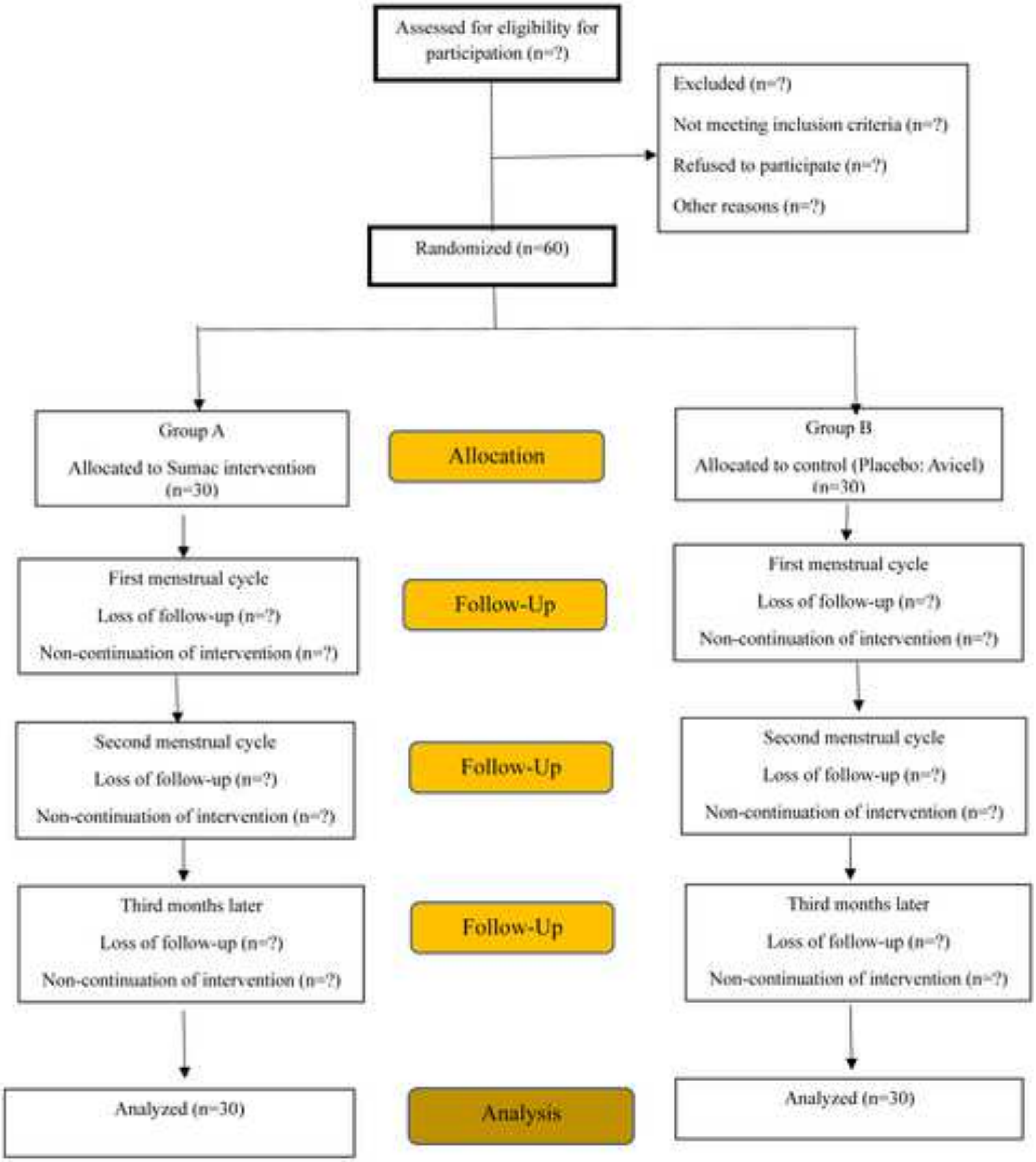

SPIRIT checklist

Click here to access/download
Supporting Information
Sumac SPIRIT.docx

Click here to access/download
Supporting Information
Sumac persian -15-9-404.docx

Translated protocole

Click here to access/download
Supporting Information
Sumac English Protocol.docx

Dear PLOS ONE Editorial Office,

Thank you for your careful technical check of our manuscript entitled "**Sumac (Rhus coriaria L.) Capsules for the Management of Hypermenorrhea: Protocol for a Randomized Triple-Blind Trial**" (Manuscript ID: PONE-D-26-06715).

We have addressed all requested points as detailed below:

1. **SPIRIT Schedule (Figure):** The SPIRIT schedule has been re-uploaded as **Fig 1**, in accordance with PLOS ONE submission guidelines. Figure numbering, legends, and in-text citations have been amended accordingly.
2. **SPIRIT Checklist:** A completed **SPIRIT checklist** has now been uploaded as **Supporting Information**.
3. **Ethics-Approved Study Protocol:** We would like to clarify the language of the submitted study protocol in response to your request. The study protocol was originally submitted to and approved by the institutional ethics committee in **Persian**, which is therefore the official and authoritative version. In accordance with the journal's requirements for Clinical Trial Study Protocols, we have now provided:

- The **original Persian ethics committee–approved protocol**, and
- A **complete English translation** of this protocol for editorial and reviewer assessment.

Both documents have been uploaded as Supporting Information files. The original, signed ethics committee approval letter has been provided separately as an "Other" file.

4. **Ethics-Approved Study Protocol (Duplicate Request):** Please note that this item duplicates point 3 above. The requested protocol documents have been fully provided.
5. **Ethics Approval Date in Methods Section:** The Methods section has been amended to explicitly state the **date of Ethics Committee/IRB approval**. (Ethical approval for this study was obtained from the Research Ethics Committee of Babol University of Medical Sciences (Approval Code: IR.MUBABOL.REC.1404.106) on 4 August 2025.)
6. **Ethics Approval Letter:** A copy of the original, dated, and officially stamped/signed **IRB/Ethics Committee approval letter** has been uploaded as an **"Other" file**, in line with journal requirements.

We believe that all technical requirements have now been fully satisfied. We appreciate your time and assistance, and we look forward to the next stage of editorial assessment.

Kind regards,

Dr. Fereshteh Behmanesh

Associate Professor, Reproductive Health, PhD.
Department of Nursing and Midwifery, Babol University of Medical Sciences.
PLOS ONE Editorial Board Member
https://orcid.org/0000-0001-5837-4265
