# Supplementary material for: Sumac (Rhus coriaria L.) Capsules for the management of hypermenorrhea: Protocol for a randomized triple-blind trial
Source: PLoS One. 2026 Nov 11;21(11):e0361956. doi: 10.1371/journal.pone.0361956 (PMC13754152; doi:10.1371/journal.pone.0361956)
Supplement: Supplementary file 2 [file pone.0361956.s007.pdf]

Dear Academic Editor

Prof Samiullah Khan,

PLOS One

Thank you for your thorough review and consideration of our manuscript *"**Sumac (Rhus coriaria L.) Capsules for the Management of Hypermenorrhea: Protocol for a Randomized Triple-Blind Trial**"* (Manuscript ID: PONE-D-26-06715).

We believe that the comments and suggestions from the reviewers have helped us produce a much improved and more fully developed paper that will offer an important contribution to the field. The contributions of each author have now been indicated in the revised manuscript, and all changes are highlighted.

However, I would like to clarify a few points regarding the submission files:

1. Reviewer 4 comments: The journal sent me a PDF file with Reviewer 4's comments directly on the manuscript. Since I could not edit the PDF directly, I copied all comments into the Word version of the manuscript, provided my responses next to each comment, and then applied all revisions to the text.
2. Submitted files (three files):
    - File 1: The complete Word file including Reviewer 4's comments and my responses.
    - File 2: A clean Word file of manuscript.
    - File 3: The original PDF file containing Reviewer 4's comments, where I have written my responses next to each comment manually.

Sincerely, Corresponding author

Fereshteh Behmanesh

Associate Professor, Reproductive Health, PhD.

f.behmanesh2015@gmail.com https://orcid.org/0000-0001-5837-4265

Academic editor and reviewer(s)	Response	page
	Editor	
Revise the whole manuscript keeping in mind all reviewer's comments and submit the revised version.	Dear Editor, thank you for your guidance. We have carefully revised the entire manuscript based on all reviewers' comments and now submit the updated version.	All manuscript
Please include the following items when submitting your revised manuscript:<br>A letter that responds to each point raised by the academic editor and reviewer(s). You should upload this letter as a separate file labeled 'Response to Reviewers'.<br>A marked-up copy of your manuscript that highlights changes made to the original version. You should upload this as a separate file labeled 'Revised Manuscript with Track Changes'.<br>An unmarked version of your revised paper without tracked changes. You should upload this as a separate file labeled 'Manuscript'.	Thank you very much for your careful guidance and for providing clear instructions regarding the resubmission of our revised manuscript. We sincerely appreciate your time and the constructive approach you have taken in handling our submission.<br>In accordance with your request, we have prepared and will upload the following three files:<br>A detailed point-by-point response letter to all comments raised by the academic editor and reviewers, which we have prepared as a separate file labeled 'Response to Reviewers'.<br>A marked-up copy of the revised manuscript with all changes clearly highlighted using track changes, uploaded as a separate file labeled 'Revised Manuscript with Track Changes'.<br>A clean, unmarked version of the revised manuscript without any tracked changes, uploaded as a separate file labeled 'Manuscript'.	------

	Once again, thank you for your kind attention and valuable guidance.	
If applicable, we recommend that you deposit your laboratory protocols in protocols.io to enhance the reproducibility of your results. Protocols.io assigns your protocol its own identifier (DOI) so that it can be cited independently in the future. For instructions see: https://journals.plos.org/plosone/s/submission-guidelines#loc-laboratory-protocols. Additionally, PLOS ONE offers an option for publishing peer-reviewed Lab Protocol articles, which describe protocols hosted on protocols.io. Read more information on sharing protocols at https://plos.org/protocols?utm_medium=editorial-email&utm_source=authorletters&utm_campaign=protocols.	We appreciate the suggestion to deposit our protocol on protocols.io. However, as our manuscript describes a clinical trial protocol with detailed steps already fully presented in the Methods section and Supporting Information, we believe that the current level of detail is sufficient for reproducibility. We will consider protocols.io for future laboratory-based studies.	-----
Reviewer #1		
Dear author,<br>The authors are to be appreciated for their effort in exploring a traditional medicine–based intervention for the management of a clinically meaningful condition such as hypermenorrhea. The attempt to generate evidence in this area is valuable and relevant. The following major and minor concerns are provided with the intention of improving the scientific clarity and methodological strength of the manuscript and the trial design.	We sincerely thank the reviewer for their thoughtful and encouraging evaluation of our work. We appreciate the recognition of the relevance and value of exploring traditional medicine-based interventions for hypermenorrhea. We have carefully addressed all of the major and minor concerns raised below, and we believe the revised manuscript has been substantially strengthened as a result.	------
Major Concerns<br>1. The study involves participants with hypermenorrhea, while the control group has received a placebo. Considering the symptomatic nature of the condition, a clearer ethical justification for placebo use would strengthen the study rationale.	We thank the reviewer for raising this important ethical consideration. Participants are recruited from settings where they are not actively seeking treatment for hypermenorrhea. In the absence of the trial, they would receive no intervention for their	P 9, Par 2

	bleeding. Therefore, placebo does not place them at a disadvantage.<br><br>Regarding concomitant medications: All participants receive standard education about menstrual health. They may voluntarily use analgesics (including mefenamic acid) for dysmenorrhea or iron supplements for perceived weakness. These are not prescribed by a physician but are permitted as over-the-counter options. Their use is not restricted because randomization is expected to balance their distribution between groups. All participants record the type, dose, frequency, and duration of any analgesic or iron intake in a daily checklist. Statistical adjustment (linear mixed model) will be used to control for potential confounding. Any participant who develops clinically significant anemia or severe bleeding requiring hospitalization will be withdrawn and referred for standard medical care. This approach is ethically sound and preserves the scientific validity of the placebo-controlled design.<br><br>We have clarified this in the revised manuscript.	P 7, par 1
2. Lifestyle modification has been described in the introduction as an important primary management strategy; however, such measures do not appear to have been implemented or standardized across the study groups. Addressing this discrepancy may improve the internal consistency of the study.	We thank the reviewer for this observation. However, we would like to clarify that the sentence cited from the introduction describes the general stepwise approach in traditional Persian medicine as a historical background. It does not imply that lifestyle modification is a component of the present clinical trial. Our study is a randomized placebo-	P 3, Par 5

	controlled trial evaluating the efficacy of sumac capsules only. Lifestyle modification has not been implemented, nor is it intended to be standardized across groups, because it is outside the scope of this trial. To avoid any potential misunderstanding, we have revised the sentence in the introduction to make clear that this is a general description of traditional medicine principles and not part of the study protocol.	
3. The intervention appears to focus mainly on symptomatic management without sufficient consideration of the underlying etiology of excessive menstrual bleeding. Further clarification of the therapeutic rationale may enhance the overall scientific robustness.	We thank the reviewer for this comment. However, we respectfully clarify that our trial has not ignored the underlying etiology of hypermenorrhea. The exclusion criteria explicitly remove participants with known structural (e.g., fibroids >3 cm, polyps, adenomyosis), hormonal (e.g., thyroid disorders, irregular cycles), or neoplastic causes of heavy bleeding. Therefore, the remaining study population consists of women with idiopathic or unexplained hypermenorrhea or those with mild ovulatory dysfunction. In this context, evaluating the bleeding-reducing effect of sumac as a symptomatic treatment is scientifically valid.	-----
4. The proposed mechanism stating that the study drug induces uterine contractions leading to reduction in bleeding appears to require clearer explanation and stronger supporting references, as increased uterine activity is conventionally associated with altered bleeding patterns or dysmenorrhea.	We thank the reviewer for this careful scientific observation. We would like to clarify that the manuscript does not state that sumac induces uterine smooth muscle contractions to reduce bleeding. If this impression was given, it may have arisen from a misinterpretation of the phrase uterine vascular contraction. In fact, the astringent effect of tannins and flavonoids in sumac is primarily on	P 4, Par 5

	blood vessels (vasoconstriction), not on uterine myometrium. We have revised the relevant sentence in the manuscript to make this distinction clear and have supported it with appropriate references.	
5. The upper age limit of 45 years may include women in the perimenopausal transition phase, potentially introducing hormonal variability and heterogeneity in the sample. Justification or appropriate stratification would improve methodological clarity.	We thank the reviewer for raising this methodological point. Although the upper age limit was set at 45 years, we applied a strict inclusion criterion of regular menstrual cycles (21–42 days). Women with perimenopausal hormonal variability typically present with cycle irregularity and were therefore excluded. In addition, randomization ensures that any remaining residual heterogeneity is balanced between the intervention and placebo groups.	P 5, Par 5 & 6
6. The block size adopted for randomization appears relatively large in relation to the total sample size. Use of smaller or variable block sizes (e.g., 4–6) may have helped maintain better allocation balance and reduce predictability.	We thank the reviewer for this valuable methodological comment. We agree that using smaller or variable block sizes (e.g., 4 and 6) would have been more optimal to reduce predictability while maintaining allocation balance. However, in the present trial, allocation concealment was adequately ensured using sequentially numbered, opaque, sealed envelopes prepared by an independent individual. Moreover, the randomization sequence was generated by Random Allocation software, which produces a valid random sequence. Nevertheless, we acknowledge the reviewer's point and will consider using variable block sizes in our future trials. For the current trial, due to the fact that enrollment has	----

	already begun, we are unable to change the block size.	
7. Sample size estimation does not appear to have been supported by data from previous studies. Utilization of prior evidence could have strengthened the methodological justification.	We thank the reviewer for this important comment. In the original submission, we relied on expert clinical opinion. In the revised manuscript, we have added data from two previous clinical trials on heavy menstrual bleeding (Eshaghian et al., 2019; Mirghafourvand et al., 2015) to support the sample size calculation.	P 6, Par 3
Minor Concerns<br>1. The introduction section may benefit from further strengthening with more focused background information and relevant recent literature.	We thank the reviewer for this suggestion. The introduction has been revised accordingly.	P 3 & P 4 & P5
2. The explanation that tannins and flavonoids stimulate estrogen receptors, thereby inducing uterine contractions and reducing bleeding, may create conceptual ambiguity for readers. Further clarification with appropriate evidence is suggested.	We thank the reviewer for this careful scientific observation. We would like to clarify that the manuscript does not state that tannins and flavonoids stimulate estrogen receptors, thereby inducing uterine contractions to reduce bleeding. If this impression was given, it may have arisen from a misinterpretation of the phrase uterine vascular contraction. In fact, the astringent effect of tannins and flavonoids in sumac is primarily on blood vessels (vasoconstriction), not on uterine myometrium. We have revised the relevant sentence in the manuscript to make this distinction clear and have supported it with appropriate references.	P 4, Par 6
3. The protocol mentions the use of concomitant medications; however, the indications or criteria for their	We thank the reviewer for this comment. We have clarified that analgesics (including mefenamic acid)	P 7, Par 1

administration are not clearly specified. This may be elaborated.	are permitted at the participant's own discretion for relief of dysmenorrhea. Iron supplements are also permitted at the participant's discretion if they experience weakness. Their use is not restricted because randomization is expected to balance their distribution between groups, and statistical adjustment (linear mixed model) will be applied. These clarifications have been added to the Methods section.	
4. The method used for adverse drug reaction (ADR) assessment has not been clearly described. Mentioning the name of the validated assessment tool or causality scale would improve transparency.	We thank the reviewer for this comment. However, sumac is a commonly consumed food spice with a well-established safety profile. No serious adverse drug reactions are anticipated. Any mild adverse events (such as gastrointestinal discomfort or allergic reactions) will be reported by participants spontaneously. Given the nature of the intervention, the use of a formal causality assessment scale is not considered necessary for this trial.	------
Overall, addressing the above points may help improve the clarity, methodological rigor, and scientific strength of the manuscript and the clinical trial design.	We thank the reviewer for their thorough and constructive comments. All of the issues raised have been carefully addressed in the revised manuscript. We believe these revisions have significantly improved the clarity, methodological rigor, and scientific strength of our protocol.	------
Reviewer #2		
Introduction: Introduction can be shortened; provide a concise description of PICO.	We thank the reviewer for this suggestion. The introduction has been shortened and the following sentence has been added at the end of the	P 3 & 4 & 5

	introduction to briefly describe the PICO framework.	
Blinding / study design: There is inconsistency in reporting of blinding. Discussion mentions double-blind, while Methods describe triple-blind. This affects protocol transparency and should be corrected. Blinding is conceptually described, but operationally incomplete and needs more procedural detail to meet SPIRIT 2025 standards.	We thank the reviewer for this careful observation. The inconsistency has been corrected. Participants, care providers (those administering the capsules), and the data analyst are all blinded to group allocation. The placebo and sumac capsules are identical in shape, color, size, and packaging. Randomization codes will be kept in the sealed envelopes and broken only after the final data analysis is completed. These details have been added to the Methods section to comply with SPIRIT 2025 recommendations.	P 9, Par 3<br><br>P 6, par 5
SPIRIT Item 11 – Patient and public involvement: Details of, or plans for, patient/public involvement in design, conduct, and reporting are not adequately stated. If not planned, this should be explicitly mentioned.	We thank the reviewer for this comment. The statement regarding patient and public involvement has been included at the end of the manuscript before the References. We confirm that there was no patient or public involvement in the design, conduct, reporting, or dissemination of this trial, and this has been explicitly stated.	P 11<br><br>Before references
SPIRIT Item 15c – Adherence monitoring: Procedures for monitoring adherence are not adequately described (e.g., tablet count, return of unused medication, diary, session/visit compliance).	We thank the reviewer for raising this point. In our protocol, adherence is monitored through participant-completed daily checklists and return of unused capsule packaging at each monthly visit. Participants are instructed to record the number of capsules taken each day. Adherence will be calculated as the percentage of capsules taken relative to the expected number. This information has been added to the Methods section.	P 7, par 1

SPIRIT Item 15d – Concomitant care: Concomitant care permitted or prohibited during the trial should be clearly described.	We thank the reviewer for this comment. The description of permitted and prohibited concomitant medications has been added to the Methods section under a separate subheading (Concomitant medications)	P 7, Par 2
Discussion – limitations: Limitations section is underdeveloped. Important issues such as concomitant medication-related confounding, lack of stratification, and reliance on self-reported outcomes should be discussed more clearly.	We thank the reviewer for this comment. The limitations section has been expanded.	P 10, Par 3
Data sharing: Data sharing plan is incomplete. It should specify where, how, and under what conditions de-identified data will be available after study completion, in line with SPIRIT 2025.	We thank the reviewer for this comment. The data sharing plan has been completed in accordance with SPIRIT 2025 recommendations.	P 11, Par 1
Dissemination policy: Dissemination plan is not adequately described. The manuscript should mention how results will be communicated to participants, stakeholders, and the public, and the planned route of reporting.	We thank the reviewer for this comment. The dissemination plan has been expanded in the revised manuscript. Results will be published in a peer-reviewed journal and presented at relevant conferences. A plain language summary of the findings will also be shared with participants upon request.	P 9, Par 1<br>Ethics and dissemination
Sample size: Sample size calculation is wrong at many points; for example, with 15% attrition the final sample should be around 31.7, not 30. Effect size calculation also appears incorrect.	We thank the reviewer for identifying this error. We have revised the sample size calculation. The sample size was calculated using the formula for comparing two independent means:<br>n=(2(Z1-α/2+Z1-β)2σ2)/d2<br>With α=0.05\alpha = 0.05α=0.05 (Z-score 1.96) and 90% power (Z-score 1.28), this yielded a required	P 6, par 3

	sample size of 26 participants per group. Accounting for an estimated 15% attrition rate, the final sample size was adjusted to 30 participants per group, for a total of 60 participants.	
Statistical methods: Methods mentioned for handling missing data are inappropriate. Although protocol has already been approved by IEC, this should be rectified.	We thank the reviewer for this important methodological comment. We agree that last observation carried forward (LOCF) is no longer considered appropriate for handling missing data in longitudinal trials. Therefore, we have removed LOCF from the revised manuscript. Missing data will be handled using linear mixed models (LMM) with restricted maximum likelihood estimation, which provides unbiased estimates under the missing at random assumption. Multiple imputation will be used as a sensitivity analysis if needed. This correction has been made in the Statistical Analysis section.	P 8, par 4
Reviewer #3		
1. Line 323 in the Discussion section describes the study as a double-blind clinical trial, whereas elsewhere the manuscript presents it as a triple-blind trial. Kindly correct this inconsistency throughout the manuscript and clearly specify who will remain blinded.	We thank the reviewer for this comment. The inconsistency has been corrected. The manuscript now consistently uses triple-blind throughout.	P 9, Par 3
2. Kindly harmonize the Objectives and Outcomes sections. At present, the objectives state that the primary objective is change in menstrual blood loss measured by PBAC, while the secondary objectives include safety, tolerability, change in bleeding duration, and adverse events. However, in the Outcomes section, both intensity and duration of bleeding appear to be presented as primary outcomes.	We thank the reviewer for identifying this inconsistency. We agree that the objectives and outcomes sections should be harmonized. Therefore, we have revised the primary objective to include both bleeding intensity and duration as co-primary outcomes, consistent with the outcomes	P5, Par 2

I recommend specifying one clearly defined primary endpoint, for example, change in PBAC score from baseline to the third cycle, and retaining bleeding duration as a secondary outcome unless a co-primary outcome framework is intended.	section. We thank the reviewer for carefully identifying this inconsistency between the objectives and outcomes sections. Your sharp observation has helped us improve the clarity and internal consistency of the manuscript.	
3. Please provide the exact sample size calculation details, including the formula used, software, alpha level, power, sidedness, and the reference/source for the assumed effect size and clinically meaningful difference. This section requires correction, as sample size justification is central to a protocol paper.	We thank the reviewer for this comment. The complete sample size details are as follows: two-sided test, $\alpha = 0.05$ (Z=1.96), 90% power (Z=1.28), standard deviation of 11.0 derived from previous studies, minimum clinically meaningful difference of 10 units based on expert consensus, formula for comparison of two independent means. The sample size is 26 per group, adjusted to 30 per group after adding 15% attrition (total 60 participants).	P 6, Par 3
4. Please refer to line 234 and clarify under what criteria the co-interventions, namely mefenamic acid and iron supplementation, will be allowed. It should also be specified how the use of these co-interventions will be incorporated into the analysis, since they may influence the outcomes.	We thank the reviewer for this comment. The criteria for co-interventions have been clarified as follows:<br><br>Mefenamic acid (or any analgesic) is permitted only for relief of dysmenorrhea when the participant experiences menstrual pain.<br><br>Iron supplementation is permitted only if the participant feels weak or fatigued during menstruation.<br><br>Both are taken at the participant's own discretion, not prescribed by a physician. All use will be recorded in a daily checklist (type, dose, frequency, duration). To account for their potential effect on the primary outcome, the variable "use of	P 7, Par 1

	analgesics" and "use of iron supplements" will be entered as time-dependent covariates in the linear mixed model (LMM). This allows statistical adjustment for confounding. These clarifications have been added to the Methods section.	
5. Based on the issue raised in Comment 4, please clarify whether hemoglobin levels will be assessed at baseline, as this is relevant both clinically and analytically in participants with heavy menstrual bleeding.	We thank the reviewer for this comment. Hemoglobin levels will not be routinely assessed at baseline, as participants are recruited from non-clinical settings and are not actively seeking treatment for hypermenorrhea. Moreover, the exclusion criteria already remove participants with severe bleeding requiring hospitalization, which implicitly excludes clinically significant anemia. Therefore, routine blood testing was not considered necessary for this pragmatic trial.	------
6. As safety is included among the secondary objectives, I suggest for including routine safety investigations, such as CBC, LFT, RFT, serum electrolytes, ECG etc., at baseline and at the end of treatment to better determine the safety profile of the product, in addition to recording adverse events at each visit.	We thank the reviewer for this thoughtful suggestion. This is a preliminary, investigator-initiated trial with limited resources, designed primarily to assess the proof-of-concept efficacy of sumac on menstrual bleeding. Sumac is a commonly consumed food spice with a long history of safe human use, and no major safety concerns have been reported in previous studies. Therefore, routine laboratory safety investigations (CBC, LFT, RFT, ECG) were not included. All clinical adverse events will be systematically recorded at each visit, and any serious event will lead to immediate withdrawal and referral. We have clarified this in the revised manuscript.	------

7. Please provide further clarification on the diagnostic workup. What criteria are being used to define hypermenorrhea? Also, will all participants undergo ultrasonography before randomization, given that the exclusion criteria include structural pathology and abnormal endometrial thickness on ultrasonography?	We thank the reviewer for this comment.<br><br>Definition of hypermenorrhea: Hypermenorrhea is defined as menstrual blood loss exceeding 80 mL per cycle, corresponding to a PBAC score of ≥100, as validated in the Iranian population.<br><br>Diagnostic workup and ultrasonography: Routine ultrasonography will not be performed for all participants. If a participant has a prior ultrasound report showing structural abnormalities (e.g., fibroids >3 cm, endometrial polyps, or abnormal endometrial thickness), she will be excluded from the study.	P5, Par 6
8. Please clarify whether the placebo is adequately matched with the Sumac capsules in terms of color, texture, taste, and overall appearance, so that blinding can be effectively maintained.	We thank the reviewer for this comment. The placebo and sumac capsules are identical in shape, size, color, texture, taste, and packaging. The only difference is the code letter (A or B) on the container label, known only to the traditional medicine pharmacist who prepared the capsules. This ensures proper blinding of participants, care providers, and the data analyst.	P6, Par 5
9. The rationale for involving two different manufacturers is unclear. Kindly justify this approach. It may be preferable for the intervention and placebo to be manufactured by a single source, if feasible, to ensure better consistency and quality control.	We thank the reviewer for this comment. We apologize for the misunderstanding. The two different manufacturers refer to the suppliers of the raw materials (sumac extract and Avicel), not the final capsule production. All capsules (both sumac and placebo) were prepared by a single traditional medicine pharmacist, using identical empty capsules, the same equipment, and the same packaging process. Therefore, consistency and	P7, Par 1

	quality control are ensured. We have clarified this in the revised manuscript.	
10. The recruitment setting includes a female dormitory. Kindly justify this recruitment source, as it may introduce selection bias and affect the generalizability of the findings.	We thank the reviewer for this comment. The female dormitory is only one of several recruitment settings. Participants are also recruited from a traditional medicine clinic and a gynecology clinic. This multi-setting approach was designed to capture a more diverse sample and reduce selection bias. The inclusion of the dormitory was intended to reach women who are not actively seeking medical care for hypermenorrhea, which is ethically important for the placebo-controlled design. We have clarified this in the revised manuscript.	P 5, Par 4
Reviewer #4		
Hello Authors, the protocol is crafted good. But requires citations for some statements, and omission of unnecessary paragraphs for a protocol. Comments are given in the attached file.	We thank the reviewer for the positive feedback and constructive comments. All comments provided in the attached file have been carefully addressed. Necessary citations have been added, and unnecessary paragraphs have been omitted or shortened as suggested. We believe the revised manuscript has been improved accordingly.	----
Reviewer #5		
The recommendation can not be made to accept , because the manuscript lacks the results which are not generated till date by authors.	We thank the reviewer for the comment. However, this manuscript is submitted as a Study Protocol to PLOS One. According to the journal's submission guidelines for protocol articles, results are not required. Protocols are intended to describe the design and methodology of a trial before its	-----

	completion. The absence of results is therefore not a valid reason for rejection. We hope this clarifies the nature of the submission.	
Reviewer #6		
This protocol describes a randomized, triple-blind, placebo-controlled trial (n=60) powered at 90% to detect a 10-unit difference in PBAC scores (effect size ≈0.80). The primary objective is to evaluate whether oral Rhus coriaria capsules reduce menstrual blood loss, measured by changes in PBAC scores, compared with placebo over three cycles. Primary analysis will use repeated-measures ANOVA or mixed-effects models to assess longitudinal changes, with an intention-to-treat framework, imputation for missing data, and a two-sided α=0.05.	We sincerely thank the reviewer for this careful and accurate summary of our trial design. We greatly appreciate the time and attention given to understanding the methodological details of our protocol, including the sample size, power, primary outcome, longitudinal analysis plan, intention-to-treat framework, and missing data handling. Such thorough reading by a reviewer is encouraging and highly valued by the authors.	-----
Minor revisions:<br>1- State the statistical testing method which attains a power of 90%.	We thank the reviewer for this comment. As shown in the Sample Size section, the power of 90% was attained using the standard formula for comparison of two independent means, with α = 0.05 (two-sided), σ = 11.0, and a minimum detectable difference of 10.0 PBAC units. The corresponding Z-scores (1.96 for α, 1.28 for β) were applied. This has been clearly presented in the revised manuscript.	P 6, par 3
2- Identify the software to be used for data capture and statistical analysis.	We thank the reviewer for this comment. Statistical analysis, including linear mixed models, will be conducted using SPSS software (version 26). This has been added to the Statistical Analysis section.	P 8, Par 4
3- The handling of missing data should be revised to avoid last observation carried forward and instead rely on more	We thank the reviewer for this comment. As suggested, last observation carried forward (LOCF)	P 8, Par 4

robust approaches such as multiple imputation or model-based methods.	has been removed from the manuscript. Missing data will be handled using linear mixed models (LMM) with restricted maximum likelihood estimation under the missing at random assumption. If the proportion of missing data exceeds 10%, multiple imputation will be performed as a sensitivity analysis. This has been corrected in the Statistical Analysis section.	
4- Clarification is also needed regarding control of Type I error given repeated time-point comparisons, and whether the main inference will focus on the group-by-time interaction.	We thank the reviewer for this important methodological comment. The primary inference of this trial will be based on the group-by-time interaction term in the linear mixed model (LMM), which tests whether the change in PBAC score over time differs between the sumac and placebo groups. To control for Type I error due to repeated time-point comparisons, no adjustment for multiple comparisons will be applied for the primary analysis because the LMM with interaction term serves as a single global test. However, for secondary between-group comparisons at individual time points (post-hoc), Bonferroni correction will be used. This has been clarified in the Statistical Analysis section.	P 8, Par 4
5- Address potential non-normality of PBAC scores, incorporating baseline or other covariates to improve precision, and explicitly stating that effect sizes with confidence intervals will be reported alongside p-values.	We thank the reviewer for this valuable statistical advice. The following points have been addressed in the revised Statistical Analysis section:<br><br>Normality of PBAC scores will be assessed using the Shapiro-Wilk test and Q-Q plots. In case of significant deviation, non-parametric tests (Mann-	P 8, Par 4

	Whitney U, Wilcoxon signed-rank) will be used as sensitivity analyses, or data transformation (log or square root) will be applied. Baseline covariates including age, baseline PBAC score, and use of concomitant medications will be included as fixed effects in the linear mixed model to improve precision. For all outcomes, effect sizes with 95% confidence intervals will be reported alongside p-values. A two-sided p-value < 0.05 will be considered statistically significant. For all primary and secondary outcomes, effect sizes (Cohen's d or appropriate measure) with 95% confidence intervals will be reported alongside p-values.	
