# Supplementary material for: Sumac (Rhus coriaria L.) Capsules for the management of hypermenorrhea: Protocol for a randomized triple-blind trial
Source: PLoS One. 2026 Nov 11;21(11):e0361956. doi: 10.1371/journal.pone.0361956 (PMC13754152; doi:10.1371/journal.pone.0361956)
Supplement: Supplementary file 3 [file pone.0361956.s009.pdf]

Dear Editor

Prof Katrien G. Janin,

PLOS One

On behalf of all co-authors, I would like to express our sincere gratitude to you and the reviewers for your continued time, consideration, and thoughtful evaluation of our manuscript entitled "***Sumac (Rhus coriaria L.) Capsules for the Management of Hypermenorrhea: Protocol for a Randomized Triple-Blind Trial***" (Manuscript ID: PONE-D-26-06715).

We are particularly grateful for the reviewers' careful reassessment of our revised manuscript and their constructive, insightful comments. We greatly appreciate the time and expertise they have devoted to helping us strengthen our work. Their feedback has been invaluable in improving the clarity, transparency, and methodological presentation of our study protocol.

We are sincerely thankful for your continued guidance throughout the review process and for the reviewers' thoughtful contributions. We hope that the revised manuscript now adequately addresses all remaining concerns and will be suitable for publication in PLOS ONE.

Thank you again for your time, consideration, and continued support.

Sincerely, Corresponding author

Fereshteh Behmanesh

Associate Professor, Reproductive Health, PhD.

Department of Nursing and Midwifery, Babol University of Medical Sciences. https://orcid.org/0000-0001-5837-4265

**Academic reviewer(s)**	**Response**	**page**
Reviewer #1		
All the queries raised during the review have been adequately addressed by the authors. However, some methodological concerns remain. Since this is a protocol-led study that has already been initiated, these aspects may not be feasible to modify at this stage. Therefore, the authors may consider acknowledging such methodological issues as limitations of the study.<br>For example, regarding baseline haemoglobin screening, the exclusion criterion specifies only clinically significant anaemia. The implications of this criterion may be acknowledged as a limitation.	We sincerely thank the reviewer for their positive assessment and for their time and valuable contributions throughout the review process.<br>Thank you very much for this constructive suggestion. We have acknowledged the absence of baseline haemoglobin measurement as a limitation of the study and added a corresponding statement to the Limitations section of the manuscript.	P 10, Limitations
Similarly, with regard to the change in the missing-data handling method from Last Observation Carried Forward (LOCF) to multiple imputation, the authors have clarified that re-approval is not required but that the modification should be intimated to the IEC. This procedural aspect may also be appropriately mentioned, where relevant, as a limitation or methodological consideration.	We sincerely thank the reviewer for their careful attention to ethical considerations in research and for emphasizing the importance of transparent reporting of methodological changes.<br>We would like to clarify that no change has been made to the originally planned missing-data handling method. The reference to LOCF in the previous version was an inadvertent reporting error, and multiple imputation has been the intended approach from the outset. Had any actual change to the original protocol been made in response to the reviewer's comments, we would have informed the Institutional Ethics Committee accordingly. However, in this case, only a reporting error has been corrected, and the planned methodology remains unchanged.<br>We appreciate the reviewer's valuable comments and commitment to research integrity.	-----

Overall, the manuscript may be considered after incorporating a clear acknowledgement of these methodological limitations.	We sincerely thank the reviewer for their careful evaluation and constructive suggestions. We have carefully considered the methodological concerns raised and revised the manuscript accordingly, while clarifying the rationale for our prespecified methodological decisions. We greatly appreciate the reviewer's valuable input, which has helped us improve the clarity and transparency of the manuscript.	----
Reviewer #2		
This protocol article on herbal medicine for Gynecological disease condition, the authors have made necessary changes, and the manuscript can be published.	We sincerely thank the reviewer for the positive and encouraging comments and for recognizing our efforts to improve the manuscript. We greatly appreciate the reviewer's careful evaluation and constructive feedback.	----
Reviewer #3		
Several of the comments raised or substantial revision suggested by the reviewer have been addressed well by the authors.	We sincerely thank the reviewer for their careful evaluation of our manuscript and for acknowledging our efforts to address the comments and suggested revisions. We greatly appreciate the reviewer's constructive feedback, which has helped us improve the clarity and quality of our manuscript.	----
Reviewer #6		
All comments have been adequately addressed.	We greatly appreciate the reviewer's careful evaluation and recognition of the improvements made in our manuscript.	-----
