# Supplementary material for: Sumac (Rhus coriaria L.) Capsules for the management of hypermenorrhea: Protocol for a randomized triple-blind trial
Source: PLoS One. 2026 Nov 11;21(11):e0361956. doi: 10.1371/journal.pone.0361956 (PMC13754152; doi:10.1371/journal.pone.0361956)
Supplement: Supplementary file 4 [file pone.0361956.s001.docx]

Babol University of Medical Sciences

*Deputy of Research and Technology*

*Research Proposal*

_______________________________________

**Evaluating the efficacy of sumac (Rhus coriaria L) capsules for hypermenorrhea**

**Proposal Abstract**

Abnormal menstrual bleeding is one of the common problems among women, caused by disturbances in the regulation of menstrual cycles, and it can affect both quality of life and overall health. Although many traditional medicine sources have described sumac as having astringent and pro-coagulant properties, and some scattered studies have suggested potential effects on uterine bleeding, definitive confirmation or rejection of these effects requires human clinical trials. Moreover, hormonal medications commonly prescribed to control bleeding may cause considerable side effects such as headaches, an increased risk of thromboembolism, and contraindications in patients with cardiovascular and liver diseases, hypertension, and diabetes. In addition, public interest in complementary therapeutic methods—due to their lower cost, easier accessibility, and fewer side effects—further emphasizes the need to investigate medicinal plants such as sumac.

Therefore, this study was designed to evaluate the effectiveness of sumac capsules in reducing moderate to heavy menstrual bleeding. In this clinical trial, after approval of the research proposal by Babol University of Medical Sciences and obtaining the necessary permissions from the Ethics Committee and the Research Council, the researcher will refer to relevant settings including female student dormitories, gynecology clinics, and the traditional medicine clinic. After explaining the study objectives and obtaining informed consent, convenience sampling will be performed. Participants who meet the inclusion criteria (60 individuals), aged 18 to 45 years, will be randomly assigned to either the intervention group or the control group. Participants will consume two capsules daily—sumac (intervention) or Avicel (control)—12 hours apart, during the first seven days of three consecutive menstrual cycles. The capsules are prepared as follows: an aqueous extract (grade A++) of sumac fruit is purchased from Giah-Kala Company, and the total flavonoid content is determined. A specific amount of the extract (equivalent to 500 mg of dried sumac fruit) will then be placed into capsules by a traditional medicine pharmacist. The placebo capsules, containing Avicel purchased from Behboud-Shimi Company under the Gloria brand, will also be filled to 500 mg by the same pharmacist. A total of 42 capsules of sumac or placebo will be packaged for use over the three menstrual cycles, and coded containers will be prepared to ensure the double-blind design of the study.

To assess bleeding intensity, participants will complete the Higham chart or PBAC visual chart before the intervention and throughout the three cycles. If needed, mefenamic acid and iron supplements will be prescribed for both groups, and the amount used will be recorded. The study instruments include a demographic–social questionnaire and the PBAC chart, which will be provided to participants along with the necessary instructions.

**Introduction**

Abnormal menstrual bleeding is a medical condition characterized by irregular menstrual cycles, including excessive menstrual blood loss. Menstrual bleeding exceeding 80 milliliters per cycle or lasting longer than seven days is considered abnormal, a condition referred to as hypermenorrhea (1, 2). The prevalence of abnormal menstrual bleeding worldwide varies and depends on multiple factors, including geographical region, cultural context, socioeconomic status, access to and effectiveness of medical care, and environmental factors (1, 3).

Complications of abnormal menstrual bleeding include iron deficiency, physical symptoms such as pain and fatigue, and, consequently, a reduced quality of personal and social life among women of reproductive age (4).

The most common causes of abnormal menstrual bleeding include pregnancy-related conditions, structural uterine pathologies (such as fibroids, endometrial polyps, and adenomyosis), ovulatory disorders, and neoplastic disorders, which, through excessive blood loss, can lead to anemia, coagulopathy, acidosis, hypothermia, and coagulation abnormalities (4, 5). Hormonal disorders and platelet-related blood abnormalities, such as vascular diseases and coagulation disorders, can also influence this condition (6, 7).

Based on these varying factors, different treatment approaches are considered for hypermenorrhea. The first-line treatment for hypermenorrhea is pharmacological therapy.

Pharmacological treatments include the use of nonsteroidal anti-inflammatory drugs, hormone therapy, progestin-containing medications, and progesterone-releasing intrauterine devices. In cases of inadequate response to medical therapy, surgical approaches such as hysterectomy or endometrial ablation are employed (8). In some cases, emergency treatment is required, such as in the presence of severe bleeding, which may result from acute blood loss (hemorrhage) (9, 10).

In traditional Iranian medicine texts, excessive menstrual flow, prolonged menstrual duration, and bleeding outside the menstrual period are referred to as efrāṭ-e ṭamth or kasrat-e ṭamth (11).The general principles of treatment in traditional Iranian medicine are based on lifestyle modification, with nutrition considered a key component. If no improvement is observed, the next steps recommended are pharmacological treatment followed by manual or procedural interventions (12). The efficacy of certain pharmacological treatments has been investigated in clinical trials, which have demonstrated positive effects (13). In the first step, hematinic treatments consisting of Khabth al-Hadid derivatives are prescribed to the patient. In the second step, herbal astringents such as quince paste, myrtle, pomegranate flower, and plantain are administered, depending on availability and cost-effectiveness (11, 14).

Another substance mentioned in traditional Iranian medicine for its effectiveness in controlling and treating abnormal uterine bleeding is sumac. Reduced blood viscosity due to increased heat and fluidity can be one of the causes of bleeding. During menstruation, the released blood is described as thin, yellowish, warm, and burning, and it exits under pressure. An appropriate intervention in this condition is to lower the “heat” of the blood. Therefore, foods considered “cooling” in traditional Iranian medicine are used—these include pomegranate, barberry, unripe grape, rhubarb, sumac, and vinegar. Causes of uterine bleeding include lesions such as ulcers, masses, trauma, difficult childbirth, or any factor that damages the uterus. In these cases, alongside treating the underlying cause, the consumption of astringent foods is recommended. These substances, which have a constrictive effect, help contract the uterus, close the vessels, and stop bleeding. Such foods include quince, apple, lentils, sumac, sour grapes, pomegranate, barberry, oak, Russian olives, and vinegar. (15).

Sumac is a plant from the pistachio family, with the scientific name Rhus coriaria L (16, 17). The flowers of the sumac shrub are dense, clustered, and honey-colored. Sumac primarily grows in rocky areas and on hard, rugged soils (18).

The nature of sumac is cold and dry in the second degree, and it possesses actions and properties such as astringent, restraining, stomach-tanning, strengthening the warm organs, and reducing bile. It has traditionally been used to treat nausea and vomiting caused by bile, bleeding, and chronic diarrhea (19).

In traditional Iranian medicine, sumac has been regarded as a preventive agent against cardiovascular diseases and is consumed alongside certain foods (20). Many recent findings regarding the effects of sumac on digestive disorders, diarrhea, as well as its antiseptic and analgesic properties, are consistent with the observations of traditional Iranian medicine scholars (21). Sumac has also long been used as a food seasoning and for strengthening the stomach and gums, reducing or stopping bleeding, and preventing infectious diseases (22). One of the therapeutic uses of sumac in traditional Iranian medicine is its application in the preparation of sumac porridge (Somāqīyeh), which has been used to treat conditions such as indigestion and diarrhea (23), nausea, nosebleeds, blood-related joint pain, hiccups, and bleeding from hemorrhoidal vessels (24). According to written Iranian medical sources, this plant has also been used for uterine disorders (25, 26). Studies have shown that sumac increases platelet distribution width (27). It can also influence bleeding time, prothrombin time, and activated partial thromboplastin time, suggesting its potential as an adjuvant therapy for bleeding control. In a study by Salsali and colleagues titled Dietary Recommendations for Abnormal Uterine Bleeding in Traditional Iranian Medicine, which reviewed classical Iranian medical texts such as Qanun fi al-Tibb, Tibb Akbari Arzani, Ma‘alijat Aghili Khorasani, Sharh al-Asbab Samarkandi, and Aksir-e Azam Nazem Jahan, it was reported that the consumption of astringent foods can be effective in reducing menstrual bleeding. One such food is sumac (15).

The active compound in sumac is tannins (28). Sumac is also rich in magnesium, calcium, tannins, flavonoids, and other polyphenolic compounds. Its astringent properties are attributed to malic acid, tannic acid, gallic acid, and tannins. Tannins and flavonoids stimulate estrogen receptors, the smooth muscles of the myometrium, and uterine vascular contraction (14). Experimental and preclinical studies suggest that polyphenols and tannins present in Rhus coriaria may influence inflammatory and vascular pathways. However, the relevance of these mechanisms to uterine bleeding in humans remains uncertain and requires confirmation through well-designed clinical trials (29).

A study titled Anti-inflammatory Properties of Sumac (Rhus coriaria L.) Powder Combined with Iron Sulfate on Hematological Indices and Oxidative Stress in Young Female Athletes with Non-anemic Iron Deficiency reported that sumac powder did not enhance the effect of iron supplementation and even had a negative impact (30). Momenimovahed and Khalajinia (2019) demonstrated that ethanolic extract of sumac fruit significantly reduced the expression of inflammatory genes IL-1β and IL-18 in LPS-stimulated synovial cells, showing notable anti-inflammatory effects (31). Since these cytokines play a role in many inflammatory processes, including endometrial inflammation and abnormal uterine bleeding, it can be inferred that sumac may indirectly reduce uterine bleeding by inhibiting inflammatory responses. However, to confirm this effect definitively, direct clinical studies on uterine health are necessary. Additionally, a systematic review of five studies investigating sumac’s effects on oxidative stress found that four studies reported significant reductions in malondialdehyde (MDA) levels and increases in total antioxidant capacity (TAC) and paraoxonase-1 (PON1). Regarding inflammation, one study reported no significant differences in TNF-α or IL-6 levels between intervention and control groups (32).

Given references in traditional medicine texts to the potential pro-coagulant effects of *Rhus coriaria* and the limited experimental studies suggesting hemostatic activity, well-designed human trials are required to clarify its clinical effects on menstrual bleeding. Moreover, hormonal therapies commonly used for heavy menstrual bleeding are associated with adverse effects and contraindications in certain populations, including increased thromboembolic risk and limitations in women with cardiovascular, metabolic, or hepatic conditions. Alongside growing interest in complementary therapies due to their accessibility, lower cost, and perceived safety, preliminary evidence from Iranian medical sources supports a potential role for sumac in the management of uterine bleeding. Therefore, this randomized, triple-blind, placebo-controlled trial is designed to evaluate the efficacy and safety of sumac capsules in women with moderate to heavy menstrual bleeding, with the aim of generating preliminary clinical evidence to inform future confirmatory studies.

**Objectives**

The primary objective of this trial is to evaluate whether oral administration of *Rhus coriaria* capsules reduces menstrual blood loss, measured by changes in PBAC scores, compared with placebo over three consecutive menstrual cycles.

Secondary objectives include assessing the safety and tolerability of sumac capsules, evaluating changes in bleeding duration, and recording the frequency and type of adverse events during the intervention period.

**Methods**

***Study Design and Setting***

This study will be conducted as a triple-blind, parallel-group randomized clinical trial with random allocation of participants into two groups: intervention and control. This manuscript follows the SPIRIT 2013 guidelines (Supplementary file 1). The trial was registered in the Iranian Clinical Trial Registry (Code: IRCT20180218038783N12) on 5 October 2025. The recruitment period for this study started on 22 October 2025 and is expected to continue until October 2027. Participants will be recruited from a traditional medicine clinic, a gynecology clinic, and the female dormitory of Babol University of Medical Sciences. Time schedule of enrolment, interventions, assessments, and visits for participants is shown in Fig 1. The study design also follows the consolidated standards of CONSORT clinical trial reports (Fig 2).

***Eligibility Criteria***

Eligible participants will include women aged 18–45 years with menstrual blood loss >80 mL per cycle, assessed using the Pictorial Blood Loss Assessment Chart (PBAC) (score ≥100), consent to participate in study, a regular menstrual cycle of 21–42 days, and literacy.

Exclusion criteria will include gynecological pathologies (ovarian cysts, endometrial polyps, neoplasms), irregular menstrual cycles, chronic medical conditions (hypertension, uncontrolled diabetes, renal, hepatic, cardiovascular, or thyroid disorders), use of anticoagulants or hormonal contraceptives, smoking or alcohol consumption, abnormal endometrial thickness on ultrasonography, first-degree family history of cancer, uterine fibroids >3 cm (submucosal or intramural), known allergy to sumac, adherence to a special diet in the previous three months, and severe bleeding requiring hospitalization.

Withdrawal criteria will include <80% treatment adherence, pregnancy or breastfeeding, and unwillingness to continue participation.

***Randomization, Allocation Concealment, and Blinding***

Participants will be enrolled using convenience sampling and randomly assigned in a 1:1 ratio to the intervention or placebo group using block randomization (block size = 10) generated by Random Allocation software. Allocation concealment will be ensured using sequentially numbered, opaque, sealed envelopes prepared by an independent individual. Random sequence generation, participant enrollment, and group assignment will be performed by separate personnel. The researchers, participants, and data analyst will remain blinded to group allocation throughout the study.

To ensure allocation concealment and preserve the integrity of randomization, sealed opaque envelopes containing the allocation assignments will be used. Based on the predetermined sample size, 60 sequentially numbered envelopes will be prepared, each enclosing a card indicating the random allocation. At the time of participant enrollment, envelopes will be opened in sequence according to the order of recruitment to determine group assignment.

***Implementation***

After selecting participants based on the inclusion criteria and obtaining informed consent, they will be randomly allocated into two groups. The intervention group will receive capsules containing an aqueous extract of sumac fruit (A++ grade), obtained from Giah Kala Company, with the total flavonoid content determined. Each capsule will be equivalent to 500 mg of dried fruit. The control group will receive placebo capsules containing 500 mg of Avicel, purchased from Behboud Shimi Company (Gloria brand). All capsules will be prepared and coded by a pharmacist specialized in traditional medicine who is not involved in participant recruitment. Participants will take two capsules daily, at 12-hour intervals, during the first seven days of menstruation, for three consecutive menstrual cycles. If required, mefenamic acid and iron supplements will be prescribed to both groups and recorded.

***Primary and Secondary Outcome***

The primary outcomes of this study are the intensity and duration of menstrual bleeding, assessed using the Pictorial Blood Loss Assessment Chart (PBAC). Bleeding intensity will be evaluated based on the total PBAC score, while bleeding duration will be determined by the number of bleeding days recorded in the PBAC chart. Outcomes will be assessed at baseline and after one, two, and three menstrual cycles following initiation of the intervention.

Secondary outcomes include the occurrence, type, and severity of any adverse effects associated with sumac capsule consumption, which will be recorded prospectively throughout the study period.

***Assessment and Recording of Adverse Events***

Adverse events will be assessed at each monthly follow-up visit through structured interviews and participant self-reporting. Participants will be instructed to report any gastrointestinal symptoms, allergic reactions, dizziness, nausea, or other unexpected symptoms during the intervention period.

Adverse events will be categorized according to severity (mild, moderate, severe) and their potential relationship to the intervention (unlikely, possible, probable). Serious adverse events will be reported immediately to the ethics committee.

***Instruments***

Menstrual blood loss will be assessed using the PBAC (Higham Chart), a validated and reliable tool, completed at baseline and during each menstrual cycle. The Pictorial Blood Loss Assessment Chart (PBAC) was originally developed by Higham, O’Brien, and Shaw in 1990 as a semi-quantitative tool for assessing menstrual blood loss (33). This chart is a simple, non-laboratory visual assessment tool for the evaluation of menstrual bleeding. The scoring system is based on the visual inspection of sanitary pads, tampons, and the presence of blood clots. The chart has a two-dimensional structure, with the number of menstrual days represented on the horizontal axis and the degree of blood-soaked pads on the vertical axis. Light staining (spotting or minimally soiled pads) was assigned a score of 1, moderate staining a score of 5, and heavy saturation a score of 20. In addition, the passage of small clots (approximately the size of a small coin) was scored as 1, large clots (approximately the size of a large coin) as 5, and each episode of flooding (soiling of underwear or free flow of blood into the toilet) was scored as 5. At the end of menstruation, the frequency of each item was multiplied by its corresponding score, and the total PBAC score was calculated by summing all values. A PBAC score of 100 or higher corresponds to a menstrual blood loss exceeding 80 mL. The Higham chart is a validated instrument for quantifying menstrual blood loss and The Higham chart is a valid instrument, and its reliability was reported as r = 0.88 in the study by Mohamadirizi et al in Iran (34).

Demographic, socioeconomic, and obstetric data will be collected at baseline using a structured questionnaire including age, body mass index (BMI), educational level, place of residence, employment status, and satisfaction with household income, as well as obstetric information such as the number of pregnancies, deliveries, and abortions, and the method of contraception, were collected. Data related to abnormal uterine bleeding included menstrual cycle characteristics, namely cycle regularity, intermenstrual interval, duration of bleeding, presence of dysmenorrhea, and volume of menstrual blood loss.

***Sample Size***

Based on expert clinical opinion, a minimum clinically significant difference of 10 units in PBAC scores between groups will be assumed. With a standard deviation of 6.5, an effect size of 0.80, a 95% confidence level, and 90% power, the required sample size will be 27 participants per group. Allowing for a 15% attrition rate, 30 participants per group will be recruited.

***Statistical Analysis***

All analyses will be done according to the intention-to-treat principle. Continuous variables will be summarized as means ± standard deviations or medians with interquartile ranges, as appropriate. Changes in PBAC scores over time will be analyzed using repeated-measures analysis of variance or linear mixed-effects models, with group, time, and group-by-time interaction terms included. Between-group differences at each time point will be assessed with independent t-tests or non-parametric equivalents. Missing data will be handled using multiple imputation methods if the proportion of missing data exceeds 5%; otherwise, last observation carried forward will be applied. A two-sided p-value <0.05 will be considered statistically significant.

References

1.	Borzutzky C, Jaffray J. Diagnosis and management of heavy menstrual bleeding and bleeding disorders in adolescents. JAMA pediatrics. 2020;174(2):186-94.

2.	De Sanctis V, Soliman A, Tzoulis P, Daar S, Di Maio S, Millimaggi G, et al. Hypomenorrhea in adolescents and youths: normal variant or menstrual disorder? Revision of literature and personal experience. Acta Bio Medica: Atenei Parmensis. 2022;93(1):e2022157.

3.	Ibrahim PM, Samwel EL. Prevalence of Heavy Menstrual Bleeding and Its Associated Factors Among Women Attending Kilimanjaro Christian Medical Centre In Northern Eastern, Tanzania: A Cross-Sectional Study. The East African Health Research Journal. 2023;7(1):1.

4.	Kaunitz AM, Barbieri RL, Levine D, Falk SJ. Approach to abnormal uterine bleeding in nonpregnant reproductive-age women. Internet) Walthman (MA): UpToDate. 2015.

5.	Ash T. Massive transfusion protocol. Archives of Hematology Mini Reviews and Reviews. 2023;8(1):004-7.

6.	Kulkarni R. Improving care and treatment options for women and girls with bleeding disorders. European journal of haematology. 2015;95:2-10.

7.	Bradley LD, Gueye N-A. The medical management of abnormal uterine bleeding in reproductive-aged women. American journal of Obstetrics and Gynecology. 2016;214(1):31-44.

8.	Rodriguez MB, Lethaby A, Jordan V. Progestogen‐releasing intrauterine systems for heavy menstrual bleeding. Cochrane Database of Systematic Reviews. 2020(6).

9.	Isono W, Tsuchiya A, Okamura A, Honda M, Saito A, Tsuchiya H, et al. Unilateral uterine artery embolization and Bakri tamponade balloon insertion in the treatment of acute puerperal uterine inversion: a case report. Journal of Medical Case Reports. 2022;16(1):190.

10.	Kaganov H, Ades A. Uterine fibroids: investigation and current management trends. Australian Family Physician. 2016;45(10):722-5.

11.	Tansaz M, Memarzadehzavareh H, Qaraaty M, Eftekhar T, Tabarrai M, Kamalinejad M. Menorrhagia management in Iranian traditional medicine. Journal of evidence-based complementary & alternative medicine. 2016;21(1):71-6.

12.	Falahat F, Tavakkoli M, Mokaberinejad R, Ayati S, Feyzabadi Z. Natural treatments of oligomenorrhea based on persian medicine. The Iranian Journal of Obstetrics, Gynecology and Infertility. 2018;21(Supplement):55-66.

13.	Fesharaki G, Zafarghandi N. Comparison of effects of pomegranate peel and Mefenamic acid consumption on the treatment of Menorrhagia: A Triple-Blind Randomized Controlled Trial. 2019.

14.	Ashteany E-h, Vahid Dastjerdi M, Tabarrai M, Nejatbakhsh F, Sadati Lamardi SN, Rahmani A, et al. Effectiveness of Persian Golnar on Excessive Menstrual Bleeding in Women with Abnormal Uterine Bleeding, Compared to Tranexamic Acid: A Triple-Blind, Randomized Equivalence Trial. Evidence-Based Complementary and Alternative Medicine. 2023;2023.

15.	Salsali G. Food recommendations for abnormal uterine bleeding in Traditional Iranian medicine on the basis of Hemorheology. Int J Ayurved Med. 2019;10(1):1-6.

16.	Ghahreman A, Okhovvat A. Matching the old medicinal plant names with scientific terminology. vol. 1. Farsi ed. Tehran University Press, Iran; 2004.

17.	Mozaffarian V. A dictionary of Iranian plant names: Latin, English, Persian: Farhang Mo'aser; 1996.

18.	Ahmad H, Ahmad F, Hasan I, Ahmad S. Unani description of Sumaq (Rhus coriaria Linn.) and its scientific report. Global Journal of Medical Research. 2013;13(7):75-8.

19.	Khoshkharam M, Shahrajabian MH, Singh RB, Sun W, Magadlela A, Khatibi M, et al. Sumac: A functional food and herbal remedy in traditional herbal medicine in the Asia. Functional foods and nutraceuticals in metabolic and non-communicable diseases: Elsevier; 2022. p. 261-6.

20.	Zargham H, Zargham R. Tannin extracted from Sumac inhibits vascular smooth muscle cell migration. McGill Journal of Medicine: MJM. 2008;11(2):119.

21.	Mohammadi S, Zarei M, Zarei MM, Salehi I. Effect of hydroalcoholic leaves extract of Rhus Coriaria on pain in male rats. Anesthesiology and pain medicine. 2016;6(1).

22.	Mosaffa-Jahromi M, Kiani H. Alshamel fi Sana’at Tebbi’at: A Comprehensive Book on the Materia Medica. Iranian Journal of Medical Sciences. 2016;41(3 Suppl):S20.

23.	Razi B. Kholasat ol-Tajarob. The Institute for Medical History-Islamic and Complementary Medicine, Tehran University of Medical Sciences, Tehran. 2000:134-53.

24.	MA A. Tibb-i Akbari (Akbari's Medicine). Qom: Jalal al-Din Publications. 2008;2:690-5.

25.	Ibn-e-Sina A. Al-qanun fit-tib [The canon of medicine]. Beirut, Lebanon: Alaalami Beirut lib Press; 2005.

26.	Aghili Shirazi S. Makhzan Al-adviyah (the Storehouse of medicaments). Tehran: Tehran University of Medical Sciences. 2009.

27.	Capcarova M, Slamecka J, Abbas K, Kolesarova A, Kalafova A, Valent M, et al. Effects of dietary inclusion of Rhus coriaria on internal milieu of rabbits. Journal of animal physiology and animal nutrition. 2012;96(3):459-65.

28.	Khodabakhsh M, Mahmoudinia M, Mousavi Bazaz M, Hamedi SS, Hoseini SS, Feyzabadi Z, et al. The effect of plantain syrup on heavy menstrual bleeding: A randomized triple blind clinical trial. Phytotherapy Research. 2020;34(1):118-25.

29.	Payranj M, Moradi M, Rakhshandeh H, Ghavami V, Farahi SMM, Najafabadi MG, et al. Investigating the Impact of Sumac Capsules on Postpartum Bleeding among Women at Risk of Bleeding: a three-blind randomized clinical trial. Journal of Pharmacopuncture. 2024;27(4):287.

30.	Amirsasan R, Zakeri N, Gargari BP, Alizadeh S. The effect of anti-inflammatory properties of sumac powder (Rhus coriaria L.) along with ferrous sulfate on blood markers and oxidative stress of young athletic girls with non-anemic iron deficiency (NAID). Sport Sciences for Health. 2024:1-9.

31.	Momenimovahed Z, Khalajinia Z. Unwillingness to have Vaginal Delivery among Iranian Women: A Qualitative Study. Current Womens Health Reviews. 2025;21(1):E240124226088.

32.	Bahari H, Zeraattalab-Motlagh S, Sajadi Hezaveh Z, Namkhah Z, Golafrouz H, Taheri S, et al. The Effects of Sumac Consumption on Inflammatory and Oxidative Stress Factors: A Systematic Review of Randomized Clinical Trials. Current Pharmaceutical Design. 2024;30(27):2142-51.

33.	Higham JM, O'brien P, Shaw R. Assessment of menstrual blood loss using a pictorial chart. BJOG: An International Journal of Obstetrics & Gynaecology. 1990;97(8):734-9.

34.	Mohamadirizi S, Kordi M, Shakeri MT, Fadardi JS, Hafizi L. Relationship between Job Stress with Menstrual Bleeding Pattern among Midwives. Hayat/Ḥayāt. 2012;18(5).
