# Supplementary material for: Sumac (Rhus coriaria L.) Capsules for the management of hypermenorrhea: Protocol for a randomized triple-blind trial
Source: PLoS One. 2026 Nov 11;21(11):e0361956. doi: 10.1371/journal.pone.0361956 (PMC13754152; doi:10.1371/journal.pone.0361956)
Supplement: Supplementary file 5 [file pone.0361956.s002.docx]

#### بسمه تعالي

## دانشگاه علوم پزشکي بابل

## *معاونت تحقيقات و فناوري*

## *طرح پيشنهادي تحقيق*

### *مشخصات طرح پژوهشي*

| نوع پايان نامه/طرح ارائه شده | طرح ارائه شده به مرکز تحقيقات |
| --- | --- |
| عنوان طرح: | بررسی اثربخشی کپسول سماق (Rhus coriaria L) بر هایپرمنوره |
| عنوان لاتین طرح | Evaluating the efficacy of sumac (Rhus coriaria L) capsules for hypermenorrhea |
| سازمان متبوع | دانشگاه علوم پزشکی بابل |
| محل اجرای طرح | دانشگاه علوم پزشکی بابل |
| نوع طرح | کارآزمایی بالینی |

### خلاصه پروپوزال

### خونریزی غیرطبیعی قاعدگی یکی از مشکلات شایع زنان است که به‌دلیل اختلال در تنظیم چرخه‌های قاعدگی ایجاد می‌شود و می‌تواند کیفیت زندگی و سلامت فرد را تحت تأثیر قرار دهد. با توجه به اینکه در منابع متعدد طب سنتی از خواص قابض و پیش‌انعقادی سماق یاد شده و برخی مطالعات نیز به‌طور پراکنده اثرات آن را بر خونریزی رحمی مطرح کرده‌اند، اما برای تأیید یا رد قطعی این اثرات، انجام آزمایشات بالینی انسانی ضروری است. از سوی دیگر، مصرف داروهای هورمونی که عموماً برای کنترل خونریزی تجویز می‌شوند، عوارض قابل‌توجهی مانند سردرد، افزایش احتمال ترومبوآمبولی و محدودیت مصرف در بیماران مبتلا به بیماری‌های قلبی‌–عروقی، کبدی، فشار خون بالا و دیابت دارند. همچنین استقبال عمومی از روش‌های درمانی مکمل به دلیل هزینه کمتر، دسترسی آسان‌تر و عوارض محدودتر، ضرورت بررسی گیاهانی مانند سماق را تقویت می‌کند. به همین دلیل این پژوهش با هدف ارزیابی اثربخشی کپسول سماق بر کاهش خونریزی متوسط تا شدید قاعدگی طراحی شده است. در این کارآزمایی بالینی، پس از تصویب طرح در دانشگاه علوم پزشکی بابل و اخذ مجوزهای لازم از کمیته اخلاق و شورای پژوهشی، پژوهشگر به محیط‌های مربوطه از جمله خوابگاه دانشجویان دختر، درمانگاه‌های زنان و کلینیک طب سنتی مراجعه کرده و پس از توضیح اهداف مطالعه و اخذ رضایت‌نامه آگاهانه، نمونه‌گیری آسان انجام می‌شود. سپس شرکت‌کنندگان (60 نفر) به‌صورت تصادفی در دو گروه مداخله و کنترل قرار می‌گیرند. نمونه ها روزانه دو کپسول سماق (مداخله) و آویسل (کنترل) با فاصله ۱۲ ساعت، طی هفت روز اول سه سیکل قاعدگی مصرف می‌کنند. روش تهیه کپسول ها به این صورت است که عصاره آبی (درجه A++) میوه سماق از شرکت گیاه کالا تهیه و میزان فلاونوئیدهای کل تعیین می‌شود. سپس مقدار مشخصی از عصاره (معادل ۵۰۰ میلی‌گرم میوه خشک سماق) توسط داروساز طب سنتی در کپسول ریخته خواهد شد. کپسول های دارونما، حاوی آویسل است که از شرکت بهبودشیمی با برند گلوریا خریداری و به اندازه 500 میلی گرم توسط متخصص داروساز طب سنتی در کپسول ها ریخته خواهد شد. سپس تعداد 42 کپسول سماق یا دارونما برای مصرف سه سیکل قاعدگی در ظرف مخصوص ریخته شده و روی ظرف ها کد گزاری انجام می شود تا پژوهش به‌صورت دوسوکور انجام گیرد. برای سنجش شدت خونریزی، جدول هیگام یا چارت بصری PBAC قبل از شروع و طی سه سیکل توسط افراد تکمیل می‌شود. در صورت نیاز، مفنامیک‌اسید و مکمل آهن برای هر دو گروه تجویز شده و میزان مصرف آنها نیز ثبت می‌گردد. ابزارهای مطالعه شامل پرسشنامه مشخصات فردی–اجتماعی و جدول PBAC است که همراه با توضیحات لازم در اختیار شرکت‌کنندگان قرار می‌گیرد.

### واژه های کلیدی: سماق- هایپرمنوره- قاعدگی- خونریزی-کارآزمایی بالینی

### *کليات و مقدمه*

| **عنوان** | **متن** |
| --- | --- |
| مقدمه و بیان مساله | خونریزی غیرطبیعی قاعدگی یک وضعیت طبی است که به تنظیم نامناسب دوره‌های قاعدگی زنانه اشاره دارد. خونریزی‌های قاعدگی بیش از 80 میلی‌لیتر و یا بیشتر از 7 روز غیرطبیعی است (1).  هایپر منوره یکی از انواع خونریزی های غیر طبیعی رحمی است که به خونریزی بیش از 80 سی سی در هر سیکل قاعدگی اشاره دارد. شیوع خونریزی غیرطبیعی قاعدگی در دنیا متغیر است و بسته به عوامل مختلفی از جمله منطقه جغرافیایی، فرهنگ، وضعیت اقتصادی و اجتماعی، میزان بهره‌وری درمانی و عوامل زیست محیطی متفاوت است(2, 3). اما بطور کلی 30 درصد از زنان در سنین باروری از هایپرمنوره رنج میبرند(4).  از جمله عوارض خونریزی غیرطبیعی قاعدگی میتوان به فقر آهن، علائم جسمی مانند درد و خستگی و بدنبال آن کاهش کیفیت زندگی فردی و اجتماعی زنان در سنین باروری اشاره نمود (5).  شایع ترین علل خونریزی غیرطبیعی قاعدگی ، شرایط مرتبط با بارداری، آسیب شناسی ساختاری رحم (مانند فیبروم، پولیپ آندومتر، آدنومیوز)، اختلال تخمک گذاری و اختلالات نئوپلازی است که با از دست‌دادن خون زیاد منجر به کم خونی، انعقاد خون، اسیدوزیس، هایپوترمی و کوآگولوپاتی می‌شود (5, 6). همچنین اختلالات هورمونی و مشکلات پلاکتی خون مانند بیماری وریدی و اختلالات انعقادیمیتواند این وضعیت را تحت تاثیر قرار دهد(7). بر اساس این عوامل متفاوت، روش‌های درمانی مختلفی برای هایپرمنوره در نظر گرفته می‌شود. گام اول درمان هایپرمنوره استفاده از داروها است(4, 8). درمانهای دارویی شامل استفاده از داروهای ضد التهاب غیراستروئیدی، هورمون درمانی و داروهای حاوی پروژسترون و IUD های آزاد کننده پروژسترون است و در صورت عدم پاسخ مناسب به دارودرمانی، روش های جراحی مانند هیسترکتومی یا ابلیشن آندومتر مورد استفاده قرار می گیرد(9).  در برخی موارد نیاز به درمان اورژانسی است، مانند مواجهه با خونریزی شدید که ممکن است به علت از دست دادن خون(هموراژی) منجر شود(10, 11).  در کتب طب ایرانی، به زیادی جریان یافتن خون قاعدگی، افزایش طول مدت قاعدگی و خونریزی در زمان غیرقاعدگی، افراط طمث یا کثرت طمث اطلاق می گردد(12). اصول کلی درمان در طب ایرانی بر پایه اصلاح سبک زندگی است که تغذیه از ارکان مهم محسوب می‌شود. قدم بعدی در صورت عدم بهبودی، درمان با دارو و سپس اعمال یداوی توصیه می‌شود (13). اثربخشی برخی از درمان‌های دارویی توسط کارآزمایی های بالینی مورد مطالعه قرارگرفته‌اند و اثرات مثبتی نشان‌داده ‌است(14). در قدم اول، درمان های خون‌ساز متشکل از مشتقات خبث الحديد برای بیمار تجویز می گردد و قدم دوم، قابضات گیاهی مانند رب به، مورد، گلنار و بارهنگ با توجه به شرایط دسترسی و مقرون به صرفه‌بودن تجویز می‌گردد(12, 15).  یکی دیگر از موادی که در طب ایرانی به اثر بخشی آن برای کنترل و درمان خونریزی غیرطبیعی رحمی اشاره شده، سماق است. **کاهش ویسکوزیته خون به دلیل افزایش دما و همچنین سیالیت آن می‌تواند یکی از علل خونریزی باشد. خون آزاد شده در دوران قاعدگی رقیق و زرد رنگ، گرم و سوزان است و با فشار خارج می‌شود. یک اقدام مناسب در این شرایط، پایین آوردن دمای خون است. بنابراین، از مواد غذایی استفاده می‌شود که در طب سنتی ایران به عنوان «خنک‌کننده» شناخته می‌شوند - غذاهایی که در آنها از انار، زرشک، غوره، ریواس، سماق و سرکه استفاده** می‌شود. علل خونریزی رحم شامل ضایعاتی مانند زخم، توده، ضربه و سخت‌زایی و هر عاملی که باعث آسیب به رحم شود، می‌باشد. در این موارد، علاوه بر درمان علت خونریزی، مصرف غذاهای قابض توصیه می‌شود. این مواد که خاصیت قابض دارند، باعث جمع شدن رحم و بسته شدن رگ و قطع خونریزی می‌شوند. این غذاها شامل به، سیب، عدس، سماق، انگور ترش، انار، زرشک، بلوط، زیتون روسی و سرکه هستند (16).  **سماق گیاهی از خانواده پسته‌ایان با نام علمی** Rhus coriaria L **می‌باشد(17, 18). گل‌های درختچه سماق خوشه‌ای و متراکم و حنایی رنگ است. محل رویش سماق عمدتاً مناطق صخره‌ای و با زمین‌های سخت است (19). طبیعت سماق سرد و خشك در درجه دوم و دارای افعال و خواصی از قبیل: رادع، قابض، دابغ معده، مقوى احشاى حارّه، رافع صفرا می‌باشد و جهت تهوع و استفراغ ناشی از صفرا و خونریزی‌ها و اسهال مزمن بکار می‌رفته است(20).** در طب ایرانی، سماق به عنوان یک ماده پیشگیری‌کننده از بیماری‌های قلبی مورد توجه بوده و همراه بعضی غذاها مصرف میشود(21). **بسیاری از یافته های جدید در زمینه تاثیر سماق بر مشکلات گوارشی، اسهال و اثر ضد‌عفونی کنندگی و ضد درد آن با نظرات حکمای طب ایرانی مطابقت دارند(22)**.**سماق همچنین از دیرباز به عنوان چاشنی غذا و نیز برای تقویت معده و لثه‌ها، کاهش و یا قطع خونریزی و پیشگیری از امراض عفونی مصرف می‌شده ‌است(23).** یکی از مصارف درمانی سماق در طب ایرانی کاربرد آن بواسطه تهیه آش سماق (سماقیه) می‌باشد که در درمان بیماری‌هایی مانند: فساد هضم و اسهال (24)، حالت تهوع، خون دماغ، درد مفاصل دموی (25)، سکسکه، خونریزی عروق بواسیر کاربرد داشته‌است (20). **بر اساس منابع مکتوب پزشکی ایرانی از این گیاه، حتی بیماری‌های رحمی استفاده می‌شده‌است(26, 27).**  **مطالعات نشان دادند که سماق عرض توزیع پلاکت را افزایش می‌دهد(28).** **هم چنین با تغییر در مدت زمان خونريزي، زمان پروترومبین، زمان ترومبوپلاستین جزئی می‌تواند داروی کمکی مناسبی برای کنترل خونریزی ارائه دهد. در مطالعه صلصالی و همکاران با عنوان توصیه های غذایی برای خونریزی غیرطبیعی رحم در طب ایرانی با بررسی کتب طب ایرانی از جمله قانون فی طب، طب اکبری ارزانی، معلجات عقیلی خراسانی، شرح الاسباب سمرقندی، اکسیر اعظم ناظم جهان بیان میکند که مصرف مواد غذایی قابض می‌تواند در کاهش خونریزی قاعدگی موثر باشد. یکی از این موارد سماق است (16). ماده موثره ای که در سماق وجود دارد** Tanninsنام دارد (29). سماق همچنین سرشار از منیزیم، کلسیم، تانن، فلاونوئیدها و سایر ترکیبات پلی فنلی است. خواص قابض آن به اسید مالیک، اسید تانیک، اسید گالیک و تانن نسبت داده می‌شود. تانن و فلاونوئیدها باعث تحریک گیرنده‌های استروژن، عضلات صاف میومتر و انقباض عروق رحم می‌شوند [15]. سماق با داشتن ترکیبات پلی‌فنولی، تانن و فلاونوئید، خواص قابض و ضدالتهابی را نشان می‌دهد. با تأثیر بر اندوتلیوم عروقی، فعال‌سازی نیتریک اکسید سنتاز اندوتلیال TNF-alpha را آغاز می‌کند که منجر به انقباض عروق و فعال‌سازی مسیر سیکلواکسیژناز می‌شود و در نهایت خونریزی رحم را کاهش می‌دهد (30). نتایج **حاصل از مطالعه‌ای با عنوان** اثر خواص ضدالتهابی پودر سماق (Rhus coriaria L.) همراه با سولفات آهن بر شاخص‌های خونی و استرس اکسیداتیو در دختران جوان ورزشکار با کمبود آهن غیرآنمیک نشان داد که پودر سماق نه تنها اثر مکمل آهن را بهبود نبخشید، بلکه تأثیر منفی بر آن داشت (31). نتایج مطالعه Momeni و همکاران (2019)، نشان داد عصاره الکلی میوه سماق با کاهش معنادار بیان ژن‌های التهابی IL-1β و IL-18 در سلول‌های سینوویال تحریک‌شده با LPS^[1]^، اثرات ضدالتهابی قابل توجهی نشان داده است. (32) از آنجا که سایتوکاین‌های مذکور در بسیاری از فرایندهای التهابی از جمله التهاب اندومتر و خونریزی غیرطبیعی رحم نقش دارند، می‌توان چنین استنباط کرد که سماق ممکن است به‌صورت غیرمستقیم و از طریق مهار پاسخ‌های التهابی، در کاهش خونریزی رحمی مؤثر واقع شود. با این حال، برای تأیید قطعی این اثر، انجام مطالعات بالینی مستقیم در زمینه سلامت رحم ضروری است. در یک مطالعه مروری سیستماتیک نیز پنج مطالعه اثرات سماق را بر استرس اکسیداتیو گزارش کردند که چهار مورد از آنها کاهش قابل توجهی در سطح مالون دی‌آلدئید (MDA) و افزایش ظرفیت آنتی‌اکسیدانی کل (TAC) و پاراکسوناز 1 (PON1) را نشان دادند. در مورد التهاب، یک مطالعه هیچ تفاوت معنی‌داری در سطح فاکتور نکروز تومور-α (TNF-α) و اینترلوکین-6 (IL-6) بین گروه‌های مداخله و کنترل گزارش نکرد.  **با توجه به اشاره بسیاری از متون طب سنتی به اثرات پیش انعقاد این عصاره ها و هم چنین مطالعات ذکر شده برای رد یا تایید اثرات بالینی سماق بر خونریزی قاعدگی، آزمایشات انسانی لازم است.**  **همچنین با توجه به عوارض مصرف داروهای هورمونی از جمله سردرد، افزایش خطر ترومبوآمبلی و ممنوعیت مصرف داروهای هورمونی در افراد با سابقه بیماری عروق مغزی و کرونر، اختلالات کبدی، فشار خون، دیابت و ... ، همچنین علاقه مردم به استفاده از روش‌های درمانی مکمل بدلیل صرفه اقتصادی، در دسترس بودن و عوارض کمتر و وجود شواهدی در کتب طب ایرانی مبنی بر اثرگذار بودن سماق بر خونریزی رحمی، این مطالعه با هدف تعیین اثربخشی کپسول سماق بر خونریری متوسط و شدید قاعدگی انجام می‌شود.** |
| بررسی متون | **1- مطالعه‌اي كه توسط رادمان و همكاران(2023) با عنوان بررسي تاثیر انار، مورت، بلوط و سماق را بر کنترل خونریزی بر روی موش‌های صحرایی نر ویستار انجام شد. در اين مطالعه مدت زمان خونريزي، زمان پروترومبین، زمان ترومبوپلاستین جزئی در موش‌هاي صحرايي اندازه‌گيري شد. با توجه به نتايج مشاهده شده در مقایسه گروه‌هاي آزمايش با گروه شاهد، این عصاره ها می توانند داروی کمکی مناسبی برای کنترل خونریزی ارائه دهند. اين مطالعه بر روي سی و دو عدد ویستار 8 تا 10 ماهه نر با وزن 200 تا 250 گرم در شرایط آزمایشگاهی استاندارد با دسترسی آزاد به آب و غذای کافی نگهداری شدند، انجام شد. موش‌هاي صحرايي به چهار گروه 1) استفاده موضعي از عصاره‌ها روي زخم دم، اندازه‌گيري زمان خونريزي، 2) تزريق داخل صفاقی عصاره گياهان، اندازه‌گيري پروترومبين و زمان ترومبوپلاستین جزئی در نمونه های خون گرفته شده از قلب ، 3) گروه كنترل يك (زخم‌های بدون استفاده موضعی از عصاره گیاهان، اندازه‌گیری زمان خونریزی، 4) گروه کنترل دو(بدون تزریق داخل صفاقی عصاره ها، اندازه‌گيري پروترومبين و زمان ترومبوپلاستین جزئی). نتایج نشان داد میانگین زمان خونریزی در گروه کنترل به طور معنی‌داری بیشتر از گروه مداخله بود. میانگین زمان پروترومبین در گروه کنترل تفاوت معنی داری با گروه مداخله نداشت. میانگین زمان ترومبوپلاستین جزئی در گروه کنترل به طور معنی‌داری کوتاه‌تر از گروه مداخله بود. با توجه به اثرات انعقادی قابل قبول عصاره های پوست و گل انار، مورت، بلوط و سماق در مقایسه با گروه کنترل می توان نتیجه گرفت که این عصاره ها می توانند داروهای کمکی مناسبی برای کنترل خونریزی باشند(33).**  2- مطالعه‌ای توسط افندک و همکاران(2022) با عنوان: «بررسی تاثیر پودر سماق بر علائم بالینی، هایپرآندروژنیسم، پروتئین واکنشگر C ، پروفایل قند و چربیهای خون در زنان مبتلا به سندرم تخمدان پلی کیستیک( PCOS ) » انجام شد. اين مطالعه يک کارآزمایی بالینی تصادفی کنترل شده دو سوکور بوده که بر روی زنان 20 تا 40 ساله مراجعه کننده به بیمارستان شهید اکبرآبادی انجام گرفت. معیارهای ورود به مطالعه: ابتلای بیمار به سندرم تخمدان پلی کیستیک بر اساس معیارهای روتردام، شامل: تاخیر بیشتر از 35 روز یا کمتر از 8 دوره خونریزی در سال علائم بالینی شامل پرمویی بالاتر از 8، اکنه و تاسی مدل مردانه و ریزش موی سر علائم بیوشیمیایی افزایش آندوژنیسم تخمدان پلی کیستیک 12 یا بیشتر از 12، فولیکول در هر تخمدان با قطر 9-2 میلی متر و یا افزایش حجم تخمدان به بیشتر از 310 میلی لیتر، تمایل به شرکت در مطالعه ، محدوده سنی 20 تا 40 سال. معیارهای خروج از مطالعه شامل: مصرف داروهای ضد انعقاد، سیگار، داشتن بیماری کلیوی، دیابت، بیماری کبد، اختلالات قلبی عروقی، کم کاری تیروئید، هیپرپرولاکتینمی، سندرم کوشینگ و هیپرپلازی مادرزادی آدرنال، پیروی از یک رژیم غذایی خاص در 3 ماه گذشته، مصرف داروهای مؤثر بر حساسیت به انسولین یا لیپیدهای هورمونی، شامل گلوکوکورتیکوئیدها، داروهای تحریک تخمک گذاری، ضد چاقی، ضد دیابت، ضد فشار خون، ضد استروژن، آنتی آندروژنیک در 3 ماه گذشته (به استثنای داروهای معمول بیماران)، عدم تمایل به شرکت در مطالعه، مصرف مکمل‌های غذایی ، داشتن پرسشنامه ناقص، اعمال هرگونه تغییر در پروتکل درمانی و داروها و داشتن پایبندی به درمان کمتر از 80 درصد، حساسیت و آلرژی به سماق. ابزارهای مورد استفاده در مطالعه شامل: یک یادآوری رژیم غذایی 24 ساعته (24 ساعت) برای ارزیابی میزان مصرف غذا قبل و بعد از مداخله تکمیل شد. دریافت درشت مغذی‌ها و ریز مغذی‌ها توسط برنامه Nutritionist IV ارزیابی شد. هیرسوتیسم با استفاده از پرسشنامه فریمن-گالوی، آکنه توسط سیستم درجه بندی آکنه جهانی GAGS و منظم بودن قاعدگی با استفاده از پرسشنامه و ثبت فاصله بین دو خونریزی در بیماران در ابتدا و انتهای مداخله ارزیابی شد. درصد چربی بدن به روش ebody اندازه گیری شد. پرسشنامه بین المللی فعالیت بدنی IPAQ قبل و بعد از مطالعه در هر دو گروه برای ارزیابی سطح فعالیت بدنی استفاده شد. نمونه خون وریدی (10 سی سی) از بیماران پس از 12 ساعت ناشتایی قبل و بعد از مداخله جهت اندازه گیری پارامترهای بیوشیمیایی گرفته شد. حجم نمونه در هر گروه 37 نفر در نظر گرفته‌شد که حجم نهایی با فرض 15 درصد ریزش نمونه 44 نفر در نظر گرفته شد. بیماران به طور تصادفی به دو گروه سماق (44 نفر) و دارونما (44 نفر) تقسیم شدند. گروه سماق روزانه سه کپسول 1 گرمی سماق و گروه دارونما یک کپسول مشابه سماق، اما از نشاسته با همان مقدار ، روزانه به مدت 12 هفته مصرف کردند. تجزیه و تحلیل اطلاعات با استفاده ازنرم افزار SPSS نسخه 25 انجام شد. در پایان مطالعه، 39 نفر در گروه سماق و 36 نفر در گروه دارونما باقی ماندند. میانگین سنی بیماران در گروه دریافت کننده سماق و دارونما به ترتیب 23 و 26 سال بود. مصرف مکمل با پودر سماق تأثیری بر فشار خون سیستولیک و دیاستولیک نداشت. میزان تغییرات قند خون ناشتا، انسولین ناشتا، حساسیت به انسولین و مقاومت به انسولین پس از مداخله در گروه سماق نسبت به مقادیر قبل از مداخله و گروه دارونما به طور معنی‌داری کاهش یافت. میزان تغییرات hs-CRP پس از مداخله در گروه سماق در مقایسه با مقادیر قبل از مداخله و گروه دارونما به طور معنی‌داری کاهش یافت. میزان تغییرات تری گلیسیرید، نسبت LDL به HDL و کلسترول پس از مداخله در گروه سماق نسبت به مقادیر قبل از مداخله و نسبت به گروه دارونما به طور معنی داری کاهش یافت. با این حال، سطح LDL به طور قابل توجهی بین دو گروه پس از مداخله تغییر نکرد . حساسیت به انسولین و HDL بعد از مداخله در گروه سماق نسبت به مقادیر قبل از مداخله و در مقایسه با گروه دارونما به طور معنی‌داری افزایش یافت. نتایج این مطالعه نشان داد که مصرف سماق می‌تواند علائم التهاب، دیابت و چربی خون را در زنان مبتلا به PCOS کاهش دهد، اما تأثیر آن بر هورمون‌های جنسی و علائم بالینی نیاز به مطالعه بیشتری دارد (34).  3- مطالعه‌ای توسط میرعنایت و همکاران (2020) با هدف : «تعیین اثر مکمل پودر سماق بر نشانگرهای متابولیک در بزرگسالان مبتلا به سندروم متابولیک» انجام شد. این مطالعه یک کارآزمایی بالینی متقاطع تصادفی سه سو کور و کنترل شده با دارونما بود که در مرکز تحقیقات غدد و متابولیک اصفهان(IEMRC) از نوامبر 2020 تا ژوئن 2021 انجام شد. معیارهای ورود به مطالعه شامل: محدوده سنی 20 تا 70 سال که مایل به شرکت در کارآزمایی بودند. طبق تعریف "برنامه ملی آموزش کلسترول، پنل درمانی بزرگسالان III  " (NCEP/ATP III)، سندرم متابولیک (MetS) به‌عنوان داشتن سه یا بیشتر از معیارهای زیر تعریف شده است: 1) دورکمر در زنان بیشتر از88 در مردان بیشتر از 108، 2) 2) تری‌گیلیسیرید بیشتر از 150 میلی گرم در دسی لیتر، 3) غلظت لیپوپروتئین-کلسترول با چگالی بالا در زنان کمتر از 50 و در مردان کمتر از 40 میلی گرم در دسی لیتر، 4) فشار سیستولیک بیشتر از 130 یا دیستولیک بیشتر از 85 و 5)قند خون ناشتا بیشتر از 100 میلی گرم در دسی لیتر. معیارهای خروج شامل: سابقه بیماری قلبی-عروقی،کلیوی، کبدی، مشکلات تیروئید، دیابت، سکته مغزی، بارداری و شیردهی، پیروی از الگوی غذایی خاص، مصرف دارویی که بر اشتها، فشار خون،التهاب، سطح چربی یا قندخون، متابولیسم چربی یا کربوهیدرات تاثیرگذار باشد. استفاده از مکمل‌های مولیت ویتامین مینرال، اسیدهای چرب امگا3 یا داروهای گیاهی. داشتن عفونت کرونا . در ابتدای مطالعه شرکت کنندگان بصورت تصادفی در دو گروه کپسول سماق و دارونما قرارگرفتند. به منظور تصادفی سازی و کور کردن از وب سایت www.randomization.com استفاده شد. در مرحله اول مداخله گروه اول کپسول سماق حاوی 500 میلی گرم پودر سماق و گروه دو کپسول دارونما با همان مقدار لاکتوز دریافت کردند که از نظر شکل،رنگ و اندازه کپسول سماق و دارونما یکسان بودند. داروها دو بار در روز بعد از شام و ناهار به مدت 6 هفته مصرف شد. تعداد نمونه در گروه مکمل سماق 23 نفر و در گروه دارونما 24 در نظر گرفته شد. تجزیه و تحلیل داده‌ها با استفاده از نرم افزار SPSS نسخه 18 انجام شد. 81 درصد شرکت کنندگان زن و 19 درصد مرد بودند. بر اساس نتایج مکمل سماق فشار سیستولیک را به میزان 5 میلی متر جیوه کاهش داده است.مکمل سماق میتواند از افزایش اندک در تری گلیسیرید بطور معناداری جلوگیری کند. طبق یافته‌های این مطالعه نتایج نشان داد که مکمل سماق بطور قابل توجهی فشار خون سیستولیک را در بزرگسالان مبتلا به سندروم متابولیک کاهش داده است (35).  4- مطالعه‌ای توسط اسحاقیان و همکاران (2018) با عنوان « بررسی اثر کندر و زنجبیل به عنوان درمان مکمل بر خونریزی شدید قاعدگی (HMB) در زنان در سنین باروری» انجام گرفت. این کارآزمایی بالینی تصادفی، دوسوکور، کنترل شده با دارونما در دو کلینیک سرپایی زنان در شهر اصفهان (ایران) از مرداد 1395 تا دی ماه 1397 انجام شد. معیارهای ورود شامل سن 18 تا 45 سال، خونریزی قاعدگی بیش از 80 میلی لیتر در هر سیکل و یا بیش از هفت روز بر اساس PBAC(Pictorial Blood Assessment Chart)، سیکل قاعدگی منظم از 22 تا 35 روز، معاینات زنان و زایمان طبیعی بدون عفونت یا کیست و توده، تست پاپ اسمیر طبیعی در طی سال گذشته. معیارهای خروج شامل بارداری و شیردهی، ضخامت غیرطبیعی آندومتر یا مورفولوژی رحم طبق سونوگرافی تراس واژینال، فیبروم رحم بزرگتر از 3 سانتی متر، کیست تخمدان، هموگلوبین کمتر از 5/10، سابقه واکنش آلرژیک به زنجبیل، کندر یا ایبوپروفن، مصرف داروهای هورمونی (داروهای ضدبارداری) یا سایر داروهای موثر بر خونریزی قاعدگی مانند آسپیرین. ابزارهای مورد استفاده در مطالعه شامل: جمع آوری اطلاعات دموگرافیک با استفاده از پرسشنامه، جدول PBAC یک مقیاس اندازه‌‌گیری که میزان از دست دادن خون قاعدگی را بر اساس ظاهر بررسی حوله‌های لکه دار تعداد پد یا تامپون استفاده شده ارزیابی میکند.کیفیت زندگی بیماران با استفاده از پرسشنامه منوراژی اندازه‌گیری شد. این پرسشنامه علائم خاص خونریزی و کیفیت زندگی را براساس درد، مشکلات خونریزی از طریق لباس، زندگی کاری، فعالیتهای اوقات فراغت و فعالیت جنسی ارزیابی میکند.تعداد نمونه در هر گروه 19نفر در نظر گرفته شد که با احتمال ریزش نمونه34 نفر در هرگروه در نظر گرفته‌شد.136 بیمار مورد مطالعه قرار گرفتند. از این تعداد 26 نفر بر اساس معیارهای مطالعه واجد شرایط نبودند. 8 بیمار حاضر به شرکت در مطالعه نبودند.در طول ماه اول 23 بیمار درمان را قطع کردند و در طول ماه دوم 13 بیمار مطالعه را ادامه ندادند. همه بیماران 200میلی گرم قرص ایبوپروفن(شرکت رازی، تهران، ایران)دریافت کردند. بیماران گروه کندر کپسول حاوی 300میلی گرم پودر کندر و 200 میلی گرم لاکتوز بدون آب، بیماران گروه زنجبیل کپسول حاوی 300 میلی گرم پودر زنجبیل و 200 میلی گرم لاکتوز بدون آب و بیماران گروه دارونما کپسول حاوی 200 میلی گرم لاکتوز بدون آب که از نظر دارویی شبیه دو داروی دیگر بود، دریافت کردند. مصرف دارو بصورت 3 بار در روز و به مدت 7 روز بود. همه بیماران برای دو سیکل قاعدگی مداوم تحت درمان قرارگرفتند. تحلیل اطلاعات و داده‌ها با نرم افزار SPSS نسخه 23 انجام شد. براساس نتایج تفاوت معناداری از نظر تغییر در نمره PBAC در پایان ماه اول و ماه دوم مداخله وجود داشت. میانگین مدت خونریزی قاعدگی گروه کندر و گروه زنجبیل در طول مطالعه بطور معناداری کاهش یافت. طبق یافته‌های این مطالعه نتایج نشان داد به نظر میرسد کندر و زنجبیل به عنوان مکمل NSAID در درمان خونریزی شدید قاعدگی در زنان در سنین باروری موثر میباشد (36).  5- مطالعه‌ای توسط خدابخش و همکاران(2017) با هدف: «تعیین تاثیر شربت چنار بر خونریزی شدید قاعدگی» انجام شد. این مطالعه یک کارآزمایی کنترل شده سه سو کور است که بر روی 68 زن مبتلا به خونریزی شدید قاعدگی مراجعه کننده به کلینیک دانشگاه علوم پزشکی مشهد، کلینیک طب سنتی، بیمارستان امام رضا(ع) و درمانگاه بانوان قائم(عج)، از سپتامبر 2017 تا اکتبر 2018 انجام شد. معیارهای ورود به مطالعه شامل: زنان بین 36 تا 45 سال، آزمایشات طبیعی زنان و پاپ اسمیر، هموگلوبین: 11-14 دسی لیتر، عدم بیماری مزمن (فشار خون، دیابت کنترل نشده، بیماری احتقانی قلب و دیسکرازی خون)، عدم وجود لوپوس اریتماتوز سیستمیک، عدم مشکلات تیروئید، هیپرپرولاکتینمی، شیردهی، عدم تمایل به استفاده از داروهای ضد بارداری خوراکی، عدم سابقه ترومبوآمبولی، عدم وجود اجسام خارجی داخل رحم و عدم وجود فیبروم (معیارهای ورود توسط متخصص زنان تایید شد). معیار خروج از مطالعه شامل: حاملگی و هرگونه عوارض جانبی احتمالی در طول مطالعه بود. پرسشنامه‌ها و ابزارهای استفاده شده در مطالعه شامل: تاریخچه کامل از مشخصات دموگرافیک، الگوهای قاعدگی و سابقه پزشکی از بیماران گرفته شد و حجم خونریزی قاعدگی با استفاده از نمودار ارزیابی تصویری خون (PBAC) طی یک سیکل بررسی شد. در نهایت حجم نمونه با در نظر گرفتن 20 درصد ریزش نمونه،34 بیمار در هر گروه مداخله و کنترل در نظر گرفته‌شد. گروه مداخله کپسول دارونما همراه با شربت چنار (5 سی سی) هر 8 ساعت و گروه کنترل کپسول مفنامیک اسید (250 میلی گرم) همراه با شربت دارونما (5 سی سی) هر 8 ساعت دریافت کردند. از بیماران خواسته شد در 5 روز اول قاعدگی و طی سه سیکل هر 8 ساعت یک بار از یک کپسول همراه با 5 سی سی شربت استفاده کنند و جدول PBAC را بر اساس حجم خونریزی کامل کنند. تجزیه و تحلیل داده‌ها با استفاده از نرم افزار SPSS نسخه 16 انجام شد. در گروه چنار یک بیمار به دلیل عدم استفاده از دارو، سه بیمار به دلیل مصرف داروهای شیمیایی و دو بیمار به دلیل بارداری از مطالعه خارج شدند. در گروه مفنامیک اسید یک بیمار به دلیل بارداری، سه بیمار به دلیل مصرف نامنظم دارو و دو بیمار به دلیل مصرف داروهای شیمیایی از مطالعه خارج شدند. در نهایت، اطلاعات 56 بیمار مورد تجزیه و تحلیل قرار گرفت. در هر دو گروه، میانگین شدت و مدت خونریزی قبل و بعد از مداخله بر اساس اندازه‌گیری‌های مکرر ANOVA مقایسه شد. نتایج نشان داد که مدت و شدت خونریزی پس از مداخله در هر دو گروه کاهش یافته است. بر اساس نتایج این مطالعه، شربت چنار می تواند به اندازه کپسول مفنامیک اسید در کاهش مدت خونریزی قاعدگی موثر باشد (29).  6-مطالعه‌ای که توسط کریمیان و همکاران(2015) با هدف: «مقایسه اثر کپسول بابونه و کپسول مفنامیک اسید بر میزان خونریزی دوران قاعدگی» انجام شد. این مطالعه کارآزمایی بالینی تصادفی سه سوکور در سال 1390 بر روی 90 نفر از دانشجویان ساکن در خوابگاه های دانشگاه علوم پزشکی کاشان انجام شد. معیارهای ورود به مطالعه شامل: مجرد بودن و نداشتن سابقه بیماری طبی خاص، سابقه مصرف دارو، سابقه مصرف قرص های جلوگیری از بارداری و نداشتن حساسیت دارویی یا گیاهی بود. در این مطالعه واحدهای پژوهش ابتدا پرسشنامه شامل اطلاعات فردی، وضعیت سیکل های قاعدگی و بررسی میزان خونریزی قاعدگی بود را تکمیل می کردند. جهت تعیین حجم خونریزی از آزمون استاندارد هیگام استفاده شد. جهت تعیین شدت درد از خط کش مك گیل استفاده‌شد. حجم نمونه با توجه به مطالعات مشابه 90 نفر در نظر گرفته شد. افراد به صورت تصادفی در دو گروه قرار گرفتند. به گروه اول کپسول مفنامیك اسید 250 میلی‌گرم هر 8 ساعت در طی دوره قاعدگی داده شد و به گروه دوم نیز کپسول بابونه ساخت شرکت باریج اسانس250 میلی‌گرم هر 8 ساعت داده شد. در نهایت تجزیه و تحلیل داده ها با استفاده از نرم افزار SPSS نسخه 16 استفاده‌شد. بر اساس نتایج بابونه باعث کاهش حجم خونریزی در سیکل قاعدگی می‌شود ولی این کاهش مقدار خونریزی در اثر بابونه در سیکل دوم نسبت به سیکل اول تفاوتی نداشت. کپسول مفنامیك اسید نیز باعث کاهش حجم خونریزی شد و مانند بابونه اختلافی بین دو سیکل در این زمینه وجود نداشت. نتایج نشان داد که اختلاف معنی داری بین دو داروی بابونه و مفنامیك اسید در کاهش دادن حجم خونریزی وجود نداشت. کپسول بابونه می تواند جایگزین مناسبی برای داروهای شیمیایی در درمان خونریزی قاعدگی باشد. لذا می‌توان از این گیاه در جهت درمان این مشکل زنان و ارتقاء سلامت آنان استفاده کرد(37).  7-مطالعه‌اي توسط ميرغفوروند و همكاران (2015) با هدف: «مقايسه تاثير ويتاگنوس و بذر كتان بر ميزان خونريزي قاعدگي» انجام شد . اين مطالعه يک کارآزمایی بالینی تصادفی کنترل شده سه سوکور بوده که بر روی زنان مراجعه‌کننده به مراکز بهداشتی درمانی شهر تبريز از اوايل آبان ماه سال ۱۳۹۲ تا اوايل شهريور ۱۳۹۳ انجام گرفت. اين مطالعه بخشي از يک پژوهش بزرگ است که بررسي خونريزي قاعدگي جزء اهداف فرعي مطالعه بود. معیارهای ورود شامل سن بین 18 تا 45 سال عدم وجود خونریزی قاعدگی، داشتن سیکل منظم 22 تا 35 روزه، داشتن سواد خواندن و نوشتن و تمایل به شرکت در مداخله. معیارهای خروج شامل بارداری و شیردهی، مصرف داروهای هورمونی پیشگیری از بارداری در حال حاضر، عدم سیکل قاعدگی منظم طی 6 ماه گذشته، بی سواد بودن، داشتن علائم یا ترشحات عفونی، وجود میوم شناخته‌شده رحمی، مصرف داروی آگونیست و آنتاگونیست دوپامین (لودوپا، آمفتامين ها، بروموکريپتين، پرگوليد، کابرگولين، متوکلوپراميد) در ۶ ماه اخير، سابقه خانوادگي سرطان پستان و ساير سرطان ها در خانواده درجه يک، مصرف داروهايي نظیر ضد افسردگي، ديگوکسين، متيل دوپا، اسپيرونولاکتون، کلروپرومازين، دانازول و تاموکسيفن و سابقه آسم يا آلرژی بود. ابزارهای مورد استفاده در مطالعه شامل: پرسشنامه مشخصات فردی-اجتماعی، جدول هیگام برای ارزیابی میزان خونریزی قاعدگی(PBAC) جهت تکمیل در روزهای قاعدگی به همراه توضیحات جهت تکمیل در اختیار شرکت کنندگان قرارگرفت. حجم نمونه با استفاده از نرم افزار G-power 43 نفر برای هر گروه محاسبه شد که با در نظر گرفتن 20 درصد ریزش برای هر گروه 53 نمونه در نظر گرفته‌شد. در نهایت در هر گروه 53 نفر شرکت کننده قرار گرفت. گروه اول که بذر کتان بصورت پودر با دوز 25 گرم برای مصرف روزانه به علاوه دارونمای ویتاگنوس در اختیارشان قرار گرفت. گروه دوم روزانه 1 عدد قرص ویتاگنوس 2/3 تا 8/4 میلی گرم تهیه شده از شرکت گل‌دارو به علاوه دارونمای بذر کتان که حاوی آرد گندم بود در اختیارشان قرار گرفت. گروه سوم دارونمای ویتاگنوس همرنگ و هم شکل قرص ویتاگنوس و بسته‌های حاوی 25 گرم آرد گندم جهت مصرف روزانه دريافت كردند. مصرف قرص‌ها بصورت روزانه بعد از غذا از اول تا پایان چرخه قاعدگی بود. اطلاعات با استفاده از نرم افزار SPSS نسخه 21 تحلیل شد. در این مطالعه 159 نفر مورد تجزیه و تحلیل قرارگرفتند. میانگین سن شرکت‌کنندگان 6/32 بود. میانگین سن منارک شرکت‌کنندگان 7/12 بود. بر اساس نتایج میانگین نمره هیگام در گروه بذرکتان از 73/90 در قبل از مداخله به 4/74 در پایان ماه اول و به 0/71 در پایان ماه دوم رسید.در گروه ویتاگنوس از نمره 6/85 در قبل از مداخله به 8/72 در پایان ماه اول و به 9/67 در پایان ماه دوم بعد از مداخله رسید. در گروه کنترل نمره هیگام 0/73 در شروع مطالعه به 3/72 در ماه اول و به 1/70 در ماه دوم بعد از مداخله رسید. طبق یافته‌های این مطالعه نتایج نشان داد بذر کتان و ویتاگنوس در درمان خونریزی موثر است و عوارض جانبی کمتری نسبت به داروهای شیمیایی دارد (38). |

### *اهداف و فرضيات*

| **عنوان** | **متن** |
| --- | --- |
| هدف اصلی | تعیین اثربخشی کپسول سماق (Rhus coriaria L) بر هایپرمنوره |
| هدف فرعی | مقایسه تغییر میانگین نمره شدت خونریزی قاعدگی در گروه مداخله (کپسول سماق) و دارونما پس از مداخله  مقایسه تغییر میانگین مدت خونریزی قاعدگی در دو گروه مداخله (کپسول سماق) و دارونما پس از مداخله  تعیین عوارض احتمالی ناشی از مصرف کپسول سماق در گروه مداخله (کپسول سماق) و دارونما |
| هدف کاربردی | در صورت تاثیر کپسول سماق بر کاهش خونریزی دوران قاعدگی می‌توان از این گیاه ارزان و در دسترس در درمان زنان مبتلا به این بیماری استفاده کرد. |
| فرضیه | میانگین نمره شدت خونریزی قاعدگی در دو گروه مطالعه متفاوت است.  میانگین مدت خونریزی قاعدگی در دو گروه مطالعه متفاوت است. |
| سوال | عوارض احتمالی ناشی از مصرف سماق در گروه مداخله (کپسول سماق) چه می باشد؟ |

### *جدول متغيرها*

| **نام متغیر** | **نوع متغیر** | **مقیاس متغیر** | **نقش متغیر** | **تعریف کاربردی** | **واحد اندازه گیری** |
| --- | --- | --- | --- | --- | --- |
| سن | کمی | نسبتی | زمینه‌ای |  | سال |
| سن منارک | کمی | نسبتی | زمینه‌ای |  | سال |
| شاخص توده بدنی | کمی | نسبتی | زمینه‌ای |  | کیلوگرم بر مترمربع |
| شغل | کیفی | اسمی | زمینه‌ای |  | خانه‌دار/شاغل در منزل/ شاغل خارج از منزل |
| سطح تحصیلات | کیفی | رتبه‌ای | زمینه‌ای |  | ابتدایی/راهنمایی/دبیرستان/ دیپلم/ دانشگاهی |
| کفایت درآمد ماهیانه برای هزینه زندگی | کیفی | رتبه‌ای | زمینه‌ای |  | بیش از حد کفایت/ در حد کفایت/ کمتر از حد کفایت |
| قد | کمی | نسبتی | زمینه‌ای |  | سانتی متر |
| وزن | کمی | نسبتی | زمینه‌ای |  | کیلوگرم |
| تعداد بارداری در افراد متاهل | کمی | نسبتی | زمینه‌ای |  | تعداد |
| سابقه ورزش | کیفی | اسمی | زمینه‌ای |  | بله/خیر |
| شدت خونریزی | کمی | نسبتی | وابسته |  | نمره پرسشنامه |
| مدت خونریزی | کمی | نسبتی | وابسته |  | نمره پرسشنامه |
| مصرف کپسول سماق | کیفی | اسمی | مستقل |  | بلی/خیر |
| عوارض احتمالی سماق | کیفی | اسمی | وابسته | هرگونه عارضه احتمالی | دارد/ ندارد |

### *روش کار*

× پژوهشگر محترم، با مراجعه به سايت به آدرس (https://www.equator-network.org/ ) استانداردهاي انواع پروژه ها را مطالعه و رعايت نماييد و ترجيعاٌ در قسمت پيوست ها چک ليست مربوطه را آپلود نماييد. بديهي است رعايت استانداردها روند تصويب طرح را تسهيل مي کند.

| **عنوان** | **متن** |
| --- | --- |
| جامعه مورد مطالعه /نحوه نمونه گیری و فرمول تعیین حجم نمونه | جامعه مطالعه: زنان متاهل و دختران مجرد 18 تا 45 ساله شهرستان بابل  معیار ورود:  سن زنان بین 18 تا 45 سال  خونریزی قاعدگی بیش از 80 سی سی در هر سیکل قاعدگی، براساس چارتPBAC  سیکل قاعدگی منظم 21 روز تا 42 روز  توانایی خواندن و نوشتن  معیار عدم ورود:  وجود کیست تخمدان تشخیص داده شده  پولیپ آندومتر  اختلالات نئوپلازی  سیکل نامنظم قاعدگی  وجود بیماری مزمن (فشار خون، دیابت کنترل نشده، کلیوی، کبدی، اختلالات قلبی عروقی، مشکلات تیروئیدی)  مصرف داروی ضد انعقاد خون  مصرف سیگار و مشروبات الکلی  ضخامت غیرطبیعی آندومتر تشخیص داده شده با سونوگرافی  مصرف داروهای هورمونی ضد بارداری  سابقه سرطان در خانواده درجه 1  فیبرم رحمی بزرگتر از 3 سانتی متر شناخته شده ( از نوع ساب موکوسال و اینترا مورال)  حساسیت و آلرژی به سماق  پیروی از یک رژیم خاص طی 3 ماه گذشته  خونریزی بسیار شدیدی که نیازمند بستری می باشند  معیار خروج:  پایبندی کمتر از 80 درصد به درمان  بارداری یا شیردهی  عدم تمایل به ادامه شرکت در مطالعه  **حجم نمونه:**  با توجه به اینکه برای تعیین حجم نمونه مطالعه مشابه ای یافت نشد. بنابراین با نظر متخصصین بالین اختلاف میانگین حداقل 10 درجه (میزان خونریزی با معیار چارت هیگام) در گروه مداخله و کنترل قابل توجه می باشد. با فرض تساوی انحراف معیار برابر 5/6 و اندازه اثر 80/0و سطح اطمینان 95% ، توان آزمون 90% (با استفاده از نرم افزار Gpower) مقدار حجم نمونه 27 نفر در هر گروه براورد می گردد و با احتمال 15 درصد ریزش 30 نفر در هر گروه تخمین زده می شود.  **روش نمونه گیری:**  این مطالعه به‌صورت یک کارازمایی بالینی دوسویه کور با گروه کنترل و به ‌صورت موازی انجام می‌شود. نمونه‌گیری به‌ صورت در دسترس انجام و تخصیص ان به صورت تصادفی به گروه مداخله و دارونما خواهد بود. از روش تصادفی سازی بلوکی (Block Randomization) با بلوک‌های ده‌تایی استفاده خواهد شد. سپس شرکت‌کنندگان بر طبق پروتکل تقسیم تصادفی بلوکی (تولیدشده توسط نرم‌افزار Random Allocation online) به یکی از دو گروه 1 با نسبت 1:1 تخصیص داده خواهند شد، به‌نحوی‌که محقق نمی‌تواند پیش‌بینی کند نفر بعد در کدام گروه از مداخله قرار خواهد گرفت.  **لیست تصادفی سازی بر اساس خروجی نرم‌افزار:**    پنهان سازی (Allocation concealment)  برای پیشگیری از تورش انتخاب، تخصیص تصادفی به‌صورت پنهان انجام می‌شود. با توجه به حجم نمونه، تعدادی پاکت نامه تهیه و توالی های تصادفی روی کارت‌هایی ثبت می‌شوند که سپس در پاکت‌های نامه به ترتیب قرار می‌گیرند. به منظور حفظ توالی تصادفی، روی سطح خارجی پاکت‌ها نیز شماره‌گذاری می‌شود. در نهایت، درب پاکت‌ها چسبانده و به ترتیب در جعبه‌ای قرار می‌گیرند. هنگام ثبت‌نام شرکت‌کنندگان، طبق ترتیب ورود افراد واجد شرایط، یکی از پاکت‌ها باز شده و گروه تخصیص یافته آن شرکت‌کننده مشخص می‌شود. این مرحله پنهان‌سازی توسط فردی که در تحقیق نیست، انجام می‌شود. اجرای فرآیند تخصیص تصادفی: برای کاهش تورش ممکن، فردی که توالی تصادفی را تولید می‌کند، با افرادی که معیارهای ورود و خروج را بررسی و شرکت‌کنندگان را به گروه‌ها تخصیص می‌دهند، متفاوت است. فرد ایجادکننده برنامه تصادفی از سایر محققان مجزا خواهد بود. همچنین کدگزاری دارو توسط فردی انجام می شود که در نمونه گیری مشارکت ندارد.  کورسازی (Blinding)  در این مطالعه محقق، بیمار و فردی که آنالیز داده ها را انجام می دهد نسبت به نوع داروها کور هستند. |
| روش جمع آوری داده ها و نحوه اجرای مطالعه | نحوه اجرای مطالعه:  جهت انجام این کارآزمایی بالینی، پس از تصویب طرح در دانشگاه علوم پزشکی بابل و کسب مجوز از کمیته اخلاق دانشگاه و ثبت در سامانه IRCT، پژوهشگر به کلینیک طب سنتی، مطب یکی از متخصصین زنان و خوابگاه دختران دانشگاه علوم پزشکی بابل مراجعه کرده و بعد از توضیح اهداف تحقیق و کسب رضایت آگاهانه کتبی از افرادی که معیارهای ورود به مطالعه را دارند (از جمله خونریزی قاعدگی بیشتر از 80 سی سی بر اساس چارت هیگام)، اقدام به نمونه گیری آسان خواهد کرد. سپس نمونه ها بر اساس جدول اعداد تصادفی در دو گروه مداخله (کپسول سماق) و کنترل (دارونما) قرار می گیرند.  روش تهیه کپسول ها به این صورت است که عصاره آبی (درجه A++) میوه سماق از شرکت گیاه کالا تهیه و میزان فلاونوئیدهای کل تعیین می‌شود. سپس مقدار مشخصی از عصاره (معادل ۵۰۰ میلی‌گرم میوه خشک سماق) توسط داروساز طب سنتی در کپسول ریخته خواهد شد. کپسول های دارونما، حاوی آویسل است که از شرکت بهبودشیمی با برند گلوریا خریداری و به اندازه 500 میلی گرم توسط متخصص داروساز طب سنتی در کپسول ها ریخته خواهد شد. سپس تعداد 42 کپسول سماق یا دارونما برای مصرف سه سیکل قاعدگی در ظرف مخصوص ریخته شده و روی ظرف ها کد گزاری انجام می شود.  نمونه ها طی 7 روز اول قاعدگی روزانه دو عدد کپسول به فاصله 12 ساعت را مصرف خواهند کرد. کپسول ها سه سیکل قاعدگی باید مصرف شوند. برای هر دوره قاعدگی جدول هیگام به شرکت کنندگان جهت تکمیل تحویل داده خواهد شد. همچنین در صورت لزوم مصرف مفنامیک اسید و آهن به هر دو گروه تجویز می شود و میزان مصرف این دو دارو در هر سیکل ارزیابی خواهد شد.  ابزارهای مورد استفاده در مطالعه شامل: پرسشنامه مشخصات فردی-اجتماعی است که قبل از مطالعه و جدول هیگام (چارت بصری PBAC) است که قبل از مطالعه و طی سه سیکل قاعدگی توسط شرکت کنندگان تکمیل خواهد شد. به همراه توضیحات جهت تکمیل در اختیار شرکت کنندگان قرار خواهد گرفت. |
| ابزارها | -چارت بصری PBAC: برای ارزیابی میزان خونریزی استفاده می‌شود. این جدول شامل چارت ارزیابی دیداری خون می‌باشد که یک روش ساده غیرآزمایشگاهی برای تشخیص خونریزی است. این سیستم امتیازدهی عمومی، دیدن نوارهای بهداشتی، تامپون‌ها و ظاهر شدن لخته‌ها را مبنا قرار میدهد.این جدول دو بعد تصویری داردکه در ردیف افقی تعداد روزهای قاعدگی و در ردیف عمودی پدهای آغشته به خون را در سه درجه خفیف (موارد کمتر آلوده به خون نواربهداشتی در حد لکه بینی) امتیاز 1، درجه متوسط امتیاز 5 و درجه شدید آغشتگی پد به خون امتیاز 20 درنظر گرفته شد. همچنین برای دفع لخته‌های کوچک (تقریبا به قطر یک سکه 2 ریالی) امتیاز 1 و برای دفع لخته‌های بزرگ (تقریبا به قطر یک سکه 100 ریالی) امتیاز 5 و برای هر جریان خون (آلوده شدن لباس زیر یا جاری شدن خون در دستشویی) امتیاز 5، در نظر گرفته شد.در پایان قاعدگی، هر علامت را در امتیاز مربوطه ضرب میکنند و اعداد به دست آمده را با هم جمع کرده و امتیاز کلی محاسبه می‌شود. امتیاز 100 یا بیشتر معادل خونریزی بیشتر از 80 میلی‌لیتر است. چارت هیگام جهت سنجش مقدار خونریزی قاعدگی ابزاری روا است و در مطالعه باغداری و همکاران (2006) و حیدری و همکاران(2008) مورد استفاده قرار گرفته است. پایایی آن نیز به روش آزمون-آزمون مجدد محاسبه شد، بدین صورت که پژوهشگر آزمون را برای 10 نفراز دانشجویان، 2 بار به فاصله یک هفته اجرا کرد و پایایی آن 85/0 =r و 011/0 =p مورد تایید قرار گرفت (39).‬‬‬‬‬‬‬‬‬‬‬‬‬‬‬‬‬‬‬‬‬‬‬‬‬‬‬‬‬‬‬‬‬‬‬‬‬‬‬‬‬‬‬‬‬‬‬‬‬‬‬‬‬  اطلاعات دموگرافیک از قبیل سن، نمایه توده بدنی، سطح تحصیلات، محل سکونت، وضعیت اشتغال، رضایت از درامد زندگی، اطلاعات مامایی مانند: تعداد بارداری، زایمان و سقط، روش جلوگیری از بارداری  اطلاعات خونریزی غیرطبیعی: شامل، الگوی سیکل قاعدگی (نظم در قاعدگی، فاصله بین قاعدگی‌ها، طول مدت خونریزی، درد زمان قاعدگی٬ میزان خونریزی) می باشد. |
| روش تجزیه تحلیل آماری | برای تجزیه و تحلیل آماری پس از گردآوري داده ها، اطلاعات توصیفی زنان با استفاده از شاخص مرکزی میانگین و شاخص پراکندگی انحراف معیار و یا به شکل تعداد و درصد گزارش خواهد شد. به منظور بررسی آزمون های آماری مرتبط ابتدا نرمالیتی داده ها با استفاده از آزمون کولموگروف اسمیرنوف مورد بررسی قرار خواهد گرفت. برای بررسی همبستگی بین متغیرهای کمی از آزمون همبستگی پیرسون، و ارتباط دو متغیر کیفی از آزمون کای اسکوئر (Chi-square) و در صورت برقرار نبودن شرایط آزمون کای اسکوئر از تست فیشر استفاده خواهد شد. برای بررسی نتایج مورد نظر در گروهها، به منظور بررسی ارتباط بین یک متغیر کمی در دو وضعیت وابسته از آزمون تی زوجی و در دو وضعیت مستقل نیز از آزمون تی مستقل استفاده خواهد شد. همچنین برای مقایسه میزان خونریزی قاعدگی و مدت خونریزی بعد از مداخله در دو گروه آزمون ANOVA Repeated measure مد نظر قرار خواهدگرفت. همچنین اختلاف میانگین (Mean Difference) و فاصله اطمینان (Confidence of Interval) 95 درصد براي نشان دادن اندازه اثر استفاده می گردد. تمامی آنالیز ها بوسیله نرم افزار SPSS نسخه 22 و سطح معنی داری (05/0>P) در نظر گرفته خواهد شد. |
| **مشکلات اجرایی احتمالی** | جلب اعتماد خانم‌های واجد شرایط جهت ورود به مطالعه  عدم همکاری افراد مورد پژوهش در استفاده منظم از داروها  عدم مراجعه مجدد زنان مطالعه |

**ملاحظات اخلاقی**

- پس از تایید کمیته اخلاق دانشگاه علوم پزشکی بابل این مطالعه صورت خواهد گرفت.
- پژوهشگر با اخذ معرفي نامه كتبي از دانشگاه علوم پزشکی بابل و ارائه آن به مسئولان و مديران محيط پژوهش، مجوز لازم را از آنان كسب خواهد شد.
- هدف از انجام پژوهش براي هر يك از واحدهاي مورد پژوهش و مسئولين محيط مطالعه توضيح داده خواهد شد.
- واحدهاي مورد پژوهش جهت شركت يا عدم شركت در پژوهش حق انتخاب داشته و در صورت شركت در پژوهش رضايت نامه ي كتبي اخذ خواهد شد.
- در استفاده از كتب و منابع علمي و ترجمه مطالب، رعايت حقوق ادبي و امانت به عمل خواهد آمد.
- در صورت تمایل شرکت کنندگان، یافته های پژوهش در اختیار آن ها قرارخواهد گرفت.
- به واحدهای مورد پژوهش اطمینان داده خواهد شد كه اطلاعات شخصی و مندرجات پرسشنامه آن‌ها نزد پژوهشگر محفوظ خواهد ماند.

### *فهرست منابع و ماخذ*

1.	خدابخش, رودی, فیض‌آبادی. نقش تغذیه در درمان بیماران مبتلا به خونریزی زیاد قاعدگی از دیدگاه طب سنتی ایرانی و طب نوین. مجله دانشکده پزشکی اصفهان. 2019;36(512):1675-83.

2.	Borzutzky C, Jaffray J. Diagnosis and management of heavy menstrual bleeding and bleeding disorders in adolescents. JAMA pediatrics. 2020;174(2):186-94.

3.	Ibrahim PM, Samwel EL. Prevalence of Heavy Menstrual Bleeding and Its Associated Factors Among Women Attending Kilimanjaro Christian Medical Centre In Northern Eastern, Tanzania: A Cross-Sectional Study. The East African Health Research Journal. 2023;7(1):1.

4.	Fritz MA, Speroff L. Clinical Gynecologic endocrinology and infertility. Translated by: Valadan M, Faghani Jadidi N Tehran: Argomand book. 2013:70-311.

5.	Kaunitz AM, Barbieri RL, Levine D, Falk SJ. Approach to abnormal uterine bleeding in nonpregnant reproductive-age women. Internet) Walthman (MA): UpToDate. 2015.

6.	Ash T. Massive transfusion protocol. Archives of Hematology Mini Reviews and Reviews. 2023;8(1):004-7.

7.	Kulkarni R. Improving care and treatment options for women and girls with bleeding disorders. European journal of haematology. 2015;95:2-10.

8.	Bradley LD, Gueye N-A. The medical management of abnormal uterine bleeding in reproductive-aged women. American journal of Obstetrics and Gynecology. 2016;214(1):31-44.

9.	Rodriguez MB, Lethaby A, Jordan V. Progestogen‐releasing intrauterine systems for heavy menstrual bleeding. Cochrane Database of Systematic Reviews. 2020(6).

10.	Isono W, Tsuchiya A, Okamura A, Honda M, Saito A, Tsuchiya H, et al. Unilateral uterine artery embolization and Bakri tamponade balloon insertion in the treatment of acute puerperal uterine inversion: a case report. Journal of Medical Case Reports. 2022;16(1):190.

11.	Kaganov H, Ades A. Uterine fibroids: investigation and current management trends. Australian Family Physician. 2016;45(10):722-5.

12.	تن‌ساز, مژگان د, کورابی نا, قرائتی, مرضیه د, مکبری‌نژاد, et al. پروتکل درمانی خونریزی غیرطبیعی رحمی در سنین باروری از دیدگاه طب ایرانی. مجله زنان، مامایی و نازایی ایران. 2023;25(11):62-72.

13.	Falahat F, Tavakkoli M, Mokaberinejad R, Ayati S, Feyzabadi Z. Natural treatments of oligomenorrhea based on persian medicine. The Iranian Journal of Obstetrics, Gynecology and Infertility. 2018;21(Supplement):55-66.

14.	Fesharaki G, Zafarghandi N. Comparison of effects of pomegranate peel and Mefenamic acid consumption on the treatment of Menorrhagia: A Triple-Blind Randomized Controlled Trial. 2019.

15.	Ashteany E-h, Vahid Dastjerdi M, Tabarrai M, Nejatbakhsh F, Sadati Lamardi SN, Rahmani A, et al. Effectiveness of Persian Golnar on Excessive Menstrual Bleeding in Women with Abnormal Uterine Bleeding, Compared to Tranexamic Acid: A Triple-Blind, Randomized Equivalence Trial. Evidence-Based Complementary and Alternative Medicine. 2023;2023.

16.	Salsali G. Food recommendations for abnormal uterine bleeding in Traditional Iranian medicine on the basis of Hemorheology. Int J Ayurved Med. 2019;10(1):1-6.

17.	Mozaffarian V. A dictionary of Iranian plant names: Latin, English, Persian: Farhang Mo'aser; 1996.

18.	Ghahreman A, Okhovvat A. Matching the old medicinal plant names with scientific terminology. vol. 1. Farsi ed. Tehran University Press, Iran; 2004.

19.	Ahmad H, Ahmad F, Hasan I, Ahmad S. Unani description of Sumaq (Rhus coriaria Linn.) and its scientific report. Global Journal of Medical Research. 2013;13(7):75-8.

20.	M A. Tazkiratul alaj. Tehran: Research Institute for Islamic and Complementary medicine; 1999.

21.	Zargham H, Zargham R. Tannin extracted from Sumac inhibits vascular smooth muscle cell migration. McGill Journal of Medicine: MJM. 2008;11(2):119.

22.	Mohammadi S, Zarei M, Zarei MM, Salehi I. Effect of hydroalcoholic leaves extract of Rhus Coriaria on pain in male rats. Anesthesiology and pain medicine. 2016;6(1).

23.	Mosaffa-Jahromi M, Kiani H. Alshamel fi Sana’at Tebbi’at: A Comprehensive Book on the Materia Medica. Iranian Journal of Medical Sciences. 2016;41(3 Suppl):S20.

24.	Razi B. Kholasat ol-Tajarob. The Institute for Medical History-Islamic and Complementary Medicine, Tehran University of Medical Sciences, Tehran. 2000:134-53.

25.	MA A. Tibb-i Akbari (Akbari's Medicine). Qom: Jalal al-Din Publications. 2008;2:690-5.

26.	Ibn-e-Sina A. Al-qanun fit-tib [The canon of medicine]. Beirut, Lebanon: Alaalami Beirut lib Press; 2005.

27.	Aghili Shirazi S. Makhzan Al-adviyah (the Storehouse of medicaments). Tehran: Tehran University of Medical Sciences. 2009.

28.	Capcarova M, Slamecka J, Abbas K, Kolesarova A, Kalafova A, Valent M, et al. Effects of dietary inclusion of Rhus coriaria on internal milieu of rabbits. Journal of animal physiology and animal nutrition. 2012;96(3):459-65.

29.	Khodabakhsh M, Mahmoudinia M, Mousavi Bazaz M, Hamedi SS, Hoseini SS, Feyzabadi Z, et al. The effect of plantain syrup on heavy menstrual bleeding: A randomized triple blind clinical trial. Phytotherapy Research. 2020;34(1):118-25.

30.	Payranj M, Moradi M, Rakhshandeh H, Ghavami V, Farahi SMM, Najafabadi MG, et al. Investigating the Impact of Sumac Capsules on Postpartum Bleeding among Women at Risk of Bleeding: a three-blind randomized clinical trial. Journal of Pharmacopuncture. 2024;27(4):287.

31.	Amirsasan R, Zakeri N, Gargari BP, Alizadeh S. The effect of anti-inflammatory properties of sumac powder (Rhus coriaria L.) along with ferrous sulfate on blood markers and oxidative stress of young athletic girls with non-anemic iron deficiency (NAID). Sport Sciences for Health. 2024:1-9.

32.	Momenimovahed Z, Khalajinia Z. Unwillingness to have Vaginal Delivery among Iranian Women: A Qualitative Study. Current Womens Health Reviews. 2025;21(1):E240124226088.

33.	Radman M, Kaeidi A, Dastjerdi MF, Javadi A. Effects of Pomegranate, Myrtle, Quercus Fruit, and Rhus coriaria L Extracts on Bleeding Control in Rat. Jundishapur Journal of Natural Pharmaceutical Products. 2023;18(2).

34.	Afandak F, Aryaeian N, Kashanian M, Janani L, Namizadeh T, Karimi MY, et al. Effect of sumac powder on clinical symptoms, hyperandrogenism, inflammation, blood glucose, lipid profiles in women with polycystic ovary syndrome: A double‐blind randomized clinical trial. Phytotherapy Research. 2023;37(6):2315-25.

35.	Mirenayat FS, Hajhashemy Z, Siavash M, Saneei P. Effects of sumac supplementation on metabolic markers in adults with metabolic syndrome: a triple-blinded randomized placebo-controlled cross-over clinical trial. Nutrition Journal. 2023;22(1):25.

36.	Eshaghian R, Mazaheri M, Ghanadian M, Rouholamin S, Feizi A, Babaeian M. The effect of frankincense (Boswellia serrata, oleoresin) and ginger (Zingiber officinale, rhizoma) on heavy menstrual bleeding: A randomized, placebo-controlled, clinical trial. Complementary therapies in medicine. 2019;42:42-7.

37.	Karimian Z, Sadat Z, Bahrami N, Kafaie M. Comparison of chamomile and mefenamic acid capsules in hemorrhage of menstruation. The Iranian Journal of Obstetrics, Gynecology and Infertility. 2015;18(157):11-7.

38.	Mirghafourvand M, Charandabi SMA, Javadzadeh Y, Ahmadpour P. The Effect of Vitex agnus and Flaxseed on the Menstrual Bleeding: a Randomized Controlled Trial. Journal of Advances in Medical and Biomedical Research. 2015;23(100):1-12.

39.	ساز ت, مژگان د, مریم د, ‌نژاد م, روشنک د, ادهمی, et al. بررسی تأثیر تجویز مرکب دو داروی شربت تونیک عنصلی و کپسول صمغ (فرآورده‌های طب ایرانی) بر علائم بالینی و آزمایشگاهی زنان مبتلا به سندرم تخمدان پلی‌کیستیک (مطالعه پایلوت). مجله زنان، مامایی و نازایی ایران. 2022;25(9):1-10.

1. Lipopolysaccharide
