# Supplementary material for: Sumac (Rhus coriaria L.) Capsules for the management of hypermenorrhea: Protocol for a randomized triple-blind trial
Source: PLoS One. 2026 Nov 11;21(11):e0361956. doi: 10.1371/journal.pone.0361956 (PMC13754152; doi:10.1371/journal.pone.0361956)
Supplement: Supplementary file 8 [file pone.0361956.s006.docx]

**Dear PLOS ONE Editorial Office,**

Thank you for your careful technical check of our manuscript entitled “**Sumac (Rhus coriaria L.) Capsules for the Management of Hypermenorrhea: Protocol for a Randomized Triple-Blind Trial**” (Manuscript ID: PONE-D-26-06715).

We have addressed all requested points as detailed below:

1. **SPIRIT Schedule (Figure):** The SPIRIT schedule has been re-uploaded as **Fig 1**, in accordance with PLOS ONE submission guidelines. Figure numbering, legends, and in-text citations have been amended accordingly.
2. **SPIRIT Checklist:** A completed **SPIRIT checklist** has now been uploaded as **Supporting Information**.
3. **Ethics-Approved Study Protocol:** We would like to clarify the language of the submitted study protocol in response to your request. The study protocol was originally submitted to and approved by the institutional ethics committee in **Persian**, which is therefore the official and authoritative version. In accordance with the journal’s requirements for Clinical Trial Study Protocols, we have now provided:

- The **original Persian ethics committee–approved protocol**, and
- A **complete English translation** of this protocol for editorial and reviewer assessment.

Both documents have been uploaded as Supporting Information files. The original, signed ethics committee approval letter has been provided separately as an “Other” file.

4. **Ethics-Approved Study Protocol (Duplicate Request):** Please note that this item duplicates point 3 above. The requested protocol documents have been fully provided.
5. **Ethics Approval Date in Methods Section:** The Methods section has been amended to explicitly state the **date of Ethics Committee/IRB approval**. (Ethical approval for this study was obtained from the Research Ethics Committee of Babol University of Medical Sciences (Approval Code: IR.MUBABOL.REC.1404.106) on 4 August 2025.)
6. **Ethics Approval Letter:** A copy of the original, dated, and officially stamped/signed **IRB/Ethics Committee approval letter** has been uploaded as an **“Other” file**, in line with journal requirements.

We believe that all technical requirements have now been fully satisfied. We appreciate your time and assistance, and we look forward to the next stage of editorial assessment.

Kind regards,

**Dr. Fereshteh Behmanesh**

Associate Professor, Reproductive Health, PhD.
Department of Nursing and Midwifery, Babol University of Medical Sciences.

PLOS ONE Editorial Board Member

<https://orcid.org/0000-0001-5837-4265>
