# Supplementary material for: Sumac (Rhus coriaria L.) Capsules for the management of hypermenorrhea: Protocol for a randomized triple-blind trial
Source: PLoS One. 2026 Nov 11;21(11):e0361956. doi: 10.1371/journal.pone.0361956 (PMC13754152; doi:10.1371/journal.pone.0361956)
Supplement: Supplementary file 9 [file pone.0361956.s008.docx]

**Dear Editor**

**Prof Katrien G. Janin,**

**PLOS One**

Thank you for the continued consideration of our manuscript, “***Sumac (Rhus coriaria L.) Capsules for the Management of Hypermenorrhea: Protocol for a Randomized Triple-Blind Trial***” (Manuscript ID: PONE-D-26-06715).

We sincerely appreciate the reviewers’ further comments and constructive suggestions. We have carefully addressed all remaining comments and made the corresponding revisions and clarifications in the manuscript. We believe that these revisions have further improved the clarity, methodological rigor, and completeness of the protocol.

All changes have been highlighted in the revised manuscript, and detailed responses to each reviewer comment are provided in the response letter.

Thank you for your time and consideration of our revised manuscript.

Sincerely, Corresponding author

Fereshteh Behmanesh

Associate Professor, Reproductive Health, PhD.

Department of Nursing and Midwifery, Babol University of Medical Sciences. https://orcid.org/0000-0001-5837-4265

| **Academic editor and reviewer(s)** | **Response** | **page** |
| --- | --- | --- |
| ***Editor*** | | |
| Dear Dr. Behmanesh,  Thank you for submitting your manuscript to PLOS One. After careful consideration, we feel that it has merit but does not fully meet PLOS One’s publication criteria as it currently stands. Therefore, we invite you to submit a revised version of the manuscript that addresses the points raised during the review process.  Your manuscript has now been reassessed. The reviewers recognize the considerable improvements made in response to the previous comments, yet some points remains for consideration. Please address this in your revised manuscript and point-by-point response. | Dear Editor, thank you for your guidance. We have carefully revised the entire manuscript based on all reviewers' comments and now submit the updated version. | All manuscript |
| Please include the following items when submitting your revised manuscript:  A letter that responds to each point raised by the academic editor and reviewer(s). You should upload this letter as a separate file labeled 'Response to Reviewers'.  A marked-up copy of your manuscript that highlights changes made to the original version. You should upload this as a separate file labeled 'Revised Manuscript with Track Changes'.  An unmarked version of your revised paper without tracked changes. You should upload this as a separate file labeled 'Manuscript'. | Thank you very much for your careful guidance and for providing clear instructions regarding the resubmission of our revised manuscript. We sincerely appreciate your time and the constructive approach you have taken in handling our submission.  In accordance with your request, we have prepared and will upload the following three files:  A detailed point-by-point response letter to all comments raised by the academic editor and reviewers, which we have prepared as a separate file labeled 'Response to Reviewers'.  A marked-up copy of the revised manuscript with all changes clearly highlighted using track changes, uploaded as a separate file labeled 'Revised Manuscript with Track Changes'.  A clean, unmarked version of the revised manuscript without any tracked changes, uploaded as a separate file labeled 'Manuscript'.  Once again, thank you for your kind attention and valuable guidance. | ------ |
| If applicable, we recommend that you deposit your laboratory protocols in protocols.io to enhance the reproducibility of your results. Protocols.io assigns your protocol its own identifier (DOI) so that it can be cited independently in the future. For instructions see: https://journals.plos.org/plosone/s/submission-guidelines#loc-laboratory-protocols. Additionally, PLOS ONE offers an option for publishing peer-reviewed Lab Protocol articles, which describe protocols hosted on protocols.io. Read more information on sharing protocols at https://plos.org/protocols?utm_medium=editorial-email&utm_source=authorletters&utm_campaign=protocols. | We appreciate the suggestion to deposit our protocol on protocols.io. However, as our manuscript describes a clinical trial protocol with detailed steps already fully presented in the Methods section and Supporting Information, we believe that the current level of detail is sufficient for reproducibility. We will consider protocols.io for future laboratory-based studies. | ----- |
| Please review your reference list to ensure that it is complete and correct. If you have cited papers that have been retracted, please include the rationale for doing so in the manuscript text, or remove these references and replace them with relevant current references. Any changes to the reference list should be mentioned in the rebuttal letter that accompanies your revised manuscript. If you need to cite a retracted article, indicate the article’s retracted status in the References list and also include a citation and full reference for the retraction notice. | We thank the Editor for this important reminder. We carefully reviewed the reference list and confirmed that all cited references are appropriate and that none of the cited articles has been retracted. No changes to the reference list were therefore necessary. | ----- |
| ***Reviewer #1*** | | |
| The following points remain noteworthy in the present version of the protocol:  The permitted use of over-the-counter analgesics and other co-interventions, including Fe supplementation based on participants’ subjective experience, may introduce variability in the study outcomes and affect the internal validity of the RCT.  The criterion for Fe supplementation based on a participant’s feeling of weakness appears subjective and may benefit from consideration from a scientific standpoint. | We sincerely thank the reviewer for highlighting this important methodological concern. We agree that the use of concomitant medications, particularly iron supplementation, may introduce variability in menstrual blood loss and potentially affect the internal validity of the trial.  We also agree that the previous wording regarding iron supplementation “according to the participant’s own discretion” was not sufficiently precise and could be interpreted as allowing participants to initiate iron supplementation solely on the basis of subjective symptoms such as weakness. Our intention was to permit necessary over-the-counter medications without unnecessarily restricting participants’ access to routine symptomatic care.  To address this concern, we have revised the protocol to clarify the management of concomitant medications. Analgesics and iron supplements will not be prohibited during the trial when participants use them as part of their usual care. However, their use will be systematically documented throughout the study, including the type, dose, frequency, and duration of use. Subjective weakness will not be considered a study-defined indication for iron supplementation.  Importantly, concomitant medication use will be incorporated into the planned statistical analysis. The use of analgesics and iron supplements will be recorded as time-varying covariates and considered in the linear mixed-effects model examining menstrual blood loss over time. This approach will allow us to account for potential differences in concomitant medication exposure between the intervention and placebo groups.  We have also revised the Limitations section to explicitly acknowledge the possibility that permitted concomitant medication use may introduce variability in the primary outcome, despite prospective documentation and planned statistical adjustment.  We believe these revisions provide a more standardized and scientifically rigorous approach to managing concomitant medications while maintaining participants’ access to necessary symptomatic care. | P7, Par 2& 3  P 10, Limitations |
| Although clinically significant anemia is mentioned as an exclusion criterion, baseline Hb assessment does not appear to be included, which raises a concern regarding the objective identification of anemia. | We thank the reviewer for this important observation. We apologize for any ambiguity in our previous response. The term “clinically significant anemia” was used in our previous response to describe a potential clinical consequence of severe menstrual bleeding; it was not intended to indicate a separate anemia-based exclusion criterion or a planned baseline hemoglobin assessment.  In the protocol, the relevant exclusion criterion is severe bleeding requiring hospitalization, defined as hemodynamic instability requiring intravenous fluids or blood transfusion, based on physician judgment. This criterion is intended to identify participants with clinically severe bleeding requiring medical management. Routine baseline hemoglobin assessment is therefore not part of the study procedures, as the study is not designed to screen for or quantify anemia.  Since “clinically significant anemia” is not specified as an independent exclusion criterion in the registered protocol or the manuscript, no modification to the eligibility criteria was required. We have clarified this point in our response to avoid further ambiguity and have retained the eligibility and exclusion criteria as prespecified in the registered protocol. | ----- |
| The ethical and methodological rationale for the use of a placebo as the control intervention remains a point of consideration. | We appreciate the reviewer’s continued consideration of the ethical and methodological rationale for the placebo-controlled design. We have reconsidered this issue carefully and would like to clarify an important aspect of the study population and trial context.  This trial is not designed to withhold an established treatment from women who are currently receiving or have been offered treatment for heavy menstrual bleeding (HMB). Participants will be recruited primarily from women attending healthcare facilities for reasons unrelated to HMB and from university students who report heavy menstrual bleeding but have not sought medical care or initiated a specific treatment for this condition. Thus, participants will not be asked to discontinue an existing treatment or to replace an indicated medical treatment with placebo as a condition of participation.  The placebo comparator is scientifically important because the primary outcome, menstrual blood loss measured by PBAC, is subject to substantial within-person variability and may also be influenced by expectations and participation in a clinical trial. An identical placebo therefore provides an appropriate comparator for estimating the specific contribution of sumac supplementation beyond these nonspecific effects.  Importantly, participation in the placebo group will not restrict access to necessary medical care. Participants will be monitored throughout the trial, and those who develop severe bleeding requiring hospitalization or other clinically important deterioration will be withdrawn and referred for appropriate medical evaluation and treatment. Concomitant medications required during the study will also be permitted and prospectively recorded.  We have further clarified these safeguards and the rationale for the placebo-controlled design in the revised protocol. | P6, Par 5  P9, Ethics and dissemination |
| The change in the missing-data approach from LOCF in the earlier version to multiple imputation in the current protocol represents a substantive methodological change. The corresponding status of Ethics Committee approval is therefore an important consideration. | We thank the reviewer for raising this point. We would like to clarify that the reference to the last observation carried forward (LOCF) approach in the previous version was an inadvertent error. It did not reflect a substantive change in the planned methodology or a change made after ethical approval.  The intended approach for handling missing data in this trial has been multiple imputation. Accordingly, the current version has been corrected to accurately reflect the prespecified statistical approach. This correction does not involve any change to the study intervention, comparator, eligibility criteria, participant procedures, outcomes, or participant safety, and therefore does not constitute a substantive protocol amendment requiring a change in the existing Ethics Committee approval.  We apologize for the lack of clarity in the previous version and appreciate the reviewer’s attention to this point. | ------ |
| ***Reviewer #2*** | | |
| The authors have made substantial revisions and have addressed several of my earlier comments.  In my opinion Sample size comes to 31 after Attrition Adjustment, A sample size of 30 per group may be acceptable, but it does not provide the stated 15% attrition allowance. The authors should therefore either increase the sample size to 31 per group or revise the stated attrition allowance and calculation accordingly. | Thank you for this important observation. we have revised the Methods section to clarify the attrition description. Rather than stating a rigid 15% attrition allowance, the text has been amended to reflect that the base sample size of 26 participants per group was conservatively inflated to 30 participants per group (a total of 60 participants) to account for an approximate 10%–15% anticipated loss to follow-up. | P 6, Par 3 |
| ***Reviewer #3*** | | |
| Kindly have a paragraph on the present recruitment status of the Trial. As the trial has already been initated, the present recruitment status may be updated. | We thank the reviewer for this helpful suggestion. We have added a paragraph to the revised manuscript reporting the current recruitment status of the trial. At the time of manuscript revision, 20 participants had been recruited and randomized, and recruitment remains ongoing. | P 5, Par 3 |
| ***Reviewer #4*** | | |
| Dear authors, all the reviewer comments have been looked into, and the article has been well crafted. | We sincerely thank the reviewer for the positive and encouraging comments and for recognizing our efforts to improve the manuscript. We greatly appreciate the reviewer’s careful evaluation and constructive feedback. | ---- |
| ***Reviewer #6*** | | |
| All statistical and methodological comments raised by the reviewers have been adequately addressed, and the manuscript has been significantly improved in clarity, rigor, and completeness. | We sincerely thank the reviewer for the positive and encouraging assessment of our revised manuscript. We greatly appreciate the reviewer’s careful evaluation and recognition of the improvements made in the statistical and methodological aspects of the study. | ----- |
